# Supplementary material for: The microcephaly-associated transcriptional regulator AUTS2 cooperates with Polycomb complex PRC2 to produce upper-layer neurons in mice
Source: EMBO J. 2025 Jan 15;44(5):1354–78. doi: 10.1038/s44318-024-00343-7 (PMC11876313; doi:10.1038/s44318-024-00343-7)
Supplement: Supplementary file 1 — Appendix [file 44318_2024_343_MOESM1_ESM.pdf]

Appendix Figures for

The microcephaly-associated transcriptional regulator AUTS2 cooperates with Polycomb complex PRC2 to produce upper-layer neurons in mice

Kazumi Shimaoka, Kei Hori, Satoshi Miyashita, Yukiko U. Inoue, Nao K.N. Tabe, Asami Sakamoto, Ikuko Hasegawa, Kayo Nishitani, Kunihiko Yamashiro, Saki F. Egusa, Shoji Tatsumoto, Yasuhiro Go, Manabu Abe, Kenji Sakimura, Takayoshi Inoue, Takuya Imamura, Mikio Hoshino*

*Correspondence should be addressed to M.H. (hoshino@ncnp.go.jp)

Table of Content:

Appendix Figure S1	….. Page 2–3
Appendix Figure S2	….. Page 4–5
Appendix Figure S3	….. Page 6–7
Appendix Figure S4	….. Page 8–9
Appendix Figure S5	….. Page 10
Appendix Figure S6	….. Page 11–12
Appendix Figure S7	….. Page 13
Appendix Figure S8	….. Page14–15
Appendix Figure S9	….. Page 16–17
Section S1	….. Page 18
Section S2	….. Page 19

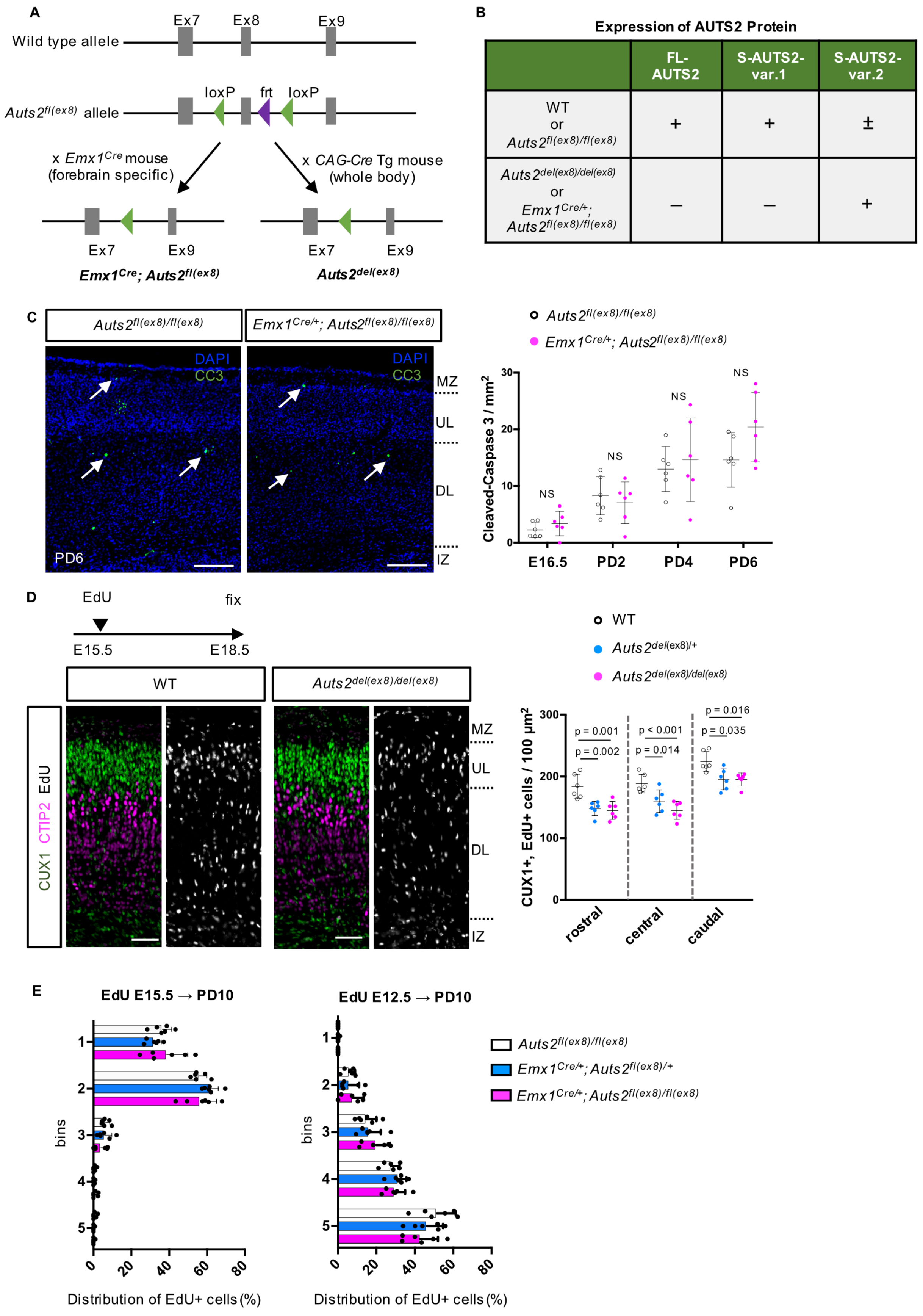

Appendix Fig. S1. AUTS2 regulates the production of neurons but not apoptosis.

(A) The genomic structure of the *Auts2* gene in each allele.

(B) The expression profile of AUTS2 protein variants in each genotype.

(C) Immunostaining for cleaved-caspase3 (CC3, green) and DAPI (blue) on the cerebral cortical sections of *Auts2*$^{fl(ex8)/fl(ex8)}$ and *Emx1*$^{Cre/+}$; *Auts2*$^{fl(ex8)/fl(ex8)}$ mice at PD6. Arrows indicate CC3^{+} cells. The graph shows the number of CC3^{+} cells in the cerebral cortex in the *Auts2*$^{fl(ex8)/fl(ex8)}$ and *Emx1*$^{Cre/+}$; *Auts2*$^{fl(ex8)/fl(ex8)}$ mice at E16.5, PD2, PD4, and PD6. MZ, marginal zone; UL, upper-layer; DL, deep-layer; IZ, intermediate zone.

(D) One pulse EdU labeling was performed in E15.5 WT, *Auts2*$^{del(ex8)/+}$, and *Auts2*$^{del(ex8)/del(ex8)}$ mice. The embryos were analyzed at E18.5 by triple-staining with CUX1 (green), CTIP2 (magenta), and EdU (white). Representative images show the rostral sections of cerebral cortices in WT and *Auts2*$^{del(ex8)/del(ex8)}$ mice. The graph shows the number of CUX1/EdU-double positive cells in a 100 µm^{2} area of WT, *Auts2*$^{del(ex8)/+}$, and *Auts2*$^{del(ex8)/del(ex8)}$ cerebral cortex.

(E) Distribution of EdU^{+} cells labeled at E15.5 (left) and E12.5 (right) in *Auts2*$^{fl(ex8)/fl(ex8)}$, *Emx1*$^{Cre/+}$; *Auts2*$^{fl(ex8)/+}$ and *Emx1*$^{Cre/+}$; *Auts2*$^{fl(ex8)/fl(ex8)}$ cerebral cortices at PD10. The graphs show the percentage of EdU^{+} cells at the rostral point in the five bins relative to total EdU^{+} cells, as indicated in Figures 1F and 1G. Bin 1 includes the marginal zone and upper layer, bin 2 is the upper layer, bin 3 is the upper and deep layers, and bins 4–5 are the deep layer.

Data are presented as the mean ± SD (N = three mice, six sections). NS, not significant, Student's t-test (C) and One-way ANOVA with Dunnett's post-hoc test or Kruskal–Wallis test (D, E). Scale bars, 200 µm (C) and 100 µm (D).

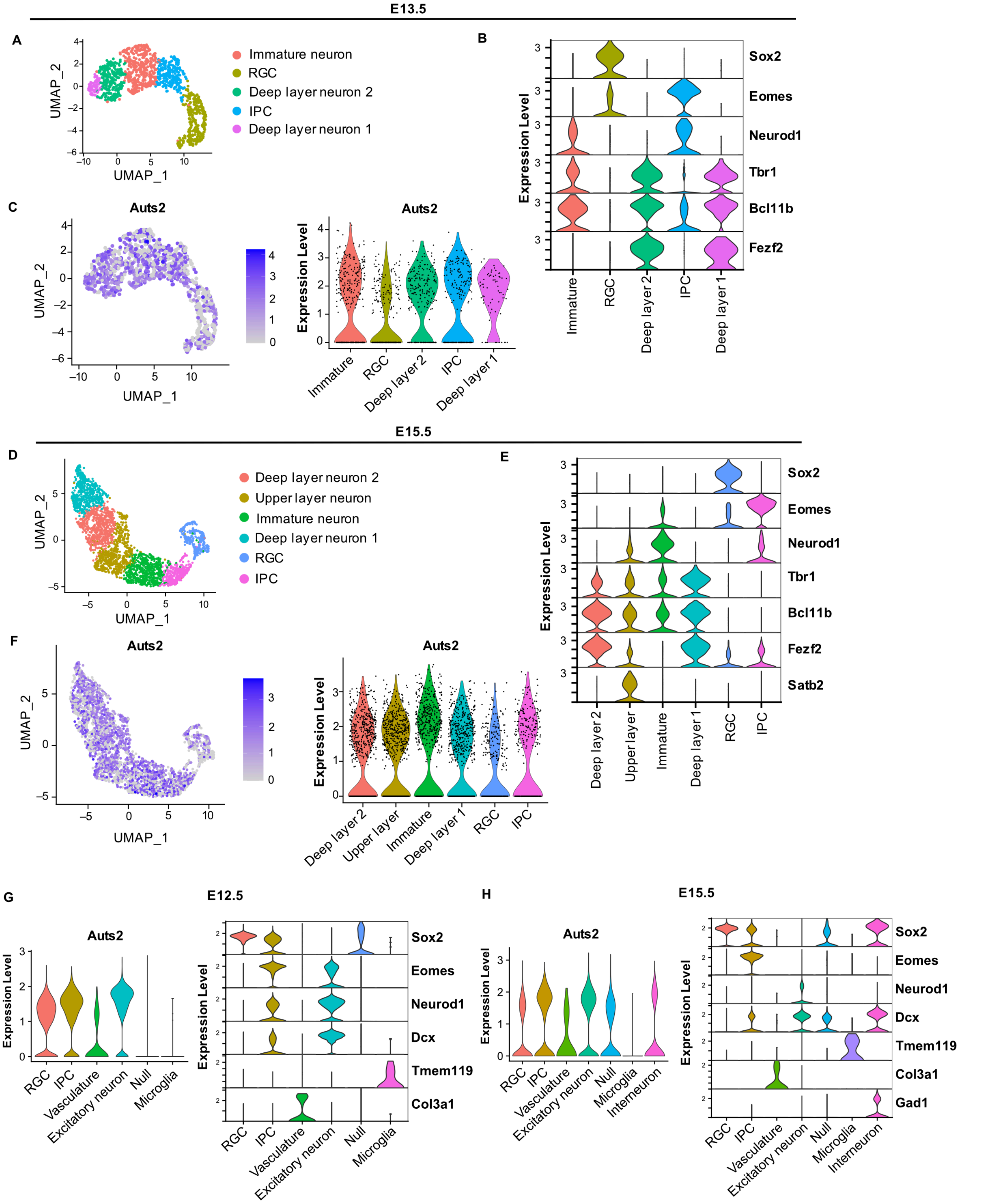

Appendix Fig. S2. Expression of *Auts2* transcripts in developing mouse cortex.

(A-F) Data of WT cerebral cortical cells at E13.5 (A–C) and E15.5 (D–F) from previously published scRNA-seq data (Yuzwa *et al*, 2017; Data ref: Yuzwa *et al*, 2017) were processed with the computational pipeline (Seurat). (A, D) UMAP-based

dimensional reduction and cluster analysis grouped five users for E13.5 (A) and six clusters for E15.5 (D). (B) Violin plots showing the expression levels of representative marker genes (*Sox2*, *Eomes*, *Neurod1*, *Tbr1*, *Bcl11b,* and *Fezf2*). Each cluster was annotated based on these expressions. (C, F) Expression levels of *Auts2* transcripts are shown in feature and violin plots at E13.5 (C) and E15.5 (F). (E) Violin plots showing the expression levels of representative marker genes (*Sox2*, *Eomes*, *Neurod1*, *Tbr1*, *Bcl11b*, *Fezf2,* and *Satb2*). Each cluster was annotated based on these expressions.

(G, H) Violin plots showing the expression levels of *Auts2* or marker genes for each cluster in the WT cerebral cortex at E12.5 (G) and E15.5 (H) from previously published scRNA-seq dataset (Di Bella *et al*, 2021; Data ref: Di Bella *et al*, 2021).

The expression of *Auts2* was significantly higher in IPCs than in RGCs at E12.5 (*Padj*=2.59E-63 (G)), E13.5 (*Padj*=5.50E-07 (C)), and E15.5 (*Padj*=1.99E-05 (F), *Padj*=2.04E-84 (H)).

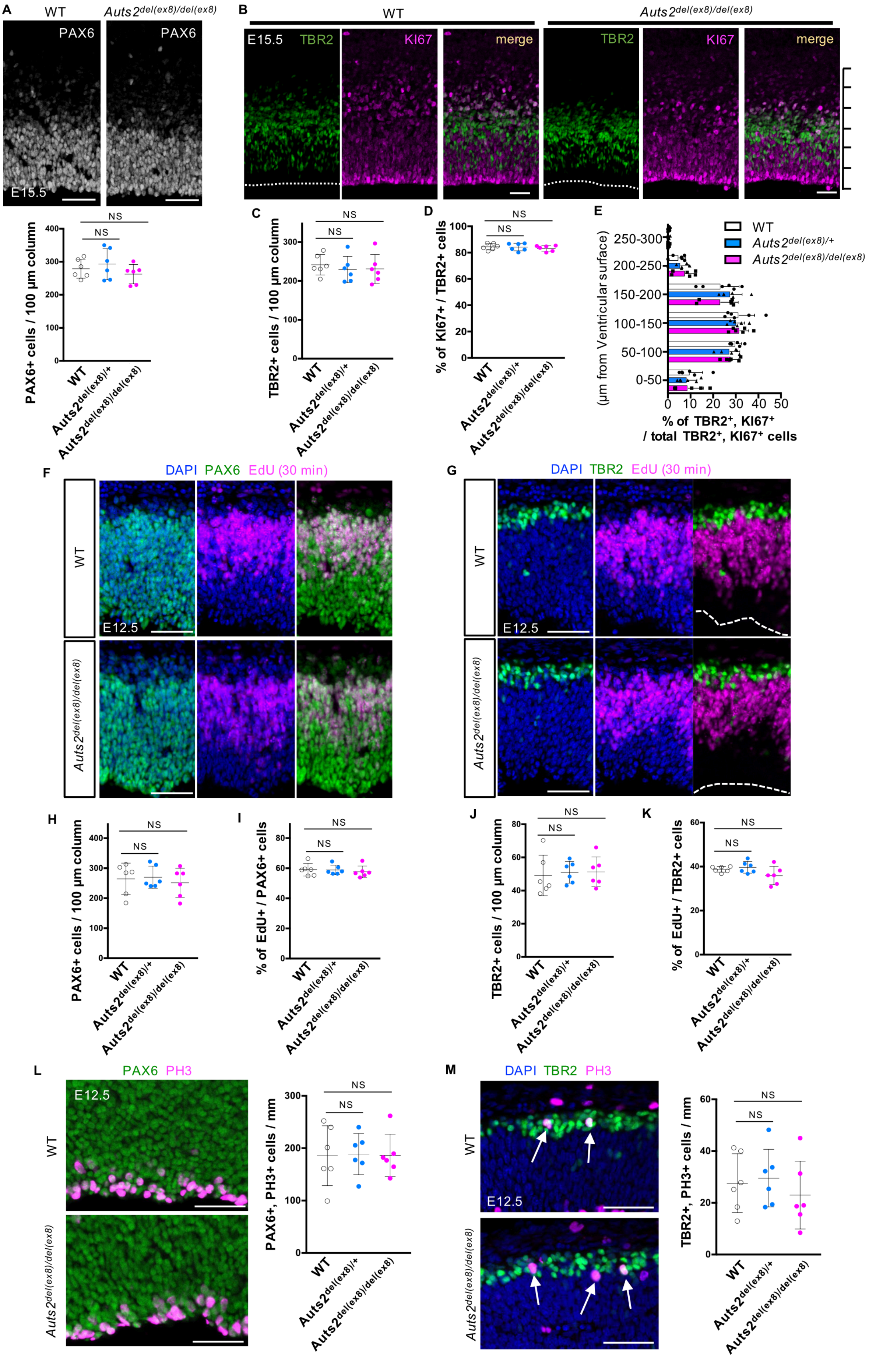
A
WT
Auts2del(ex8)/del(ex8)
PAX6
E15.5
PAX6+ cells / 100 µm column
B
E15.5 TBR2 KI67 merge
C
TBR2+ cells / 100 µm column
D
% of KI67+ / TBR2+ cells
E
WT
Auts2del(ex8)/+
Auts2del(ex8)/del(ex8)
(µm from Ventricular surface)
% of TBR2+, KI67+ / total TBR2+, KI67+ cells
F
DAPI PAX6 EdU (30 min)
E12.5
G
DAPI TBR2 EdU (30 min)
H
PAX6+ cells / 100 µm column
I
% of EdU+ / PAX6+ cells
J
TBR2+ cells / 100 µm column
K
% of EdU+ / TBR2+ cells
L
PAX6 PH3
PAX6+, PH3+ cells / mm
M
DAPI TBR2 PH3
TBR2+, PH3+ cells / mm
NS

Appendix Fig. S3. Loss of *Auts2* does not affect the proliferation of RGCs and IPCs at E12.5.

(A) Representative images of immunostaining for PAX6 in WT and *Auts2*$^{del(ex8)/del(ex8)}$ cortical sections at E15.5. The graph shows the number of PAX6^{+} cells within a 100 µm wide field.

(B–E) Representative images of immunostaining for TBR2 (green) and KI67 (magenta) in WT and *Auts2*$^{del(ex8)/del(ex8)}$ cortical sections at E15.5. The graphs show the number of TBR2^{+} cells within a 100 µm wide field (C), the ratio of KI67^{+} cells in TBR2^{+} cells (D), and the distribution of TBR2^{+} and KI67^{+} cells (E). Bins were set up parallel to the ventricular surface, separated every 50 µm (E).

(F–K) EdU was administered intraperitoneally to pregnant mice 30 min before sacrifice at E12.5. (F) Representative staining images for DAPI (blue), PAX6 (green) and EdU (magenta) in WT and *Auts2*$^{del(ex8)/del(ex8)}$ cortical sections. (G) Representative staining images for DAPI (blue), TBR2 (green) and EdU (magenta) in WT and *Auts2*$^{del(ex8)/del(ex8)}$ cortical sections. (H, J) The graphs show the number of PAX6^{+} (H) and TBR2^{+} (J) cells within a 100 µm wide field. (I, K) The graphs show the percentage of EdU^{+} cells in PAX6^{+} cells (I) and TBR2^{+} cells (K).

(L) Representative immunostaining images for PAX6 (green) and PH3 (magenta) in WT and *Auts2*$^{del(ex8)/del(ex8)}$ cortical sections at E12.5. The graph shows the number of PAX6^{+} and PH3^{+} cells on the ventricular surface in WT, *Auts2*$^{del(ex8)/+}$, and *Auts2*$^{del(ex8)/del(ex8)}$ mice.

(M) Representative immunostaining images for DAPI (blue), TBR2 (green) and PH3 (magenta) in WT and *Auts2*$^{del(ex8)/del(ex8)}$ cortical sections at E12.5. The graph shows the number of TBR2^{+} and PH3^{+} cells in WT, *Auts2*$^{del(ex8)/+}$, and *Auts2*$^{del(ex8)/del(ex8)}$ mice. Arrows indicate TBR2^{+} and PH3^{+} cells.

The number of cells was quantified at the rostral point. Data are presented as the mean ± SD (N = three mice, six sections). NS, not significant, One-way ANOVA with Dunnett's post-hoc test. Scale bars, 50 µm.

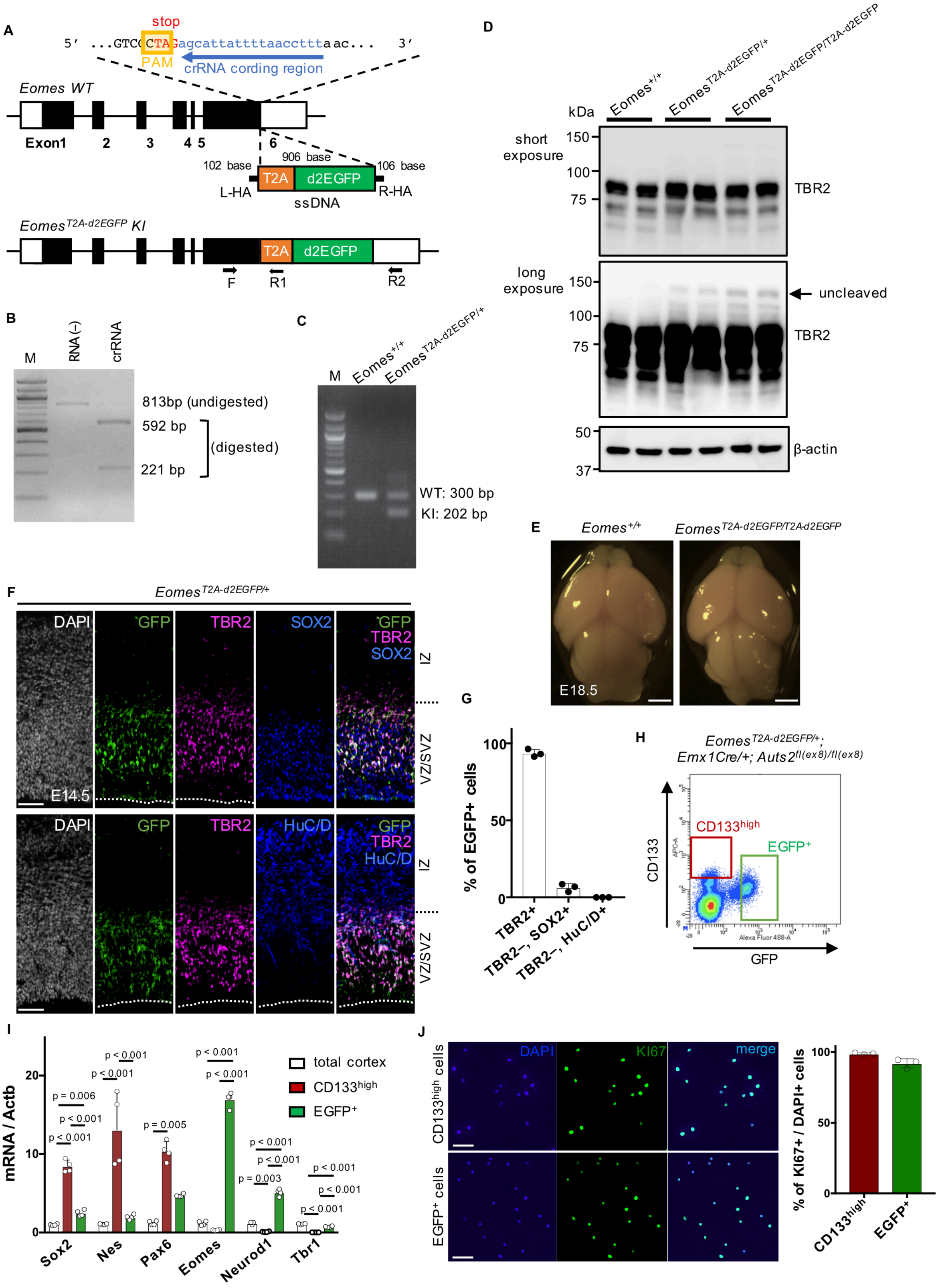

Appendix Fig. S4. Generation of *Eomes*$^{T2A\text{-}d2EGFP}$ knock-in mouse.

(A) Targeting strategy of CRISPR/Cas9 method for the generation of *Eomes*$^{T2A\text{-}d2EGFP}$ knock-in mouse. The DNA sequence for T2A-d2EGFP is inserted between the last amino acid and the stop codon of the *Eomes* locus.

L-HA, left homology arm; R-HA, right homology arm; ssDNA, single-stranded donor DNA; F, forward primer for genotyping;

R1 and R2, reverse primers for genotyping.

(B) *In vitro* digestion assay to check the cleavage activity of the crRNA designed in (A). Targeted PCR products of the *Eomes* locus were cleaved in the presence of the chemically synthesized guide RNA (*Eomes* crRNA/tracrRNA) combined with Cas9 protein. M, molecular marker.

(C) Genotyping for WT and *Eomes*$^{T2A\text{-}d2EGFP/+}$ (KI) mouse using primers indicated in (A).

(D) Western blotting analysis of cerebral cortical lysates in WT, *Eomes*$^{T2A\text{-}d2EGFP/+}$, and *Eomes*$^{T2A\text{-}d2EGFP/T2A\text{-}d2EGFP}$ mice at E14.5 with anti-TBR2 and anti-β-actin antibodies. A very small amount of uncleaved TBR2-T2A-d2EGFP protein was observed at the long exposure (arrow); however, most signals for TBR2 were detected around the expected size for TBR2 protein (short exposure).

(E) Whole-mount images in WT and *Eomes*$^{T2A\text{-}d2EGFP/T2A\text{-}d2EGFP}$ mouse brains at E18.5. Scale bars, 1 mm.

(F) Representative images of staining with DAPI (white), anti-GFP (green), anti-TBR2 (magenta), anti-SOX2 (upper, blue), and anti-HuC/D (lower, blue) antibodies in *Eomes*$^{T2A\text{-}d2EGFP/+}$ cortical sections at E14.5. IZ, intermediate zone; SVZ, subventricular zone; VZ, ventricular zone. Scale bars, 50 µm.

(G) The graph shows the percentage of indicated cells among GFP$^+$ cells. Data are presented as the mean ± SD (N = three mice).

(H) Representative plot showing sorting gates for CD133^{high} and EGFP$^+$ cells in *Eomes*$^{T2A\text{-}d2EGFP/+}$*; Emx1*$^{Cre/+}$*; Auts2*$^{fl(ex8)/fl(ex8)}$ cortex.

(I) RT-qPCR analysis for *Sox2, Nes, Pax6, Eomes, Neurod1* and *Tbr1* in the cerebral cortex, sorted CD133^{high} cells and sorted EGFP$^+$ cells from *Eomes*$^{T2A\text{-}d2EGFP/+}$ mice at E15.5. Data are presented as mean ± SD (N = four biological replicates); One-way ANOVA with Turkey's post-hoc test or Kruskal–Wallis test.

(J) Representative images of staining with DAPI (blue) and anti-KI67 (green) in sorted CD133^{high} and EGFP$^+$ cells from *Eomes*$^{T2A\text{-}d2EGFP/+}$*; Auts2*$^{fl(ex8)/fl(ex8)}$ cortex at E15.5. The graph shows the percentage of KI67$^+$ cells in sorted CD133^{high} or EGFP$^+$ cells. Data are presented as the mean ± SD (N = three biological replicates).

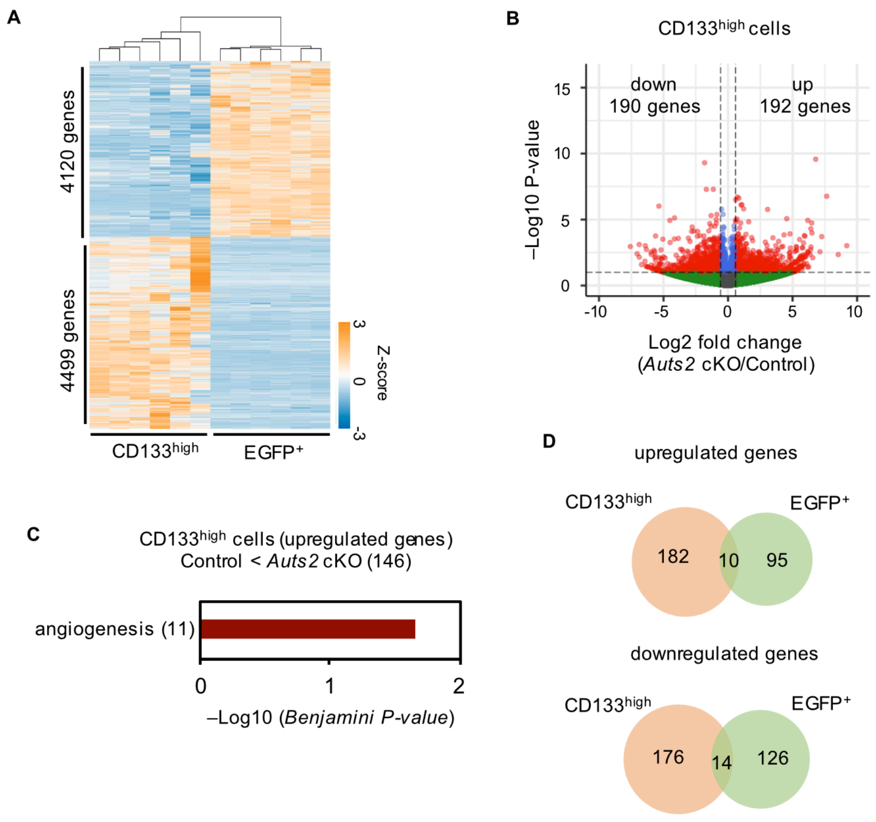

Appendix Fig. S5. Transcriptional profiling of RGCs at E15.5.

(A) Heatmap of differentially expressed genes (DEGs) between CD133^{high} and EGFP^{+} cells from E15.5 control mouse cortices (adjusted P-value < 0.05). In total, 4499 and 4120 genes were enriched in CD133^{high} and EGFP^{+} cells, respectively. The color scale is shown on the bottom right.

(B) Volcano plot showing differences in gene expression in CD133^{high} cells between control and *Auts2* cKO mice. Red plots indicate upregulated and downregulated genes in *Auts2* cKO cells compared with the control (P-value < 0.01 and |Log2 fold-change| > 0.58).

(C) DAVID Gene Ontology biological process analysis of upregulated genes in CD133^{high} cells (Benjamini-Hochberg adjusted P-value < 0.05). Numbers in parentheses show the count of genes.

(D) Venn diagrams showing upregulated or downregulated genes overlap between CD133^{high} and EGFP^{+} cells.

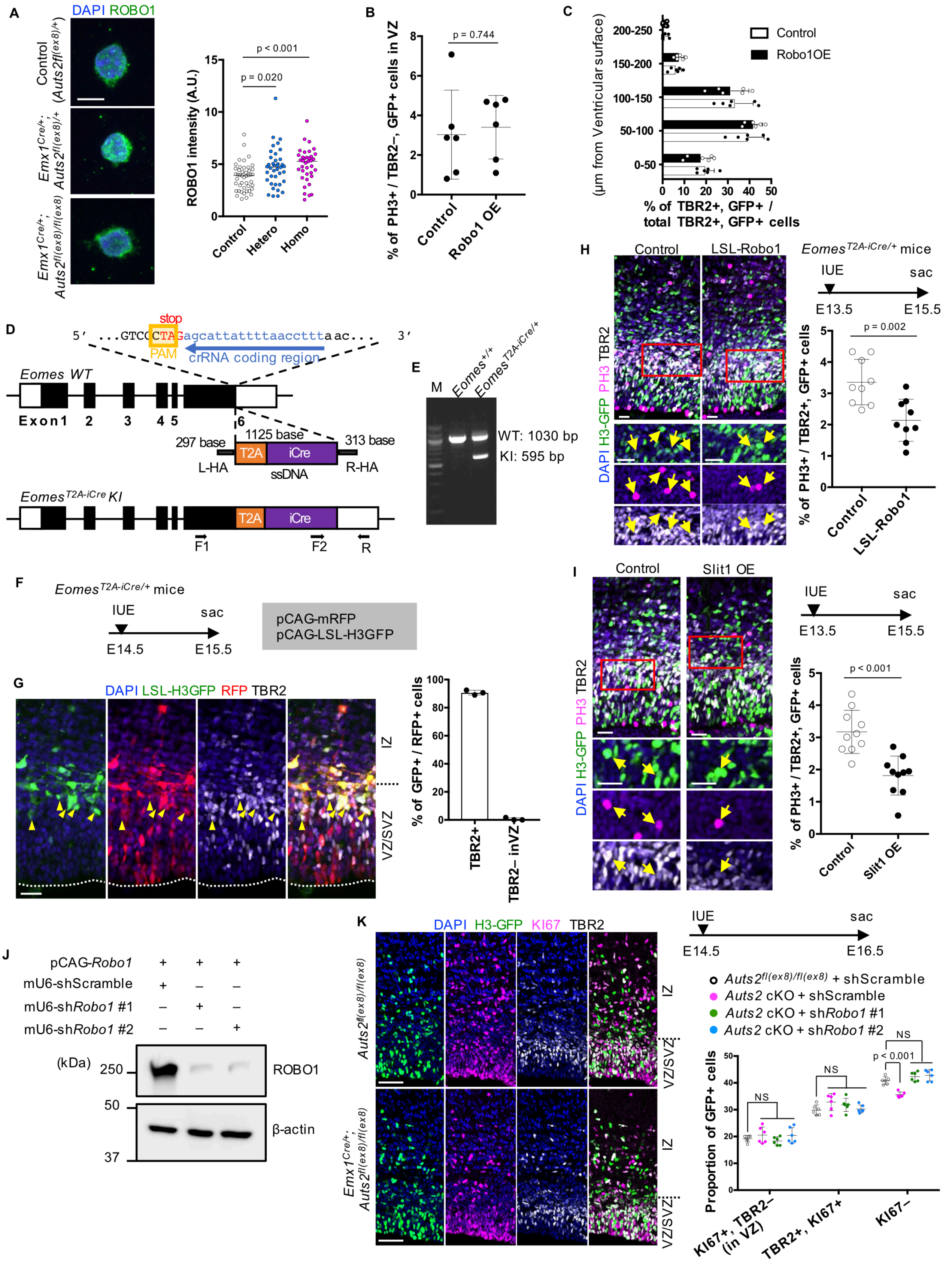

Appendix Fig. S6. *Robo1* overexpression in IPCs reduces the cell division.

(A) Representative z-stack images of DAPI (blue) and ROBO1 (green) staining in sorted EGFP^{+} cells from E15.5 cerebral cortices of indicated genotypes. The graph shows the immunofluorescence intensity of ROBO1. A.U., arbitrary units. Data are

presented as the median, N = three biological replicates, 37–45 cells.

(B) The graph shows the percentage of $PH3^+$ cells in TBR2-negative and GFP^+ cells in the VZ of electroporated animals in Fig. 5A. Data are presented as the mean ± SD (N = three mice, six sections).

(C) The graph shows the distribution of $TBR2^+$ and GFP^+ cells of electroporated animals in Fig. 5A. Bins are set up parallel to the ventricular surface, separated every 50 µm. Data are presented as the mean ± SD (N = three mice, six sections).

(D) Targeting strategy of CRISPR/Cas9 method for the generation of $Eomes^{T2A\text{-}iCre}$ knock-in mouse. The DNA sequence for T2A-iCre is inserted between the last amino acid and the stop codon of the *Eomes* locus. F1 and F2, forward primers for genotyping; R, reverse primer for genotyping.

(E) Genotyping for WT and $Eomes^{T2A\text{-}iCre/+}$ KI mouse using primers indicated in (D).

(F) Experimental design of *in utero* electroporation (IUE) into $Eomes^{T2A\text{-}iCre}$ KI mice.

(G) Representative immunofluorescent images of the electroporated brains (F) for DAPI (blue), H3-GFP (green), RFP (red), and TBR2 (white). Arrowheads indicate GFP^+, RFP^+ and $TBR2^+$ cells. The graph shows the percentage of GFP^+ cells among RFP^+, $TBR2^+$ cells (left), or GFP^+ cells among RFP^+, TBR2-negative cells located in VZ/SVZ (right). Data are presented as the mean ± SD (N = three mice).

(H) IUE of empty (control) or pCAG-LSL-*Robo1* (LSL-*Robo1*) vector together with pCAG-H3GFP into $Eomes^{T2A\text{-}iCre/+}$ KI cortices at E13.5, followed by staining for DAPI (blue), H3-GFP (green), PH3 (magenta) and TBR2 (white) at E15.5. The graph shows the percentage of $PH3^+$ cells in $TBR2^+$ and GFP^+ cells. Arrows indicate the $PH3^+$, $TBR2^+$ and GFP^+ cells. Data are presented as the mean ± SD (N = five mice, nine sections).

(I) IUE of empty (control) or pCAG-*Slit1* (*Slit1* OE) vector plus pCAG-H3GFP into WT cortices at E13.5, followed by staining for DAPI (blue), H3-GFP (green), PH3 (magenta) and TBR2 (white) at E15.5. Arrows indicate the $PH3^+$, $TBR2^+$ and GFP^+ cells. The graph shows the percentage of $PH3^+$ cells in $TBR2^+$ and GFP^+ cells. Data are presented as the mean ± SD (N = six mice, 10 sections).

(J) *In vitro* knockdown (KD) experiment of ROBO1 expression by co-transfection of a *Robo1* expression vector and scrambled shRNA or *Robo1* shRNA vectors into HEK293 cells. The cell lysates were immunoblotted with anti-ROBO1 and anti-β-actin antibodies two days after transfection.

(K) IUE of scrambled or *Robo1* shRNA into $Auts2^{fl(ex8)/fl(ex8)}$ or $Emx1^{Cre/+}$; $Auts2^{fl(ex8)/fl(ex8)}$ (*Auts2* cKO) cortices at E14.5, followed by immunostaining with DAPI (blue), anti-GFP (green), anti-KI67 (magenta) and anti-TBR2 (white) at E16.5. The graph shows the proportion of $KI67^+$ and TBR2-negative cells located to the VZ (RGCs), $TBR2^+$ and $KI67^+$ cells (IPCs), and KI67-negative cells (postmitotic neurons) among total GFP^+ cells. The percentage of RGCs and IPCs in electroporated cells was not different among the indicated samples. Data are presented as the mean ± SD (N = three mice, six sections).

NS, not significant; Kruskal–Wallis test (A), unpaired Student's t-test (B, C, H, I), One-way ANOVA with Dunnett's post-hoc test (K). Scale bars, 5 µm (A), 20 µm (G, H, I) and 50 µm (K).

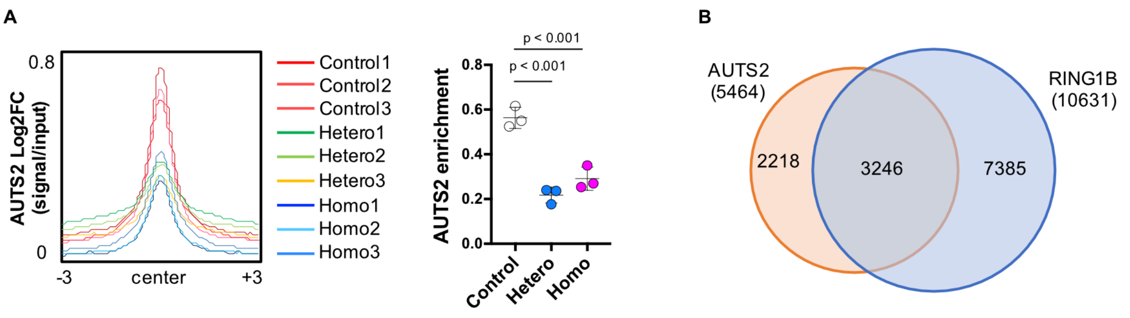

Appendix Fig. S7. CUT&Tag signals of AUTS2 reduced in *Auts2* mutants.

(A) Density plots of AUTS2 CUT&Tag signals in control, *Auts2* cKO heterozygous and homozygous cells centered on AUTS2-binding loci (±3 kb). The graph shows the enrichment of CUT&Tag signals for AUTS2 in control and *Auts2* mutants. FC, fold change. N = three biological replicates. Data are presented as the mean ± SD; One-way ANOVA with Dunnett's post-hoc test.

(B) Venn diagrams comparing the AUTS2-binding loci with RING1B-binding loci in control cells.

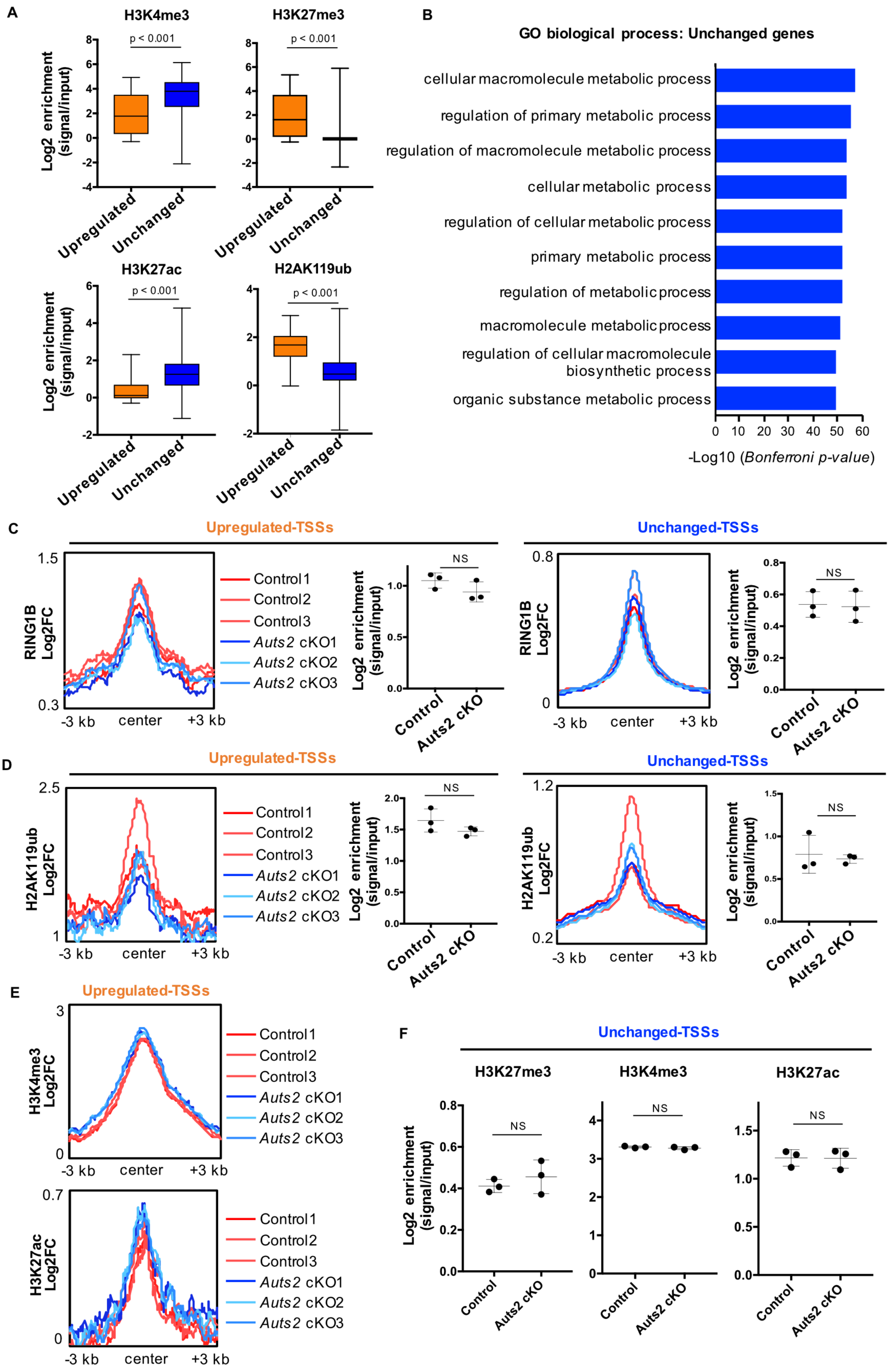

Appendix Fig. S8. Loss of *Auts2* alters histone modifications in IPC-upregulated-TSSs but not in IPC-unchanged-TSSs.

(A) Enrichment of H3K4me3, H3K27me3, H3K27ac and H2AK119ub CUT&Tag signals on IPC-upregulated-TSSs (61 loci) and IPC-unchanged-TSSs (2717 loci). Mann–Whitney U-test. These histone modifications were also significantly different for

the other two biological replicates.

(B) Graph showing the top 10 GO-term biological processes for genes with IPC-unchanged-TSSs on GREAT.

(C) Density plots of RING1B CUT&Tag signals centered on IPC-upregulated-TSSs (left) and IPC-unchanged-TSSs (right) (±3 kb) in control and *Auts2* cKO cells. The graph shows the enrichment of the signals on indicated loci.

(D) Density plots of H2AK119ub CUT&Tag signals centered on IPC-upregulated-TSSs (left) and IPC-unchanged-TSSs (right) (±3 kb) in control and *Auts2* cKO cells. The graph shows the enrichment of the signals on indicated loci.

(E) Density plots of H3K4me3 (top) and H3K27ac (bottom) CUT&Tag signals centered on IPC-upregulated-TSSs (±3 kb) in control and *Auts2* cKO cells.

(F) Graphs showing the enrichment of H3K27me3, H3K4me3, and H3K27ac in IPC-unchanged TSSs in control and *Auts2* cKO cells.

Data are presented as the mean ± SD (N = three biological replicates). NS, not significant; unpaired Student's t-test (C, D, F).

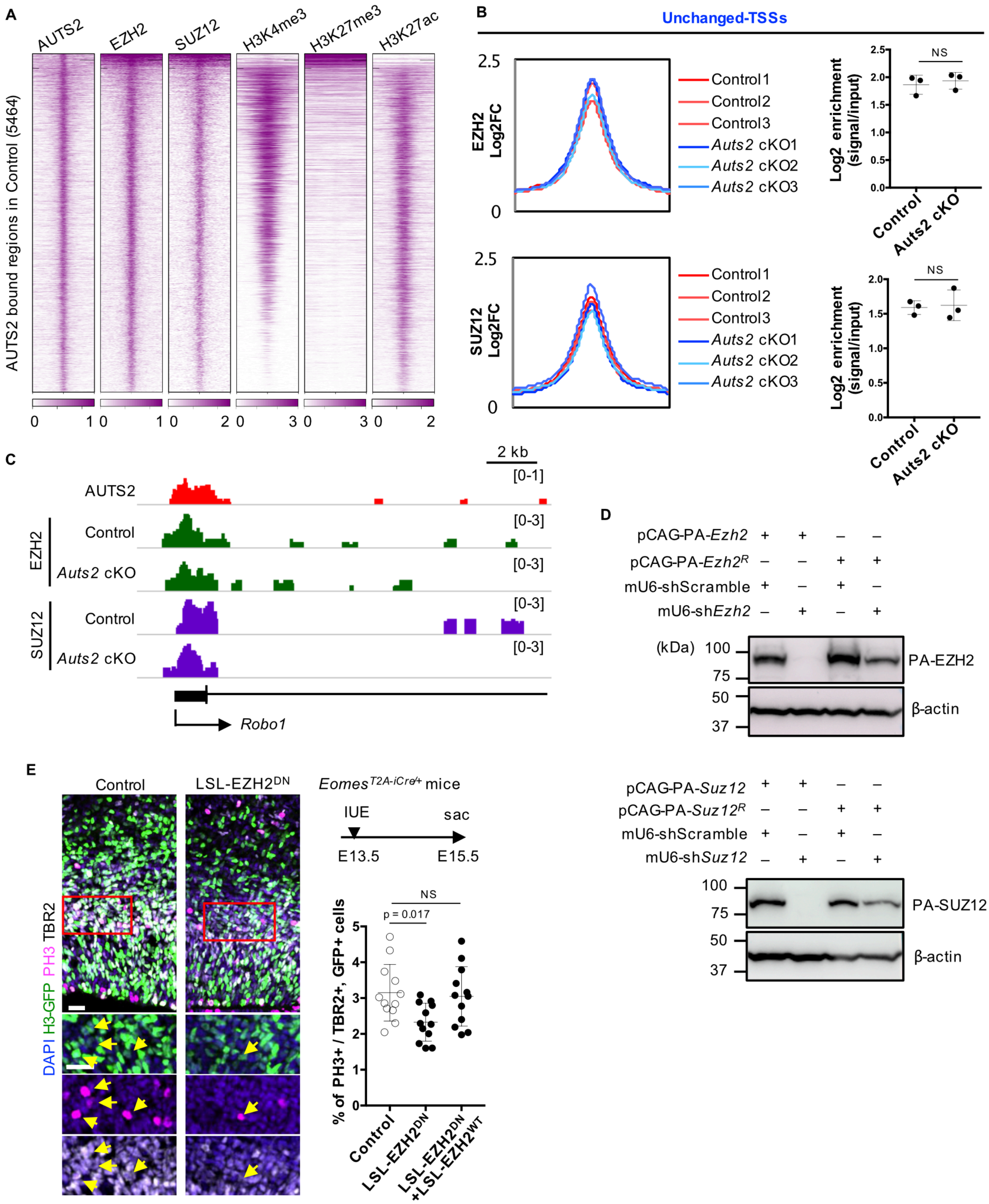

Appendix Fig. S9: Genome wide distribution of PRC2 proteins in IPCs and effect of PRC2 deficiency on IPC division.

(A) Heatmap of AUTS2, EZH2, SUZ12, H3K4me4, H3K27me3 and H3K27ac CUT&Tag signals centered on AUTS2-binding loci (±3 kb) identified in control cells at E15.5.

(B) Density plots of EZH2 (top) and SUZ12 (bottom) signals centered on IPC-upregulated-TSSs (±3 kb) in control and *Auts2* cKO cells. Graphs show the enrichment of EZH2 and SUZ12 on IPC-upregulated-TSSs. Data are presented as the means ± SD (N = three biological replicates). NS, not significant, unpaired Student's t-test.

(C) IGV browser views showing the CUT&Tag signal for AUTS2 in control, EZH2 and SUZ12 in control and *Auts2* cKO cells around the TSS of *Robo1* locus.

(D) *In vitro* KD experiments for EZH2 (top) and SUZ12 (bottom) by co-transfection of PA-tagged expression vectors together with scrambled shRNA or indicated shRNA vectors into HEK293T cells. Two days after transfection, the cell lysates were immunoblotted with anti-PA and anti-β-actin antibodies. pCAG-PA-$Ezh2^R$ and pCAG-PA-$Suz12^R$ indicate the shRNA-resistant expression vectors. The KD effect was much lower against each resistant mRNA.

(E) IUE of indicated vector with pCAG-H3GFP into $Eomes^{T2A\text{-}iCre/+}$ KI cortices at E13.5, followed by staining for DAPI (blue), H3-GFP (green), PH3 (magenta) and TBR2 (white) at E15.5. Arrows indicate the $PH3^+$, $TBR2^+$ and GFP^+ cells. The graph shows the percentage of $PH3^+$ cells in $TBR2^+$, GFP^+ cells. Data are presented as the mean ± SD (N = six mice, 12 sections), One-way ANOVA with Dunnett's post-hoc test.

Section S1: Sequences of the inserted T2A and d2EGFP.

5′-
ggcagtggagagggcagaggaagtctgctaacatgcggtgacgtcgaggagaatcctggcccaATGGTGAGCAAGGGCGAGGA
GCTGTTCACCGGGGTGGTGCCCATCCTGGTCGAGCTGGACGGCGACGTAAACGGCCACAAGT
TCAGCGTGTCCGGCGAGGGCGAGGGCGATGCCACCTACGGCAAGCTGACCCTGAAGTTCATC
TGCACCACCGGCAAGCTGCCCGTGCCCTGGCCCACCCTCGTGACCACCCTGACCTACGGCGT
GCAGTGCTTCAGCCGCTACCCCGACCACATGAAGCAGCACGACTTCTTCAAGTCCGCCATGC
CCGAAGGCTACGTCCAGGAGCGCACCATCTTCTTCAAGGACGACGGCAACTACAAGACCCGC
GCCGAGGTGAAGTTCGAGGGCGACACCCTGGTGAACCGCATCGAGCTGAAGGGCATCGACT
TCAAGGAGGACGGCAACATCCTGGGGCACAAGCTGGAGTACAACTACAACAGCCACAACGT
CTATATCATGGCCGACAAGCAGAAGAACGGCATCAAGGTGAACTTCAAGATCCGCCACAACA
TCGAGGACGGCAGCGTGCAGCTCGCCGACCACTACCAGCAGAACACCCCCATCGGCGACGG
CCCCGTGCTGCTGCCCGACAACCACTACCTGAGCACCCAGTCCGCCCTGAGCAAAGACCCCA
ACGAGAAGCGCGATCACATGGTCCTGCTGGAGTTCGTGACCGCCGCCGGGATCACTCTCGGC
ATGGACGAGCTGTACAAGAAGCTTAGCCATGGCTTCCCGCCGGAGGTGGAGGAGCAGGATGA
TGGCACGCTGCCCATGTCTTGTGCCCAGGAGAGCGGGATGGACCGTCACCCTGCAGCCTGTG
CTTCTGCTAGGATCAATGTGTAG -3′

Section S2: Sequences of the inserted T2A, iCre and BamHI.

5′-
ggcagtggagagggcagaggaagtctgctaacatgcggtgacgtcgaggagaatcctggcccaATGGTGCCCAAGAAGAAGAG
GAAAGTCTCCAACCTGCTGACTGTGCACCAAAACCTGCCTGCCCTCCCTGTGGATGCCACCT
CTGATGAAGTCAGGAAGAACCTGATGGACATGTTCAGGGACAGGCAGGCCTTCTCTGAACAC
ACCTGGAAGATGCTCCTGTCTGTGTGCAGATCCTGGGCTGCCTGGTGCAAGCTGAACAACAG
GAAATGGTTCCCTGCTGAACCTGAGGATGTGAGGGACTACCTCCTGTACCTGCAAGCCAGAG
GCCTGGCTGTGAAGACCATCCAACAGCACCTGGGCCAGCTCAACATGCTGCACAGGAGATCT
GGCCTGCCTCGCCCTTCTGACTCCAATGCTGTGTCCCTGGTGATGAGGAGAATCAGAAAGGA
GAATGTGGATGCTGGGGAGAGAGCCAAGCAGGCCCTGGCCTTTGAACGCACTGACTTTGACC
AAGTCAGATCCCTGATGGAGAACTCTGACAGATGCCAGGACATCAGGAACCTGGCCTTCCTG
GGCATTGCCTACAACACCCTGCTGCGCATTGCCGAAATTGCCAGAATCAGAGTGAAGGACAT
CTCCCGCACCGATGGTGGGAGAATGCTGATCCACATTGGCAGGACCAAGACCCTGGTGTCCA
CAGCTGGTGTGGAGAAGGCCCTGTCCCTGGGGGTTACCAAGCTGGTGGAGAGATGGATCTCT
GTGTCTGGTGTGGCTGATGACCCCAACAACTACCTGTTCTGCCGGGTCAGAAAGAATGGTGT
GGCTGCCCCTTCTGCCACCTCCCAACTGTCCACCCGGGCCCTGGAAGGGATCTTTGAGGCCA
CCCACCGCCTGATCTATGGTGCCAAGGATGACTCTGGGCAGAGATACCTGGCCTGGTCTGGCC
ACTCTGCCAGAGTGGGTGCTGCCAGGGACATGGCCAGGGCTGGTGTGTCCATCCCTGAAATC
ATGCAGGCTGGTGGCTGGACCAATGTGAACATTGTGATGAACTACATCAGAAACCTGGACTCT
GAGACTGGGGCCATGGTGAGGCTGCTCGAGGATGGGGACTGAGGATCC -3′
