# Supplementary figures and images for: The microcephaly-associated transcriptional regulator AUTS2 cooperates with Polycomb complex PRC2 to produce upper-layer neurons in mice (part 1 of 2)
Source: EMBO J. 2025 Jan 15;44(5):1354–78. doi: 10.1038/s44318-024-00343-7 (PMC11876313; doi:10.1038/s44318-024-00343-7)

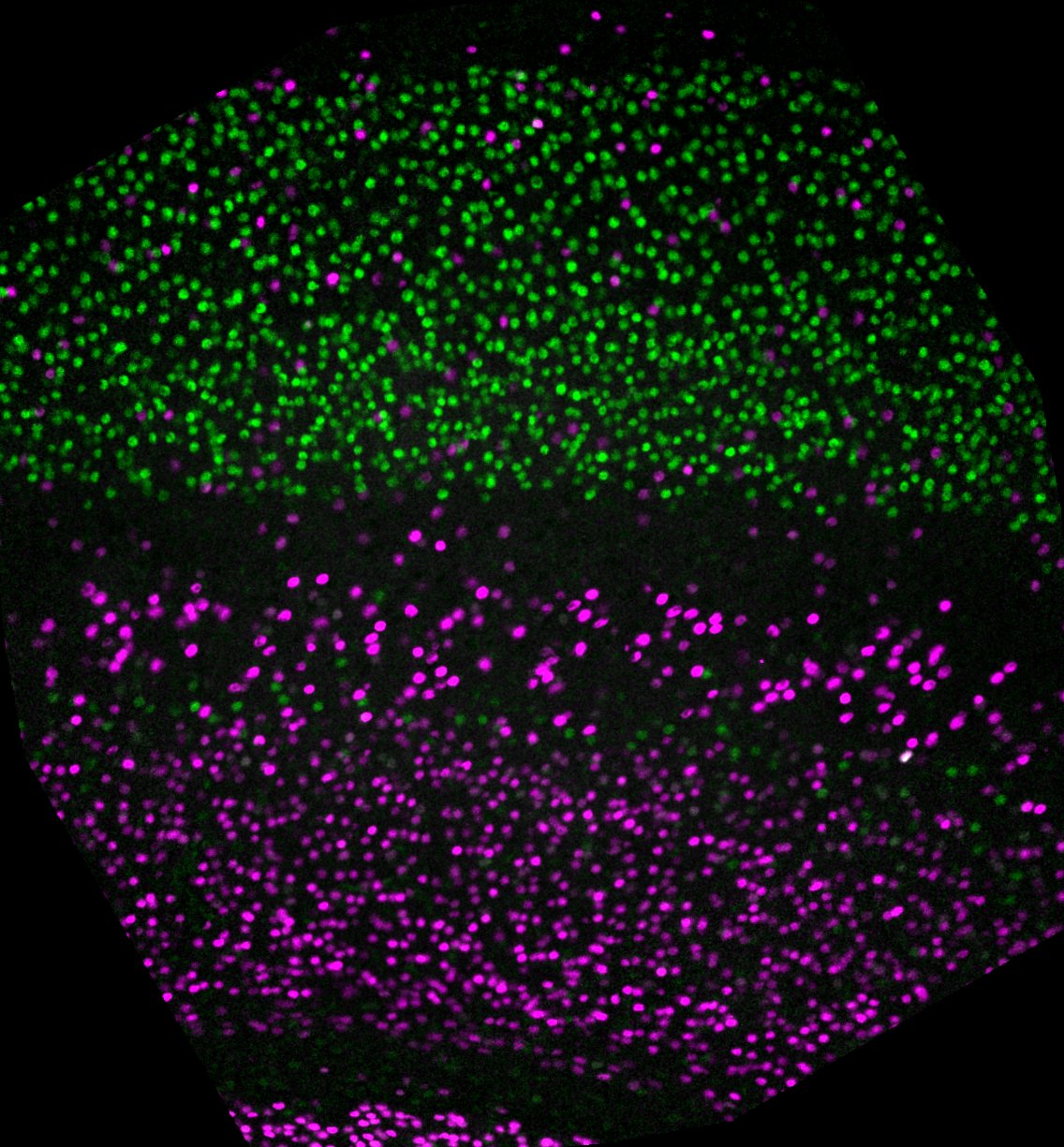

Supplement: Supplementary file 6 — Source data Fig. 1 [file 44318_2024_343_MOESM6_ESM.zip › Figure1/1F/Auts2fl:fl_CUX1,CTIP2.jpg]

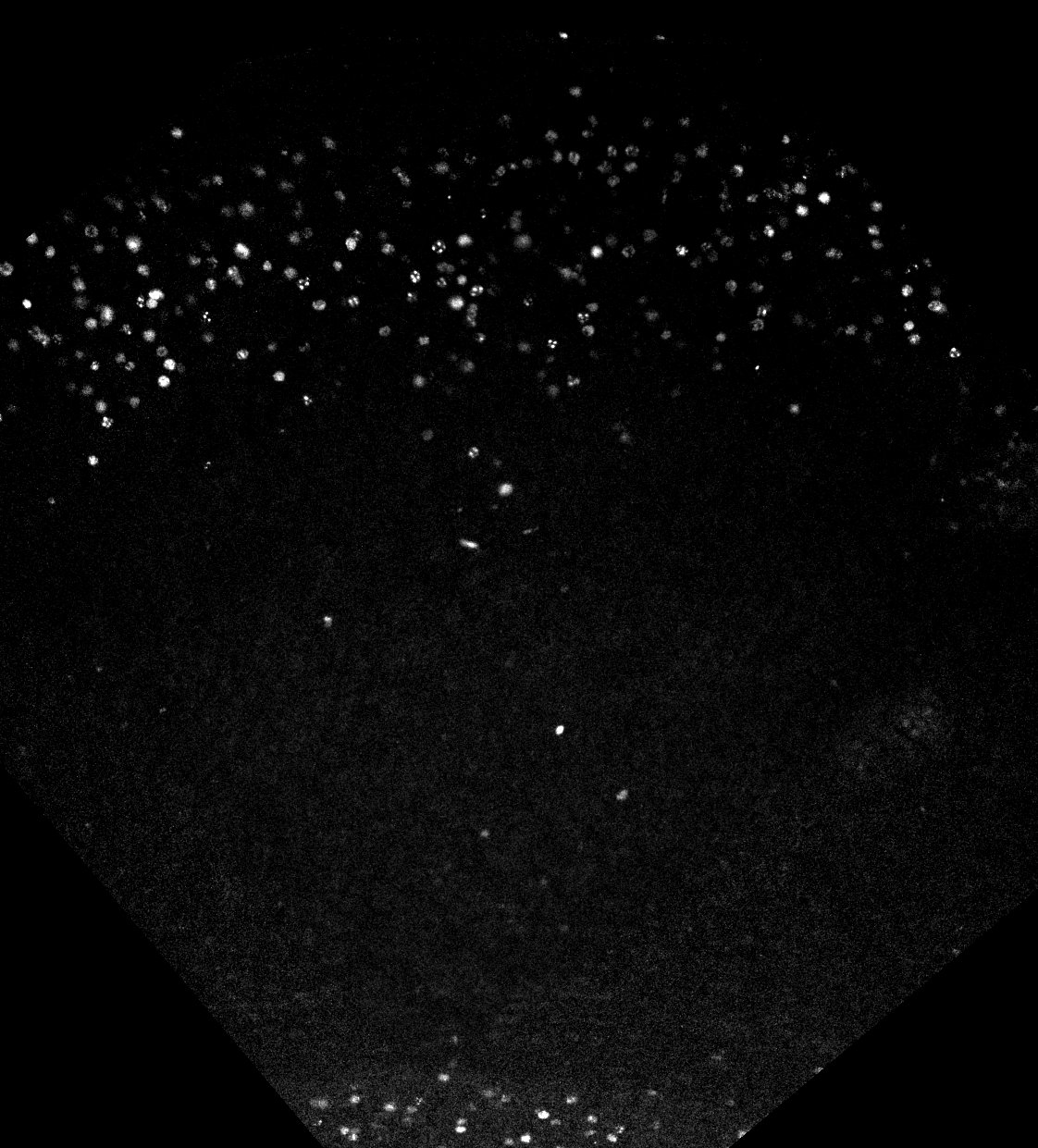

Supplement: Supplementary file 6 — Source data Fig. 1 [file 44318_2024_343_MOESM6_ESM.zip › Figure1/1F/Emx1Cre:+;Auts2fl:fl_EdU.jpg]

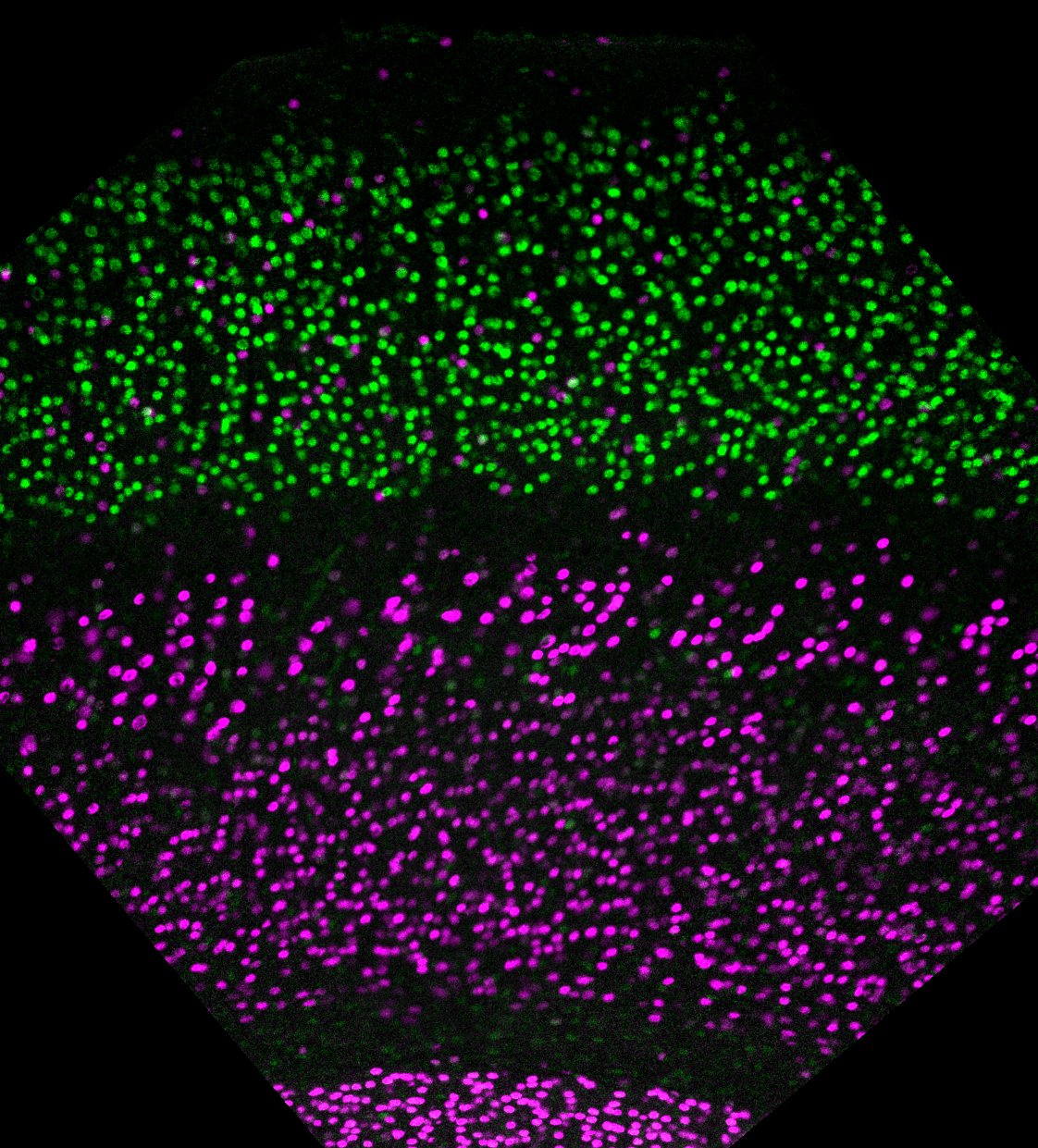

Supplement: Supplementary file 6 — Source data Fig. 1 [file 44318_2024_343_MOESM6_ESM.zip › Figure1/1F/Emx1Cre:+;Auts2flfl_CUX1,CTIP2.jpg]

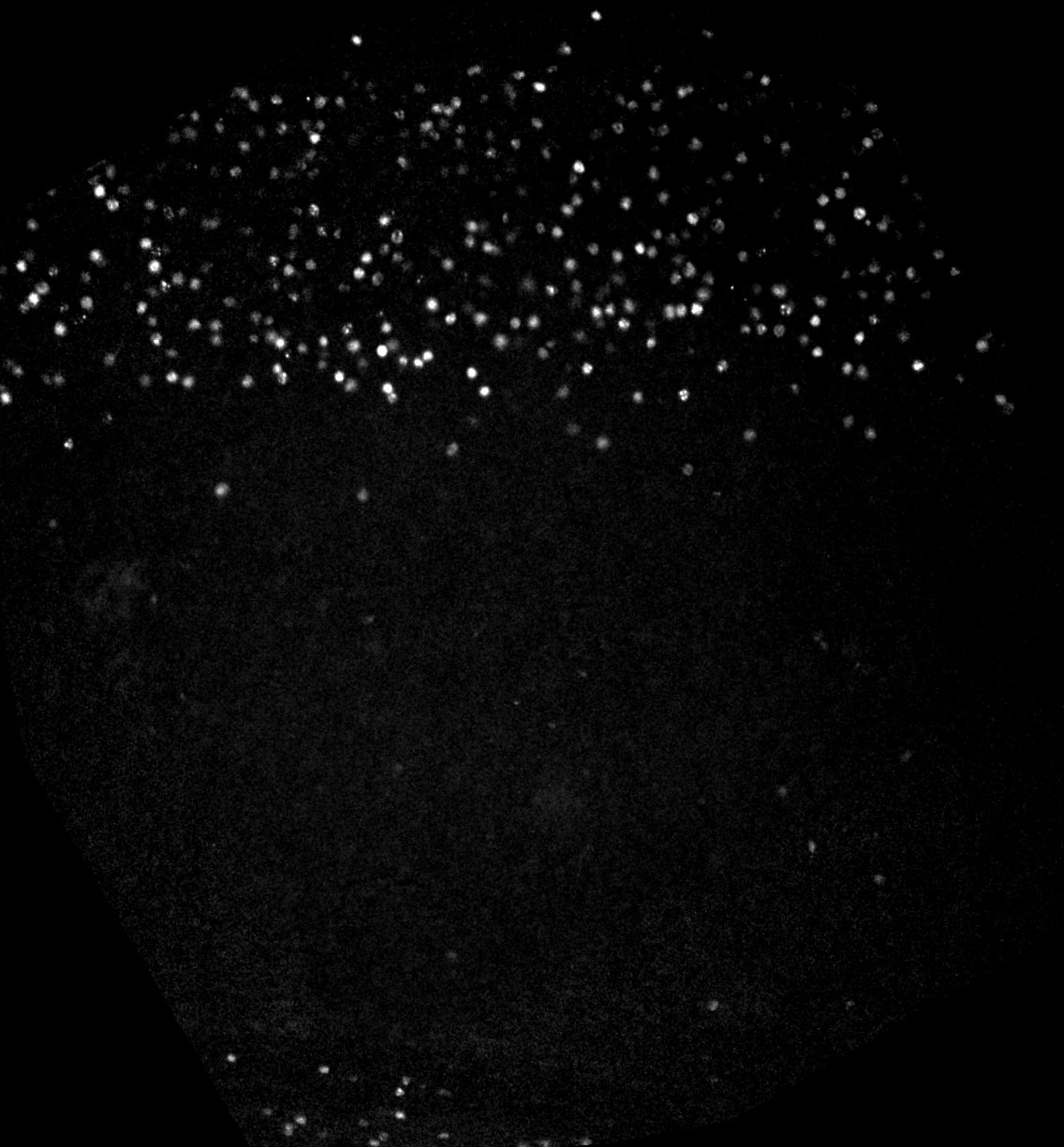

Supplement: Supplementary file 6 — Source data Fig. 1 [file 44318_2024_343_MOESM6_ESM.zip › Figure1/1F/Auts2fl::fl_EdU.jpg]

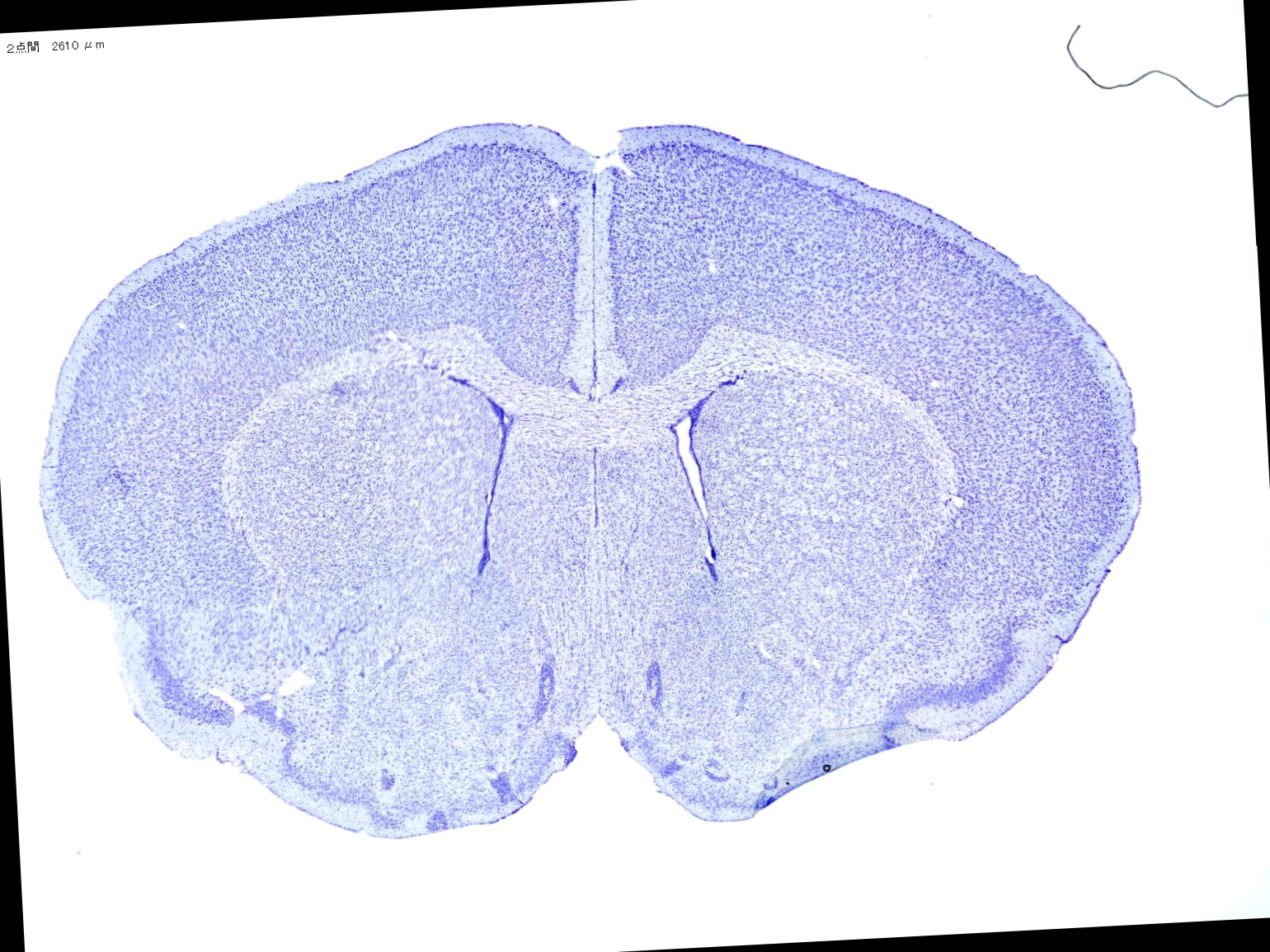

Supplement: Supplementary file 6 — Source data Fig. 1 [file 44318_2024_343_MOESM6_ESM.zip › Figure1/1A/Emx1-Cre:+;Auts2fl:fl.jpg]

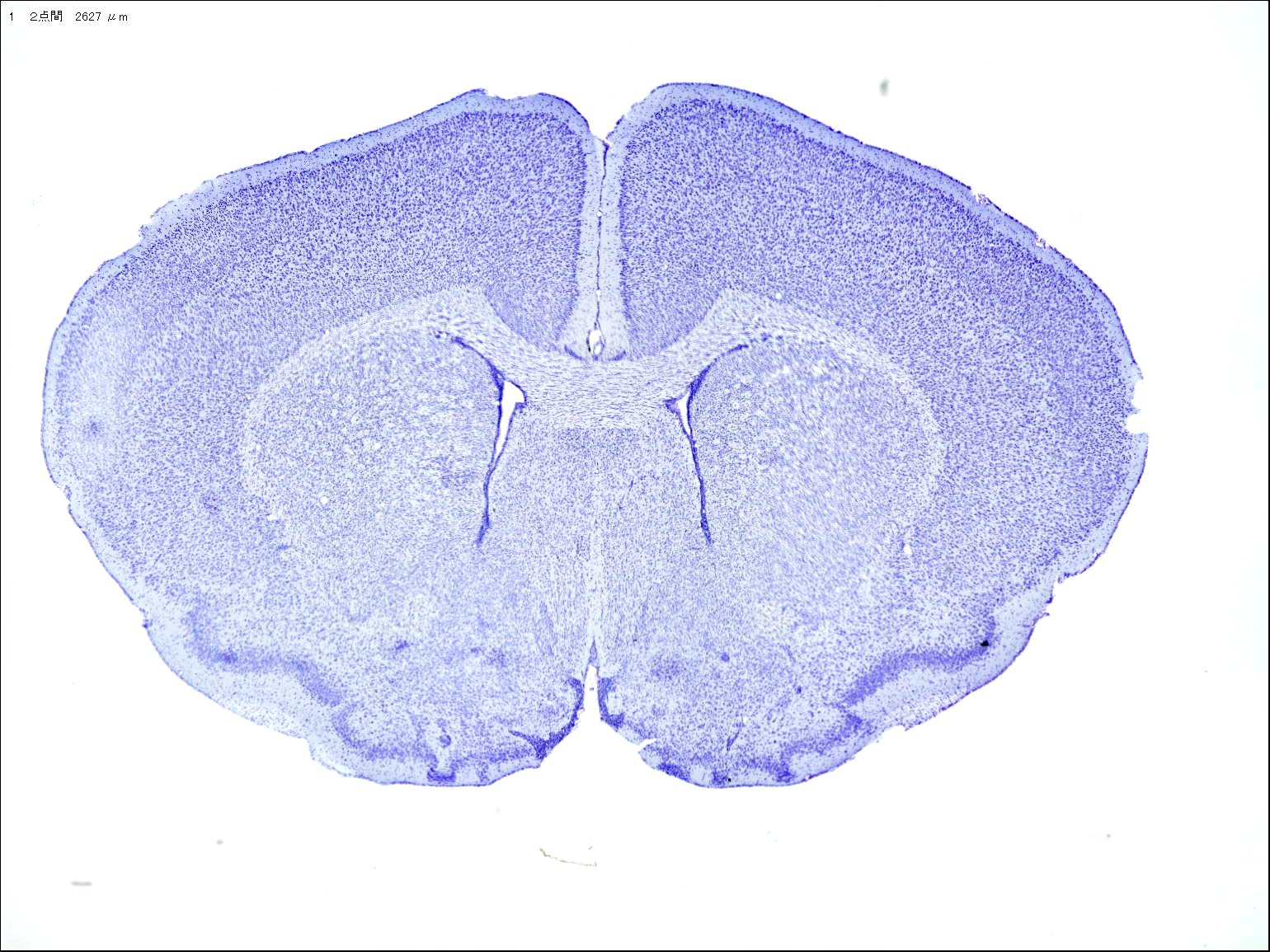

Supplement: Supplementary file 6 — Source data Fig. 1 [file 44318_2024_343_MOESM6_ESM.zip › Figure1/1A/Auts2fl:fl.jpg]

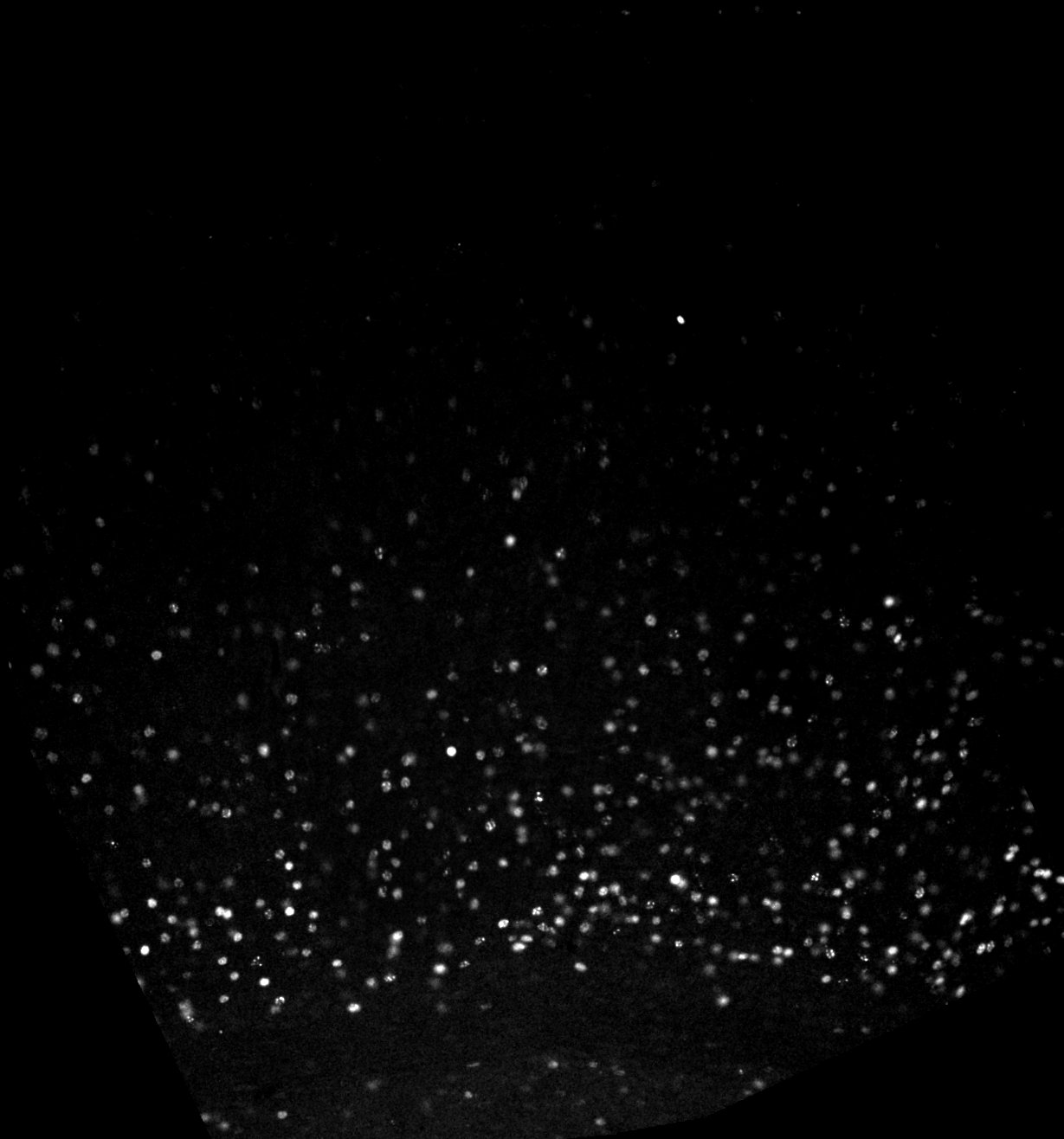

Supplement: Supplementary file 6 — Source data Fig. 1 [file 44318_2024_343_MOESM6_ESM.zip › Figure1/1G/Auts2fl:fl_EdU.jpg]

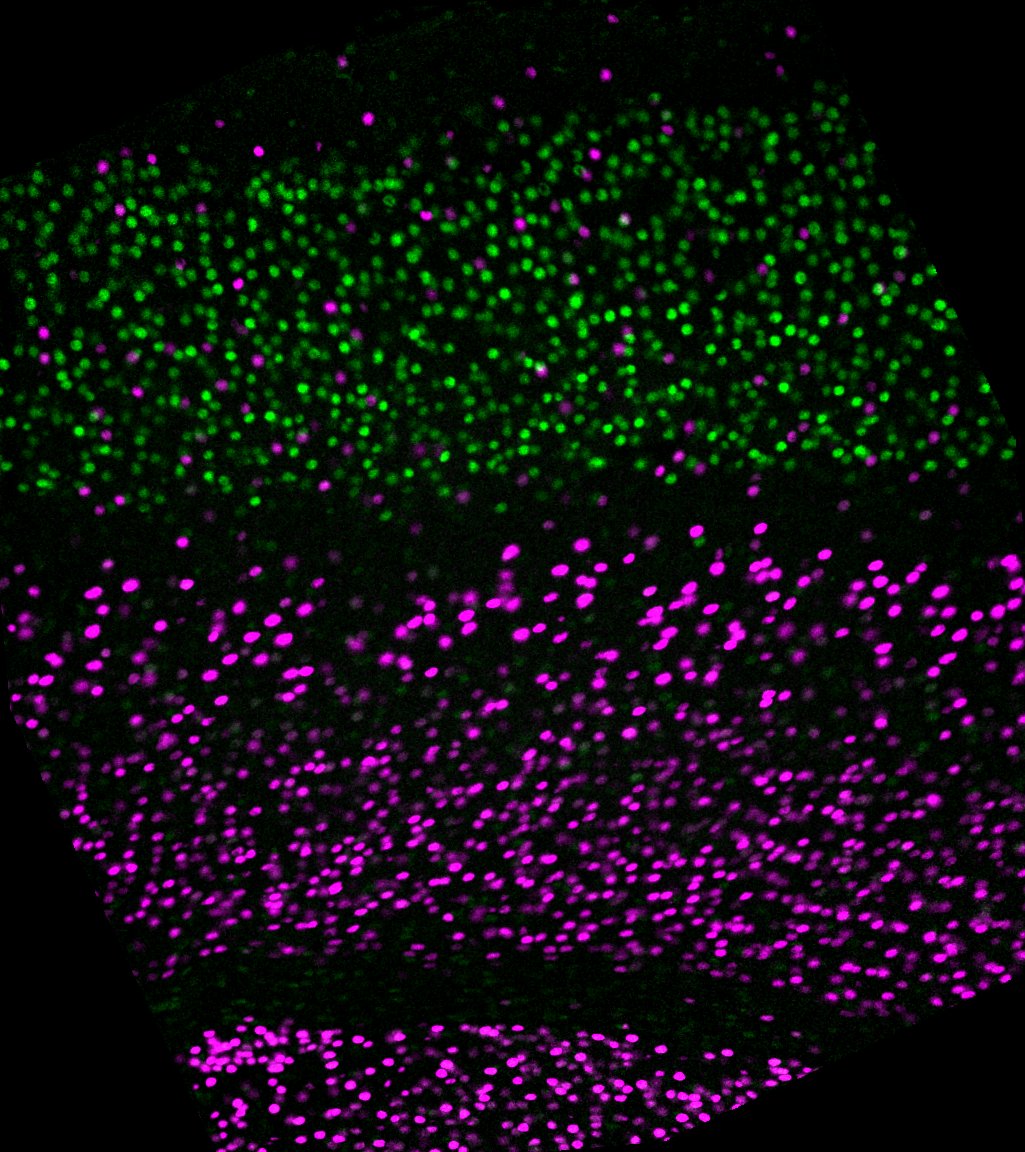

Supplement: Supplementary file 6 — Source data Fig. 1 [file 44318_2024_343_MOESM6_ESM.zip › Figure1/1G/Emx1-Cre:+;Auts2fl:fl_CUX1,CTIP2.jpg]

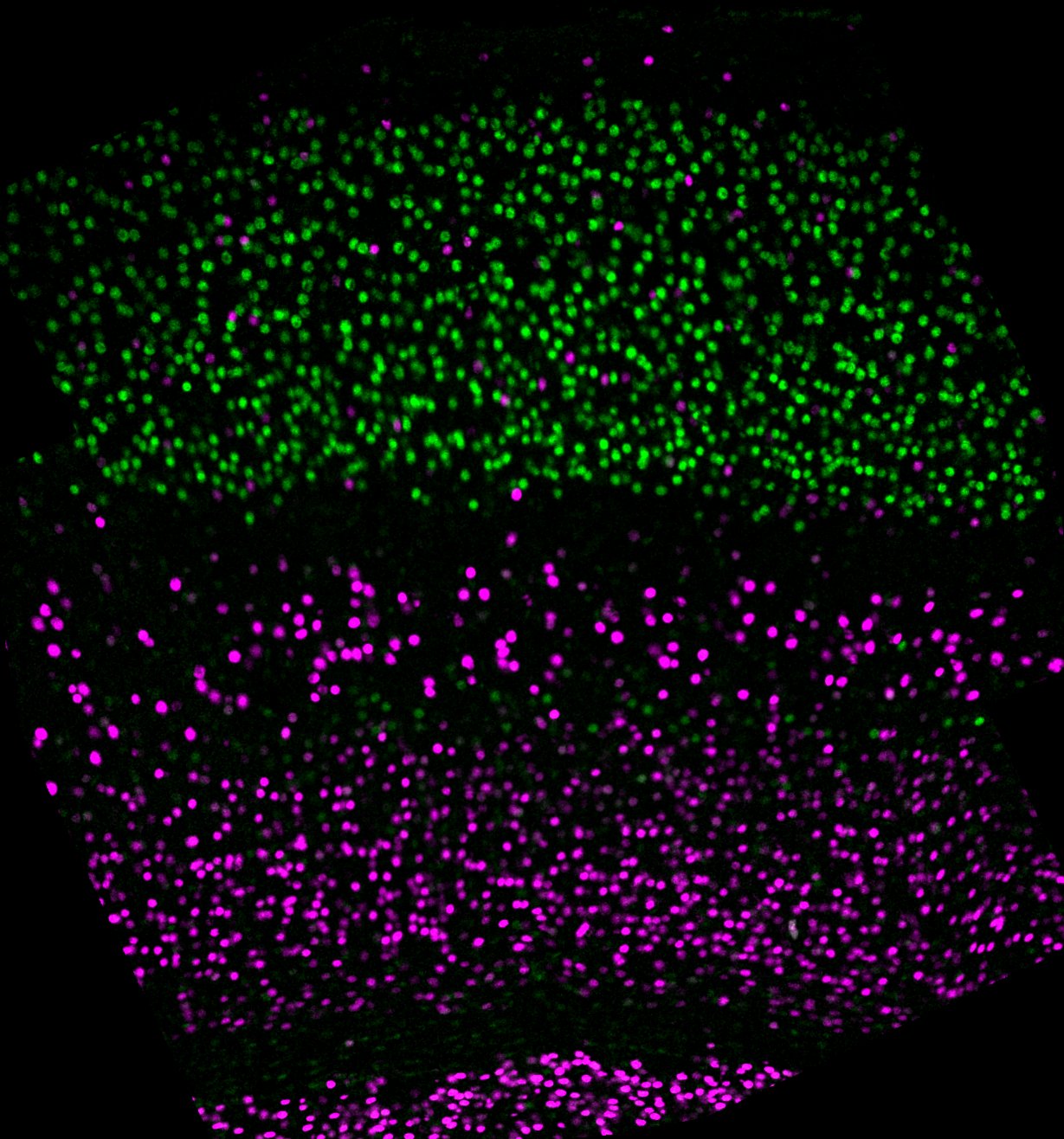

Supplement: Supplementary file 6 — Source data Fig. 1 [file 44318_2024_343_MOESM6_ESM.zip › Figure1/1G/Auts2fl:fl_CUX1,CTIP2.jpg]

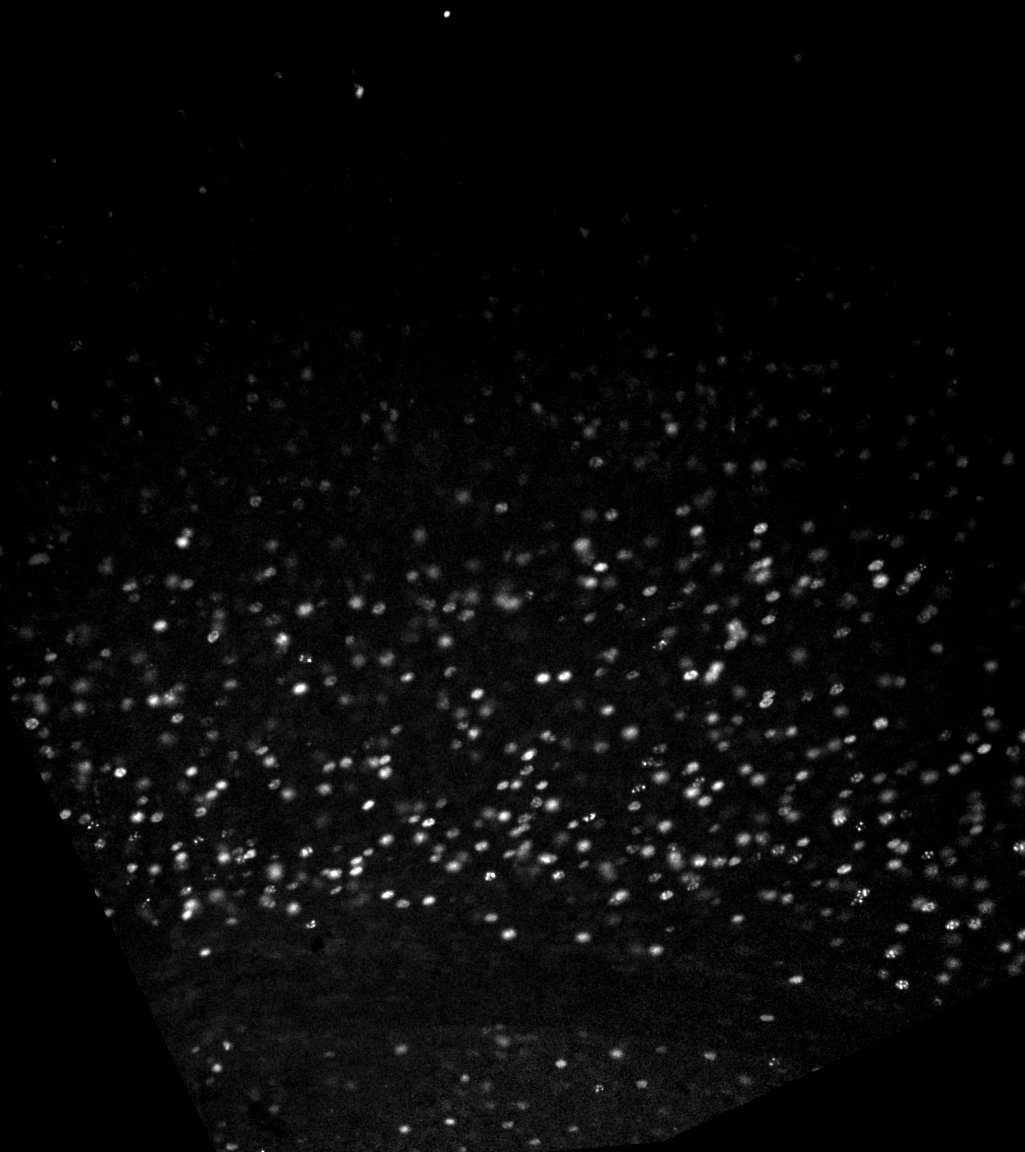

Supplement: Supplementary file 6 — Source data Fig. 1 [file 44318_2024_343_MOESM6_ESM.zip › Figure1/1G/Emx1Cre:+;Auts2fl:fl_EdU.jpg]

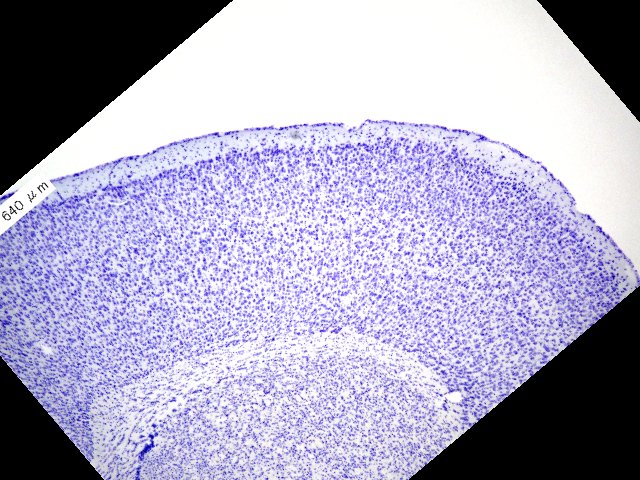

Supplement: Supplementary file 6 — Source data Fig. 1 [file 44318_2024_343_MOESM6_ESM.zip › Figure1/1B/Emx1-cre:+;Auts2fl:fl.jpg]

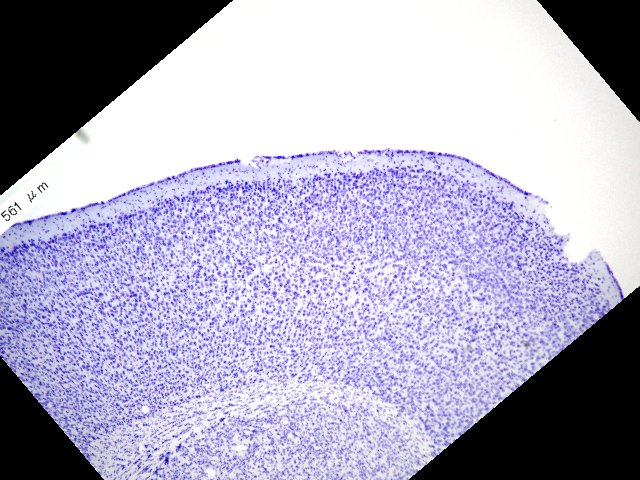

Supplement: Supplementary file 6 — Source data Fig. 1 [file 44318_2024_343_MOESM6_ESM.zip › Figure1/1B/Auts2fl:fl.jpg]

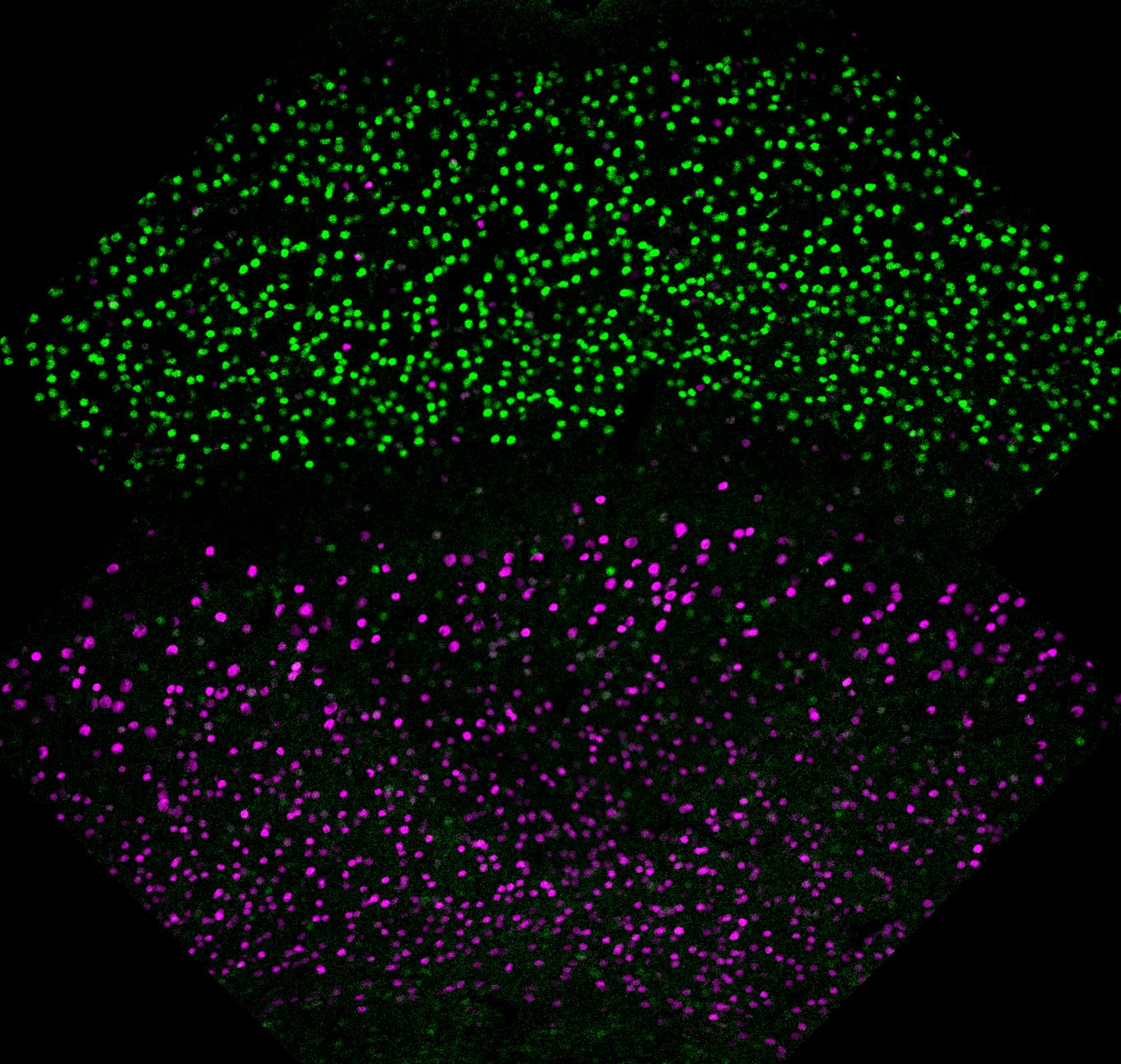

Supplement: Supplementary file 6 — Source data Fig. 1 [file 44318_2024_343_MOESM6_ESM.zip › Figure1/1C/Emx1Cre:+;Auts2fl:fl_PD15.jpg]

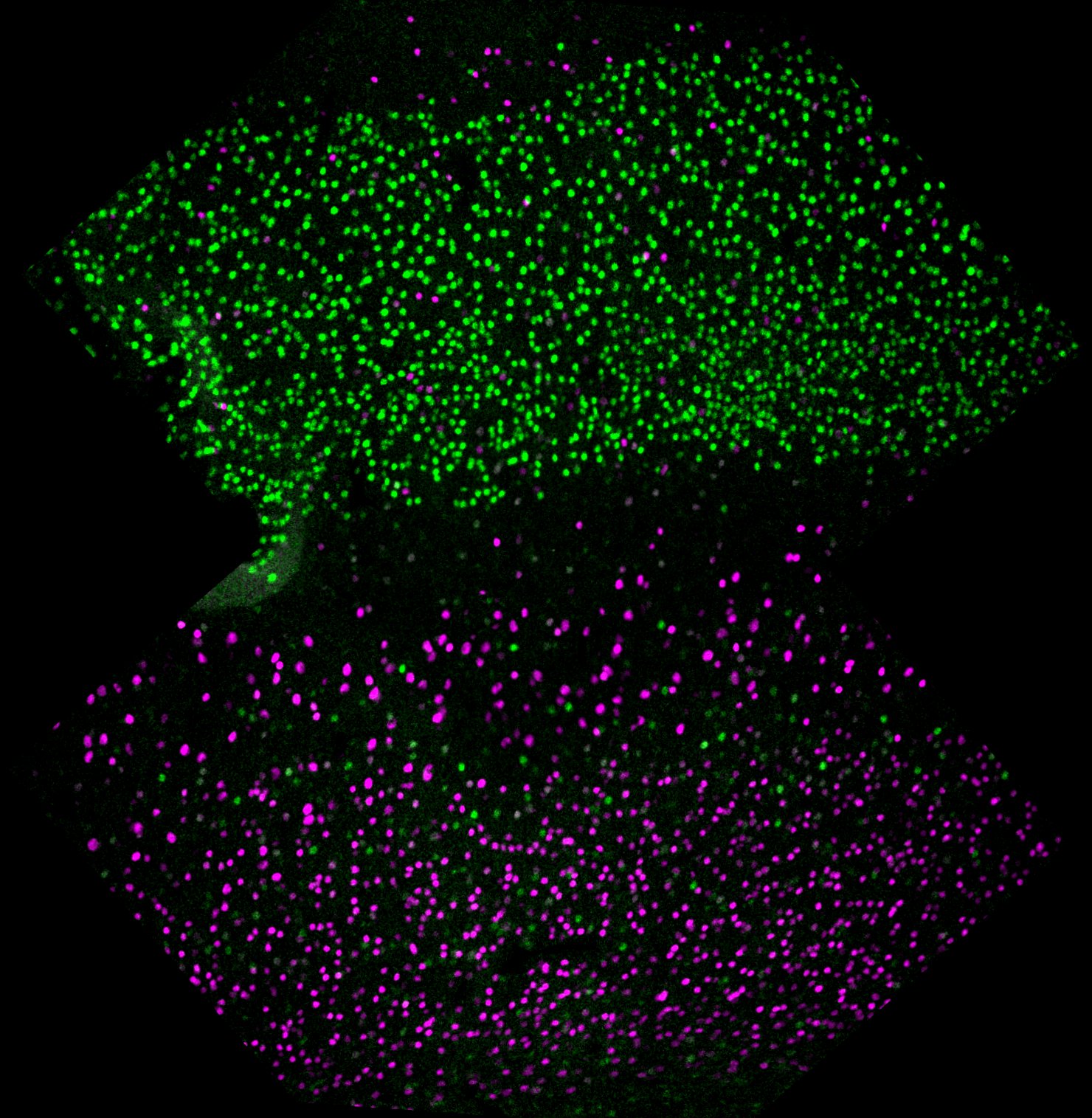

Supplement: Supplementary file 6 — Source data Fig. 1 [file 44318_2024_343_MOESM6_ESM.zip › Figure1/1C/Auts2fl:fl_PD15.jpg]

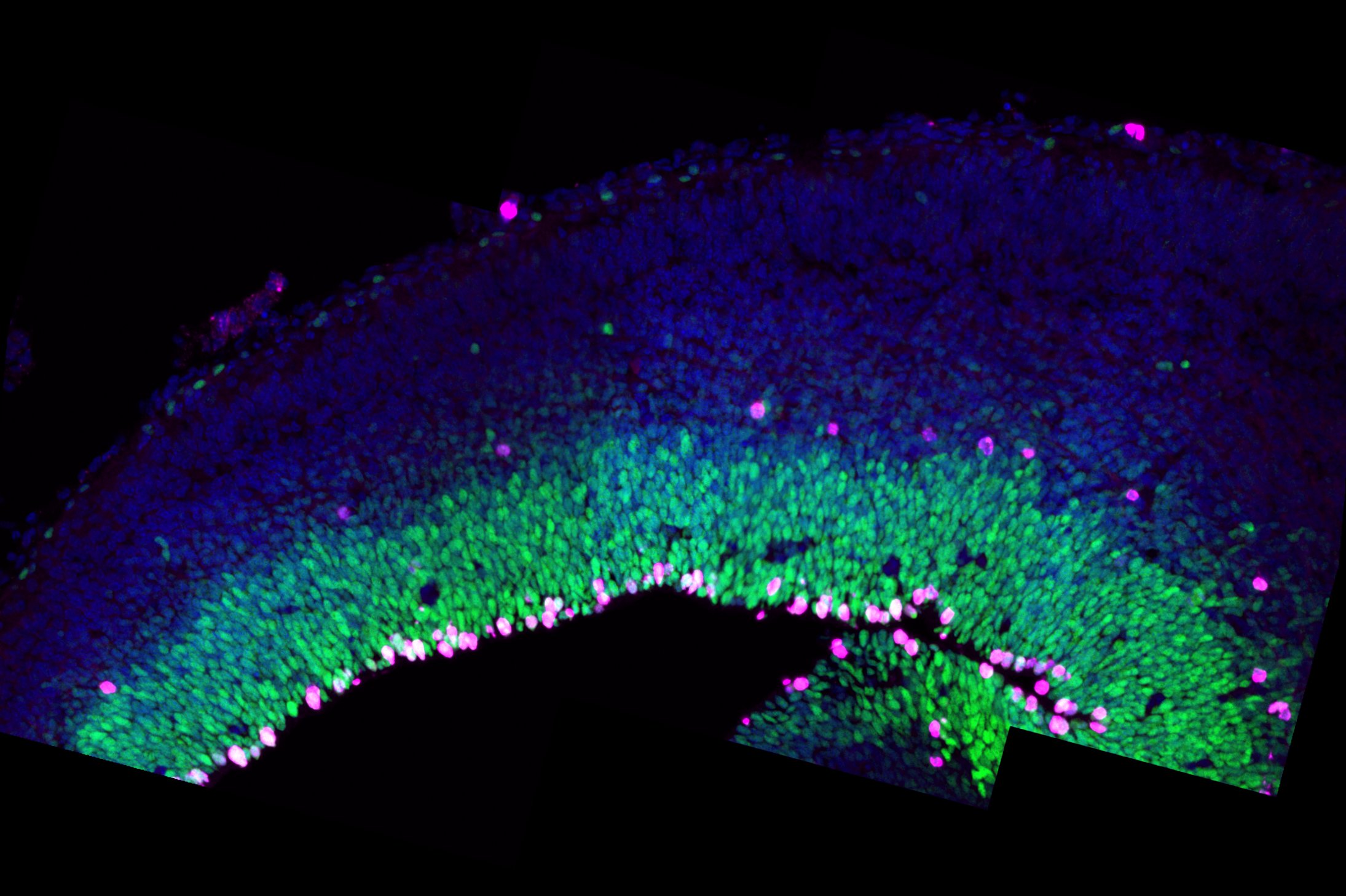

Supplement: Supplementary file 7 — Source data Fig. 2 [file 44318_2024_343_MOESM7_ESM.zip › Figure2/2A/Homo.jpg]

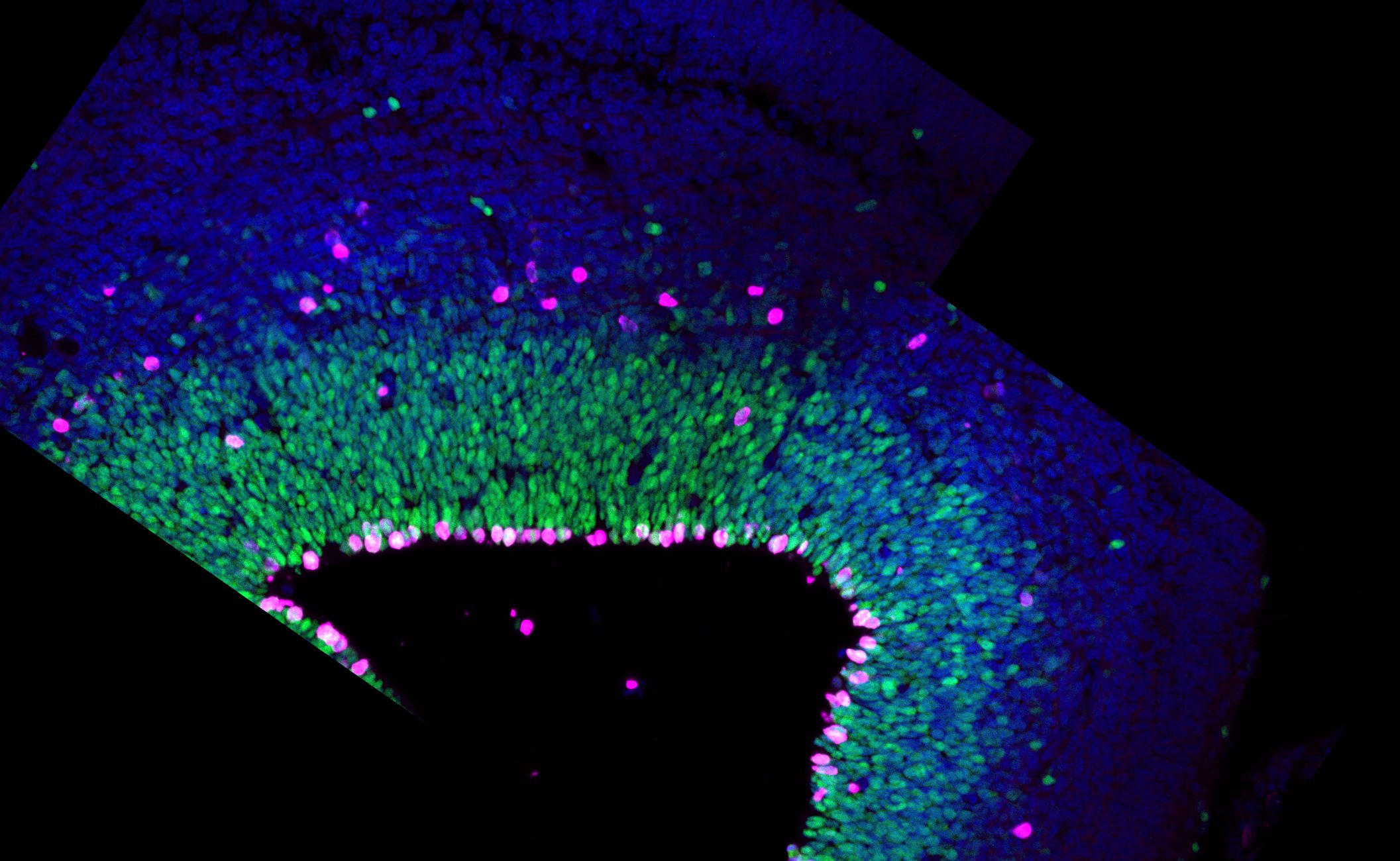

Supplement: Supplementary file 7 — Source data Fig. 2 [file 44318_2024_343_MOESM7_ESM.zip › Figure2/2A/WT.jpg]

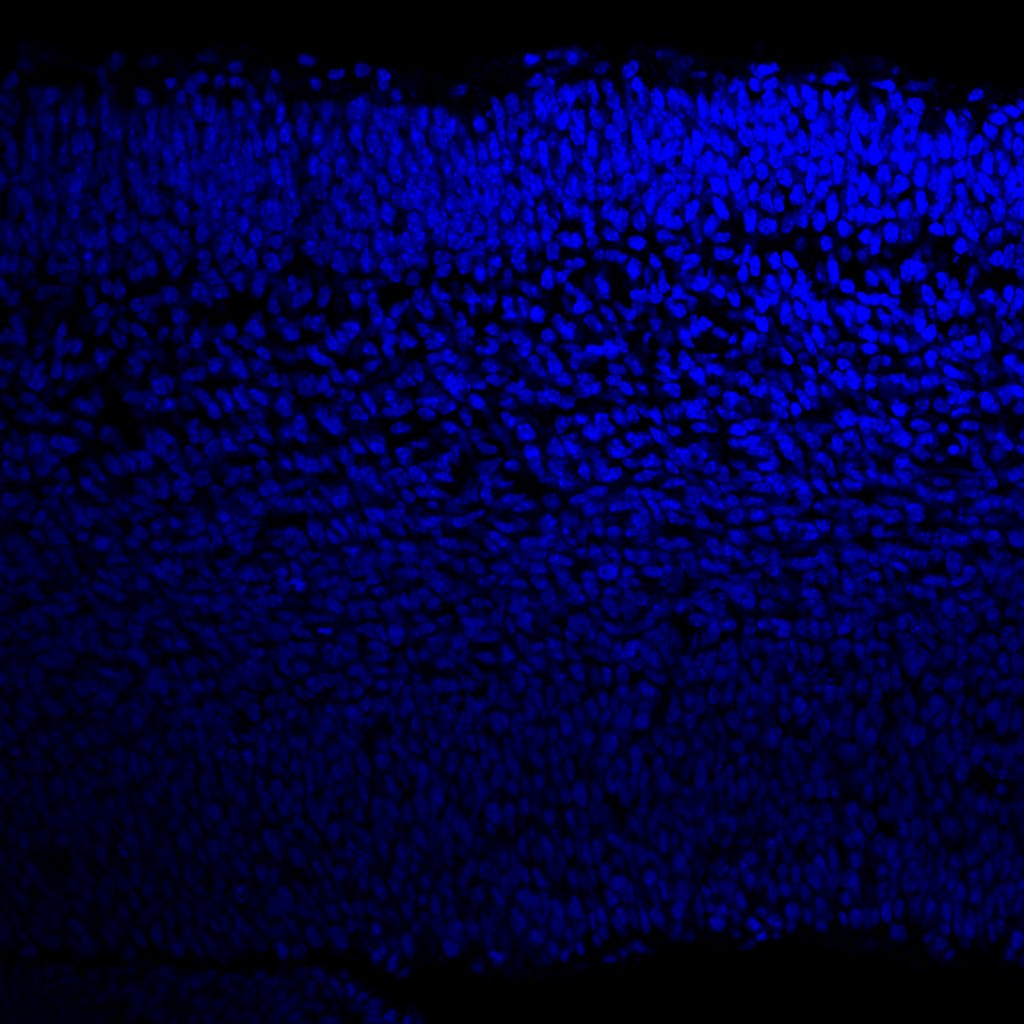

Supplement: Supplementary file 7 — Source data Fig. 2 [file 44318_2024_343_MOESM7_ESM.zip › Figure2/2C/WT_DAPI.jpg]

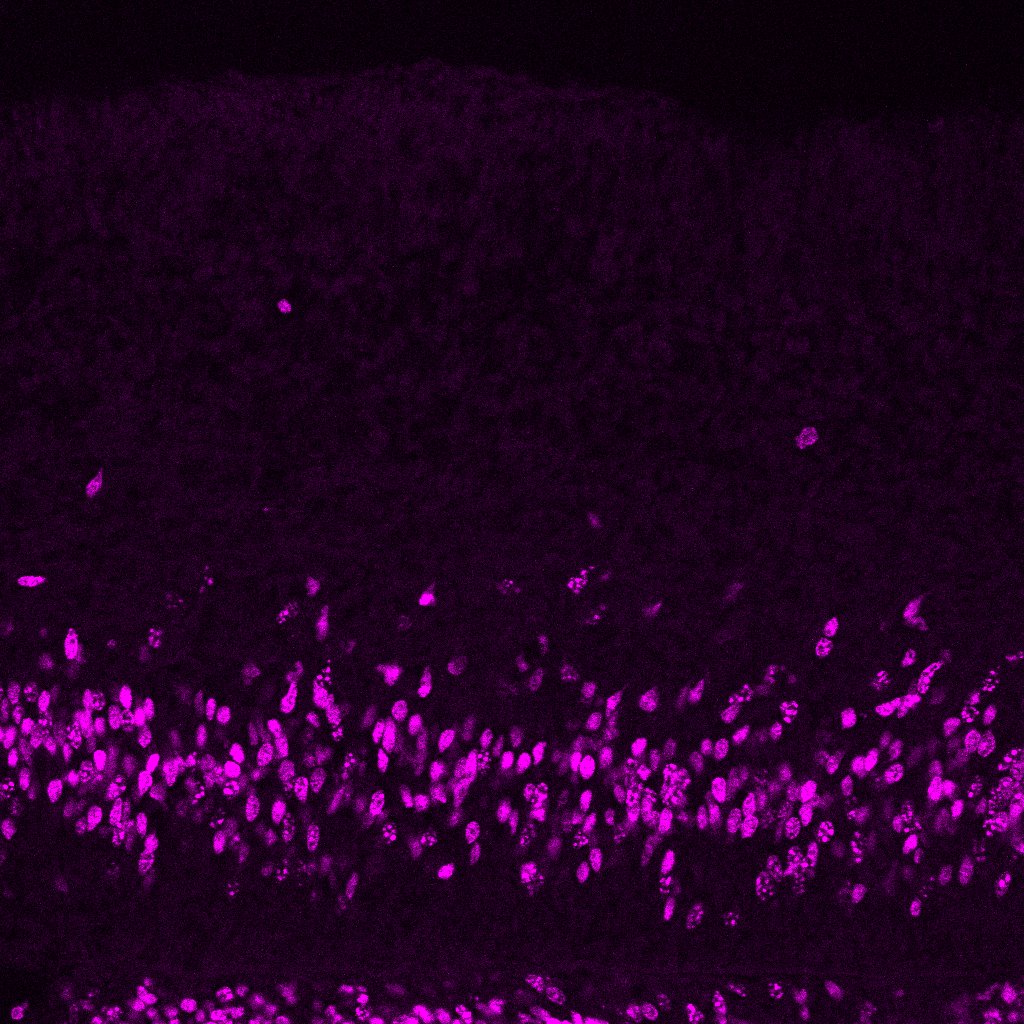

Supplement: Supplementary file 7 — Source data Fig. 2 [file 44318_2024_343_MOESM7_ESM.zip › Figure2/2C/WT_EdU.jpg]

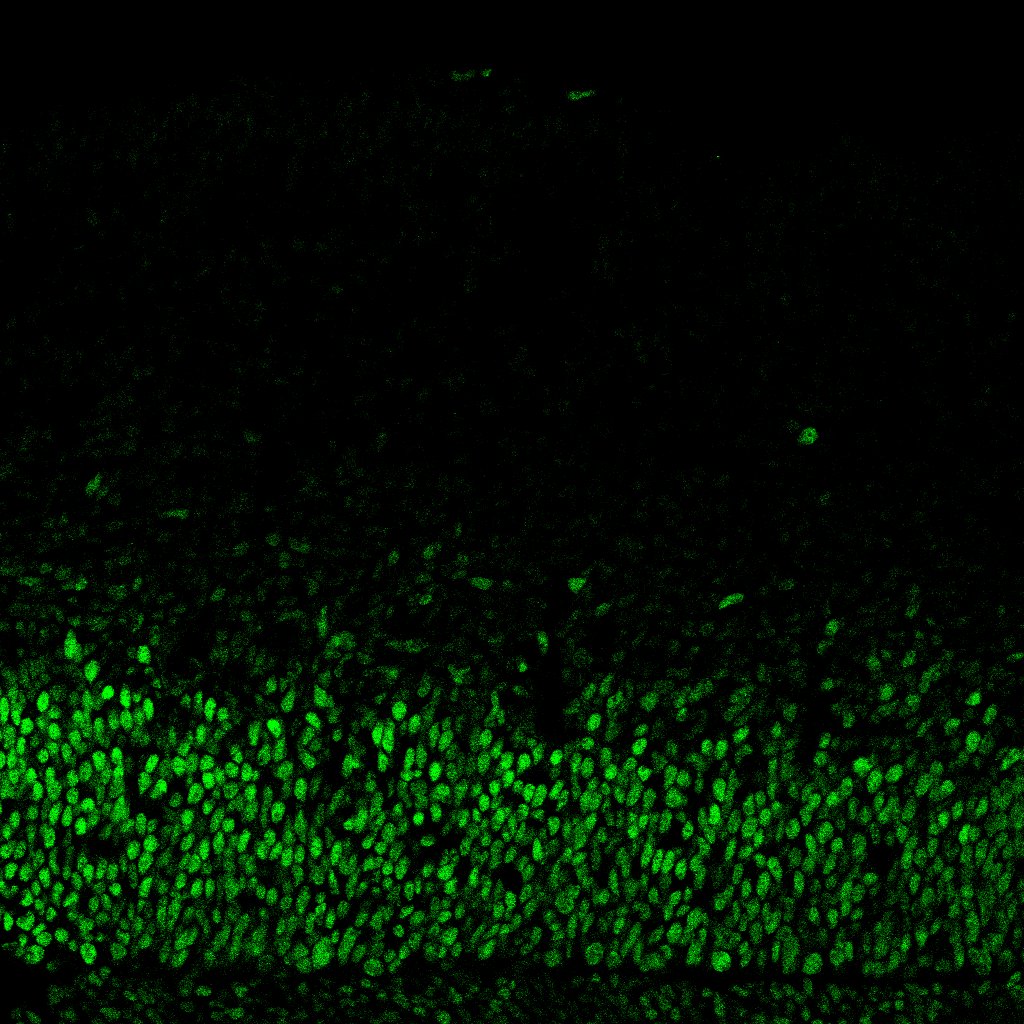

Supplement: Supplementary file 7 — Source data Fig. 2 [file 44318_2024_343_MOESM7_ESM.zip › Figure2/2C/WT_PAX6.jpg]

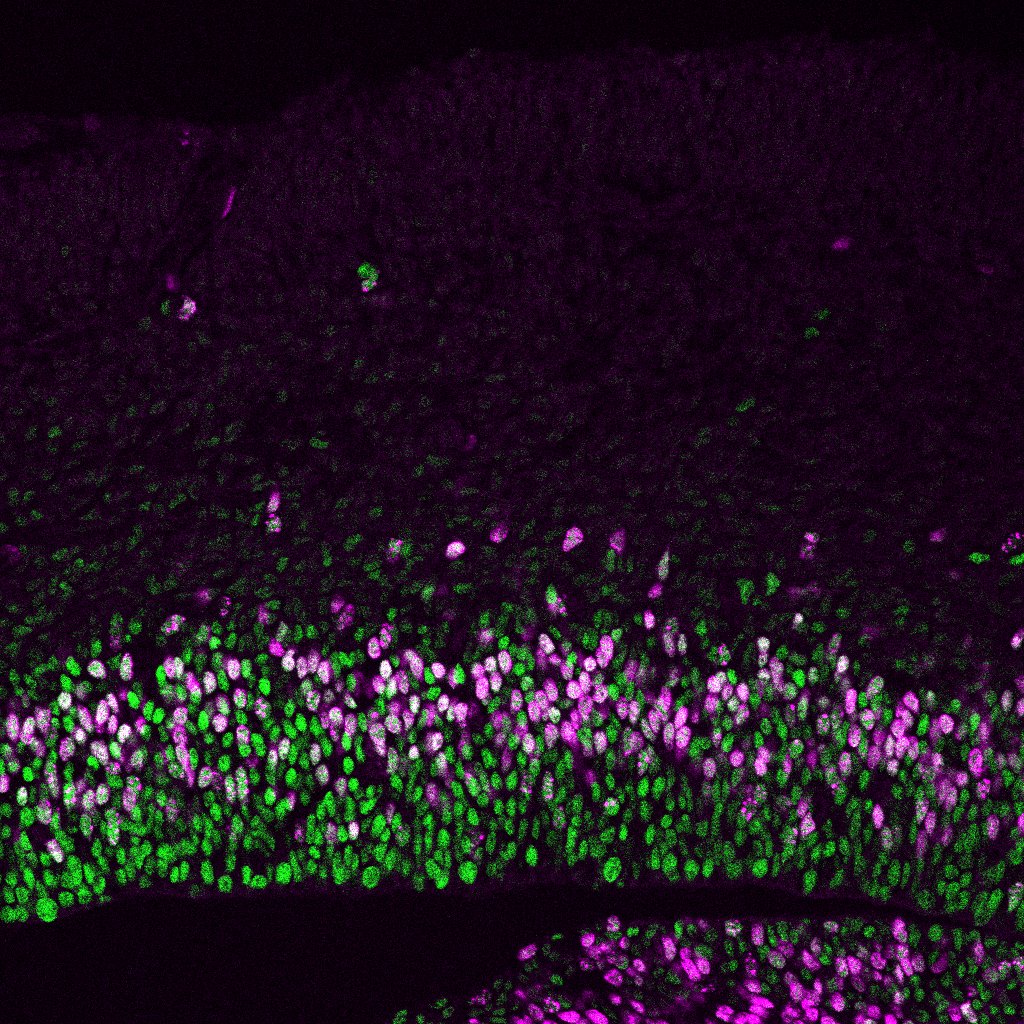

Supplement: Supplementary file 7 — Source data Fig. 2 [file 44318_2024_343_MOESM7_ESM.zip › Figure2/2C/Auts2-del8:del8_PAX6,EdU.jpg]

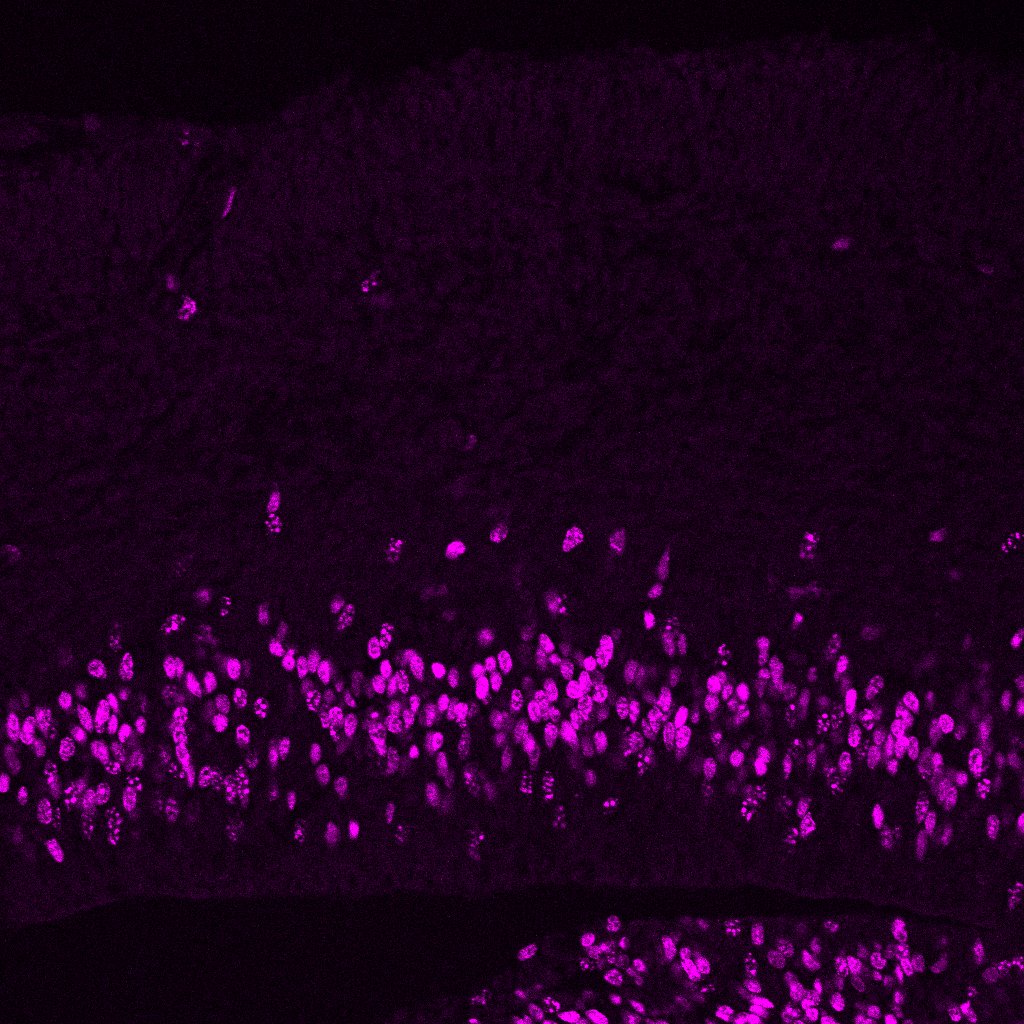

Supplement: Supplementary file 7 — Source data Fig. 2 [file 44318_2024_343_MOESM7_ESM.zip › Figure2/2C/Auts2-del8:del8_EdU.jpg]

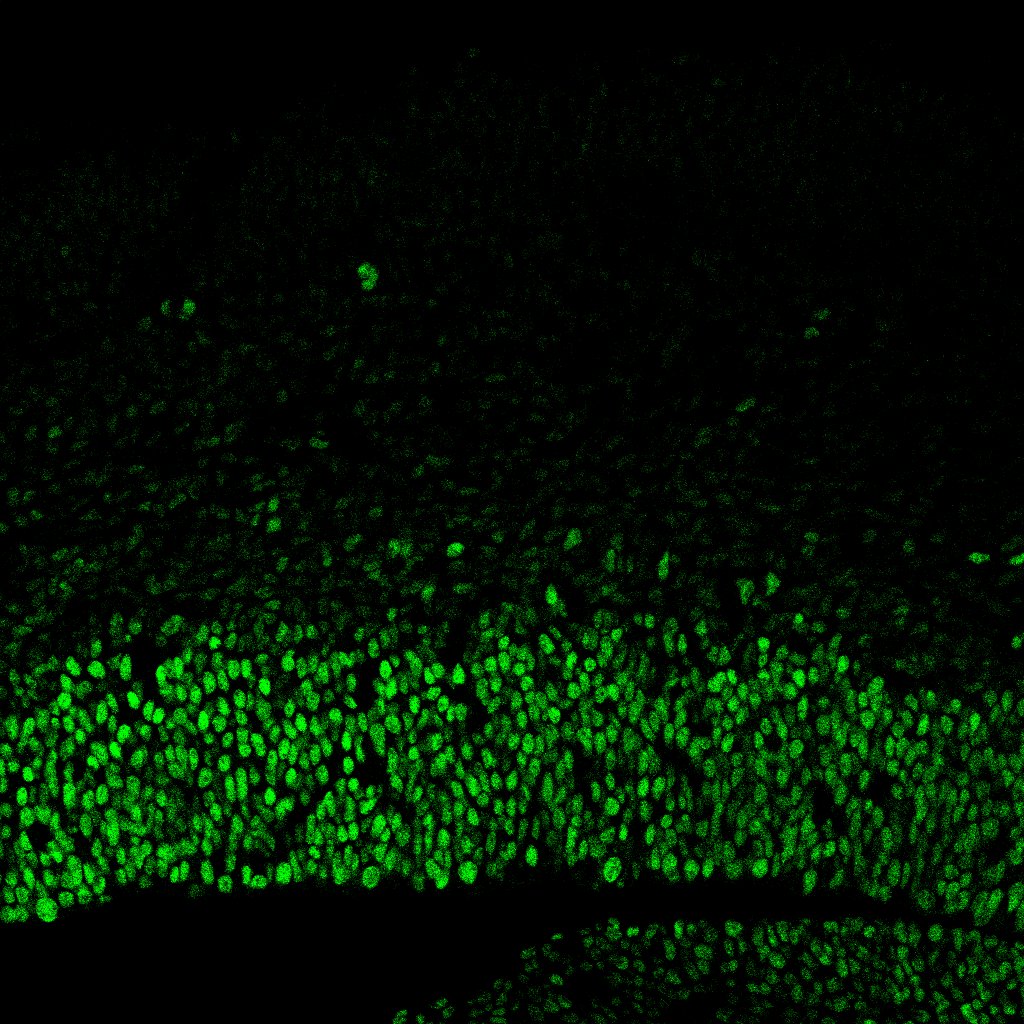

Supplement: Supplementary file 7 — Source data Fig. 2 [file 44318_2024_343_MOESM7_ESM.zip › Figure2/2C/Auts2-del8:del8_PAX6.jpg]

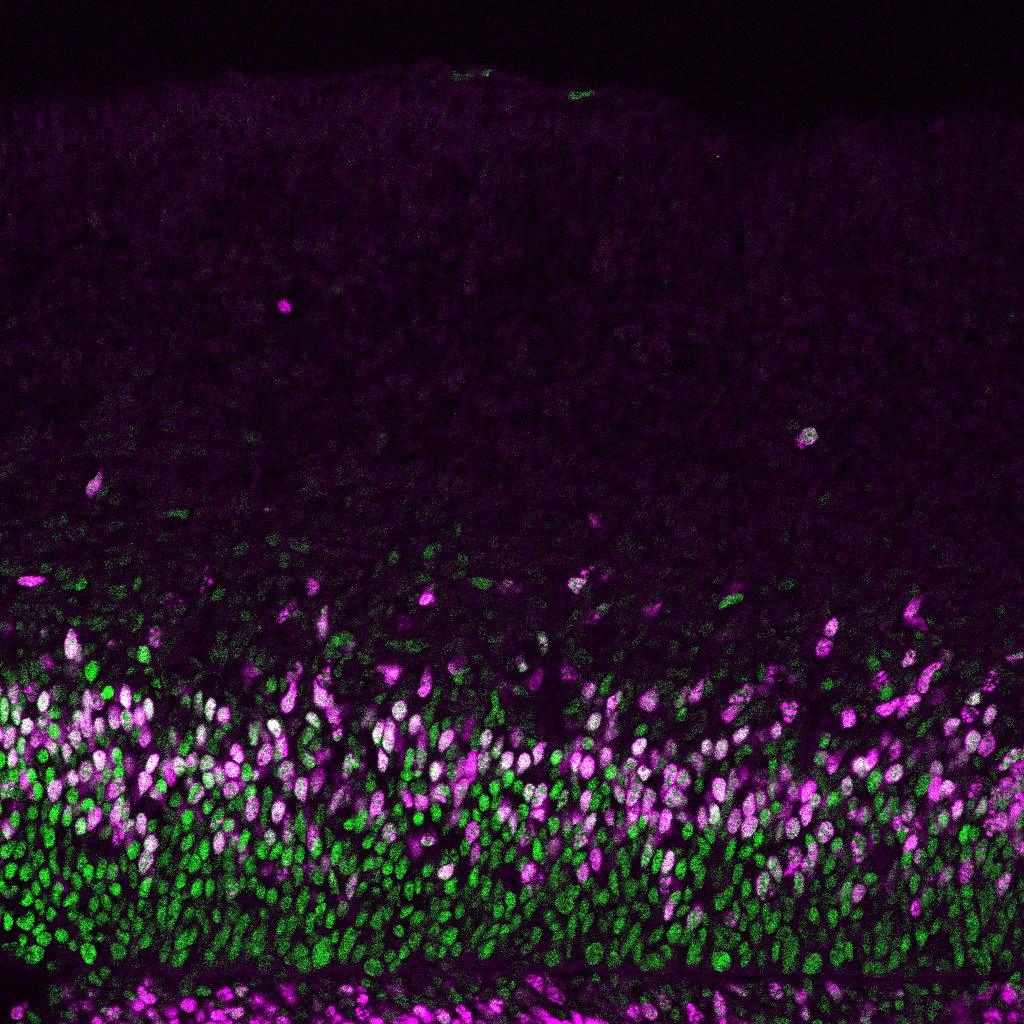

Supplement: Supplementary file 7 — Source data Fig. 2 [file 44318_2024_343_MOESM7_ESM.zip › Figure2/2C/WT_PAX6,EdU.jpg]

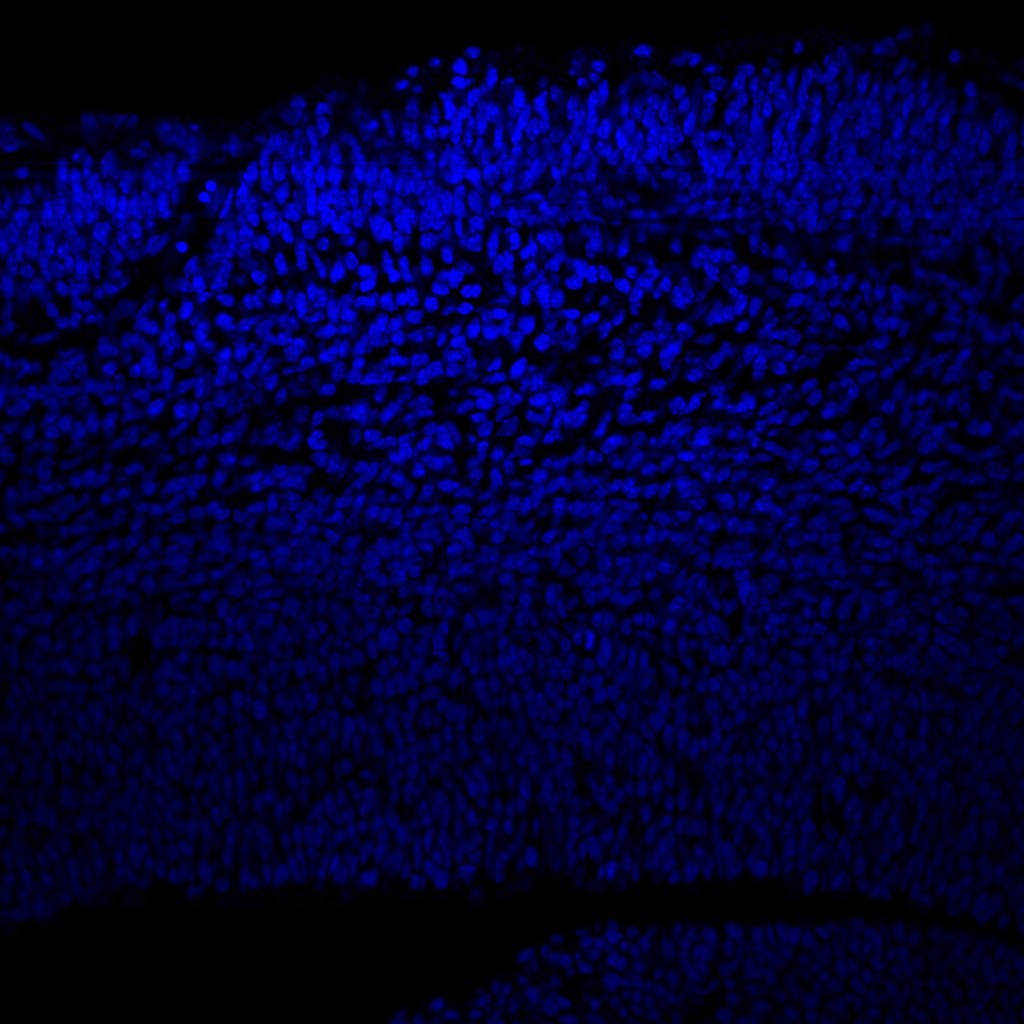

Supplement: Supplementary file 7 — Source data Fig. 2 [file 44318_2024_343_MOESM7_ESM.zip › Figure2/2C/Auts2-del8:del8_DAPI.jpg]

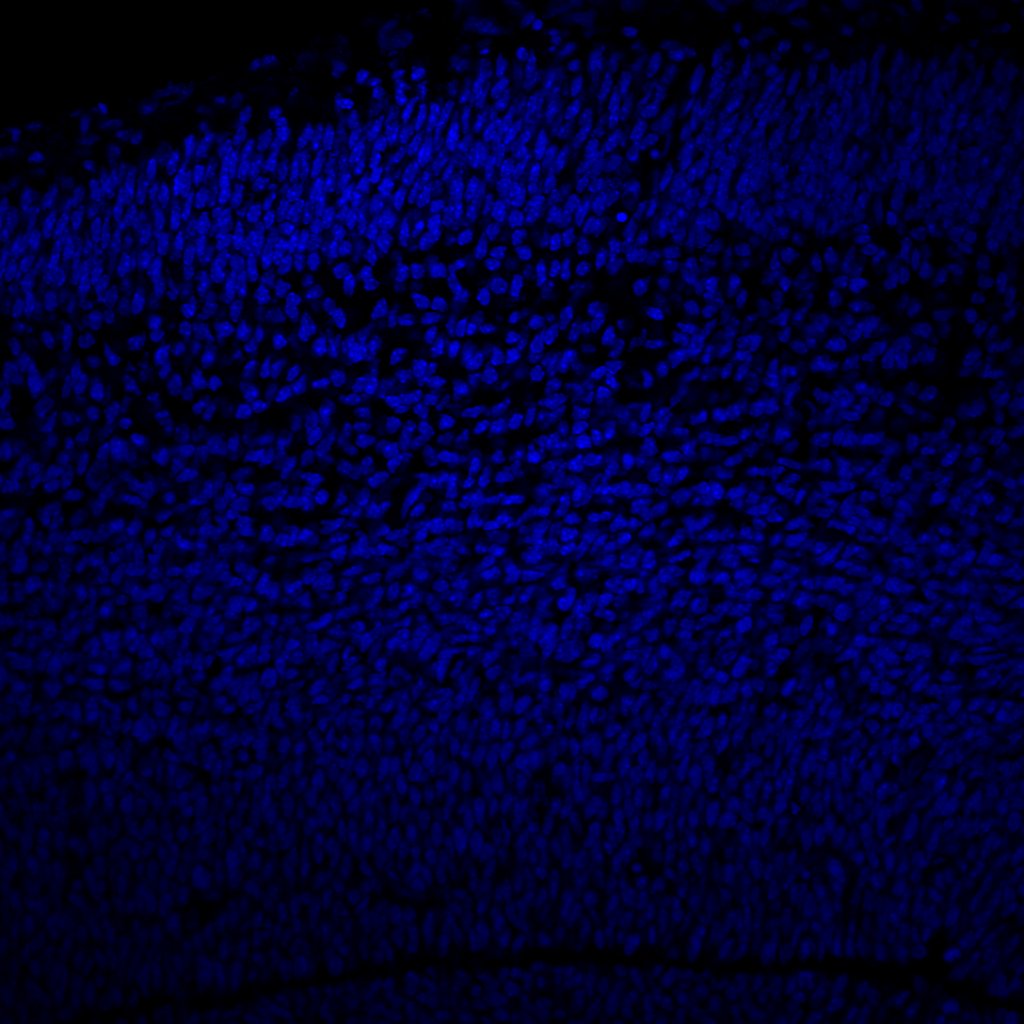

Supplement: Supplementary file 7 — Source data Fig. 2 [file 44318_2024_343_MOESM7_ESM.zip › Figure2/2D/WT_DAPI.jpg]

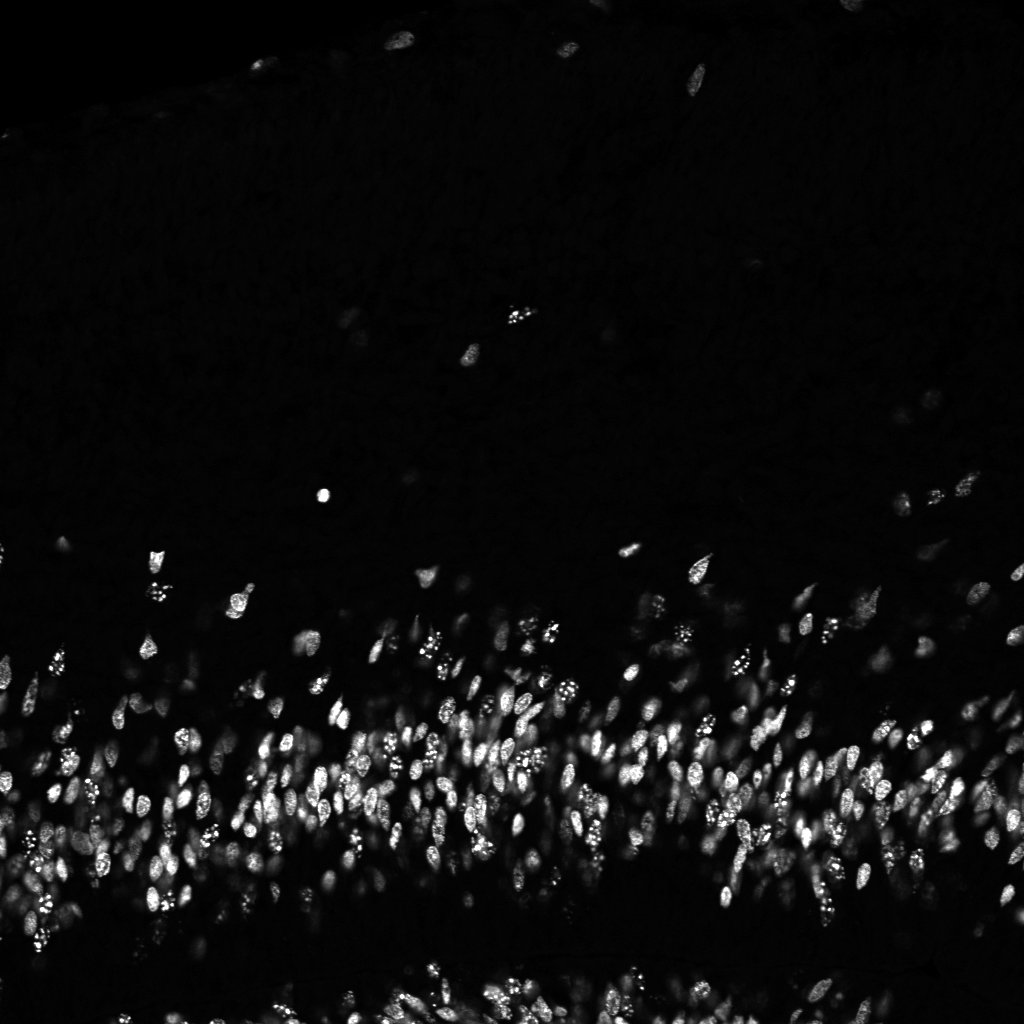

Supplement: Supplementary file 7 — Source data Fig. 2 [file 44318_2024_343_MOESM7_ESM.zip › Figure2/2D/WT_EdU.jpg]

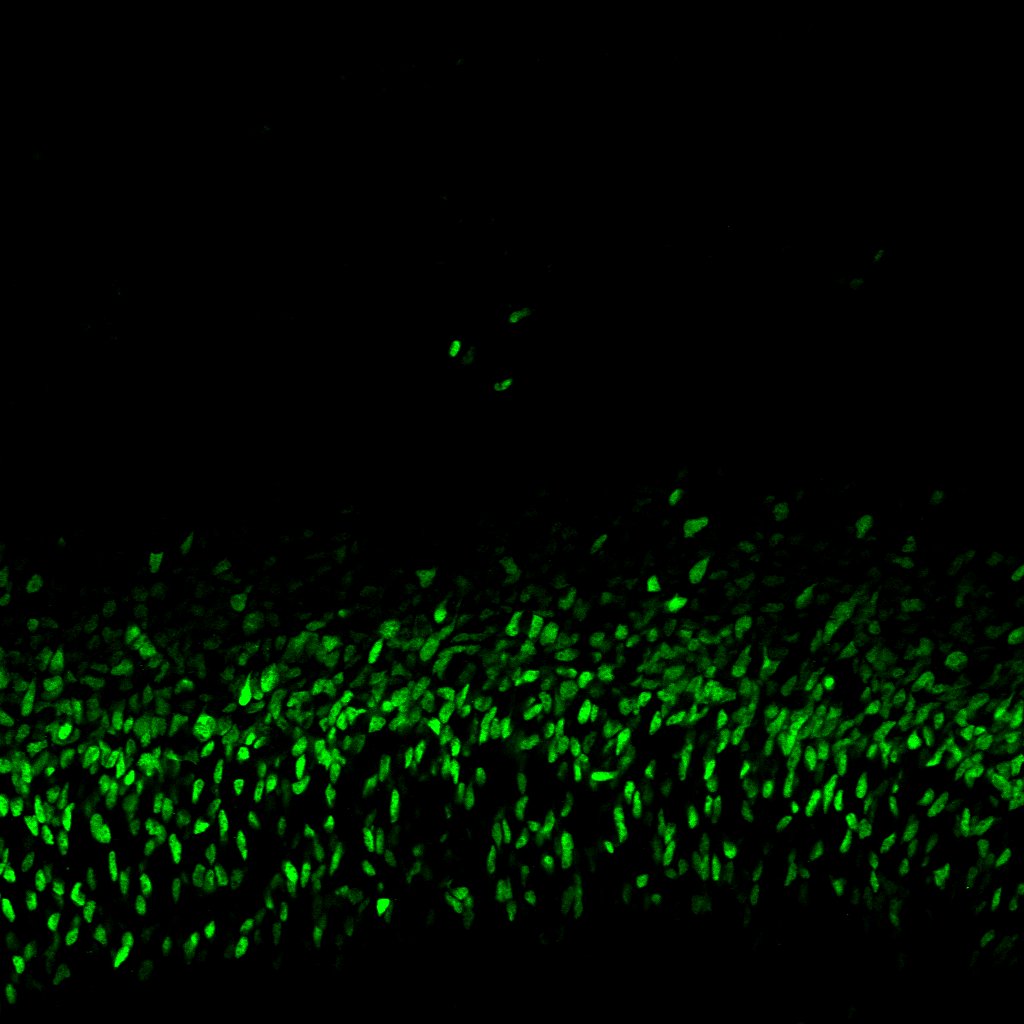

Supplement: Supplementary file 7 — Source data Fig. 2 [file 44318_2024_343_MOESM7_ESM.zip › Figure2/2D/WT_TBR2.jpg]

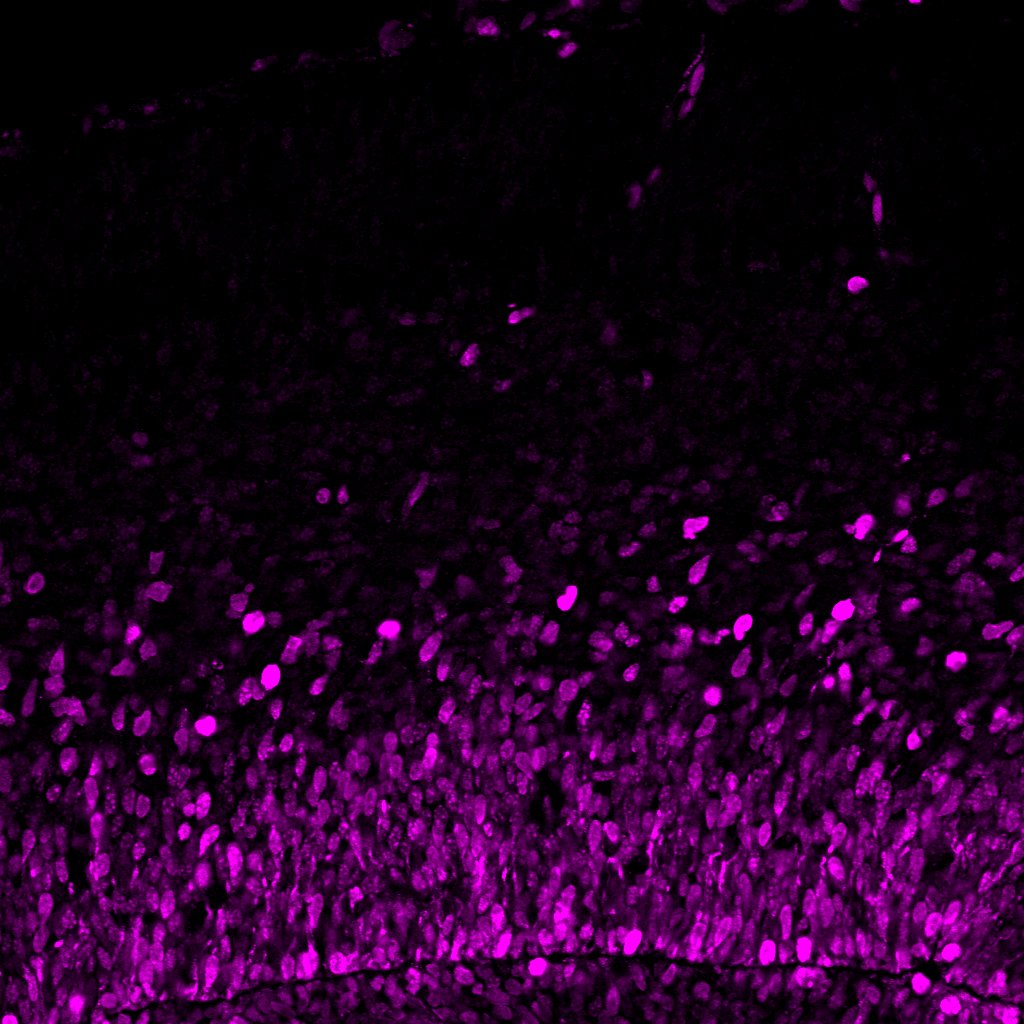

Supplement: Supplementary file 7 — Source data Fig. 2 [file 44318_2024_343_MOESM7_ESM.zip › Figure2/2D/WT_KI67.jpg]

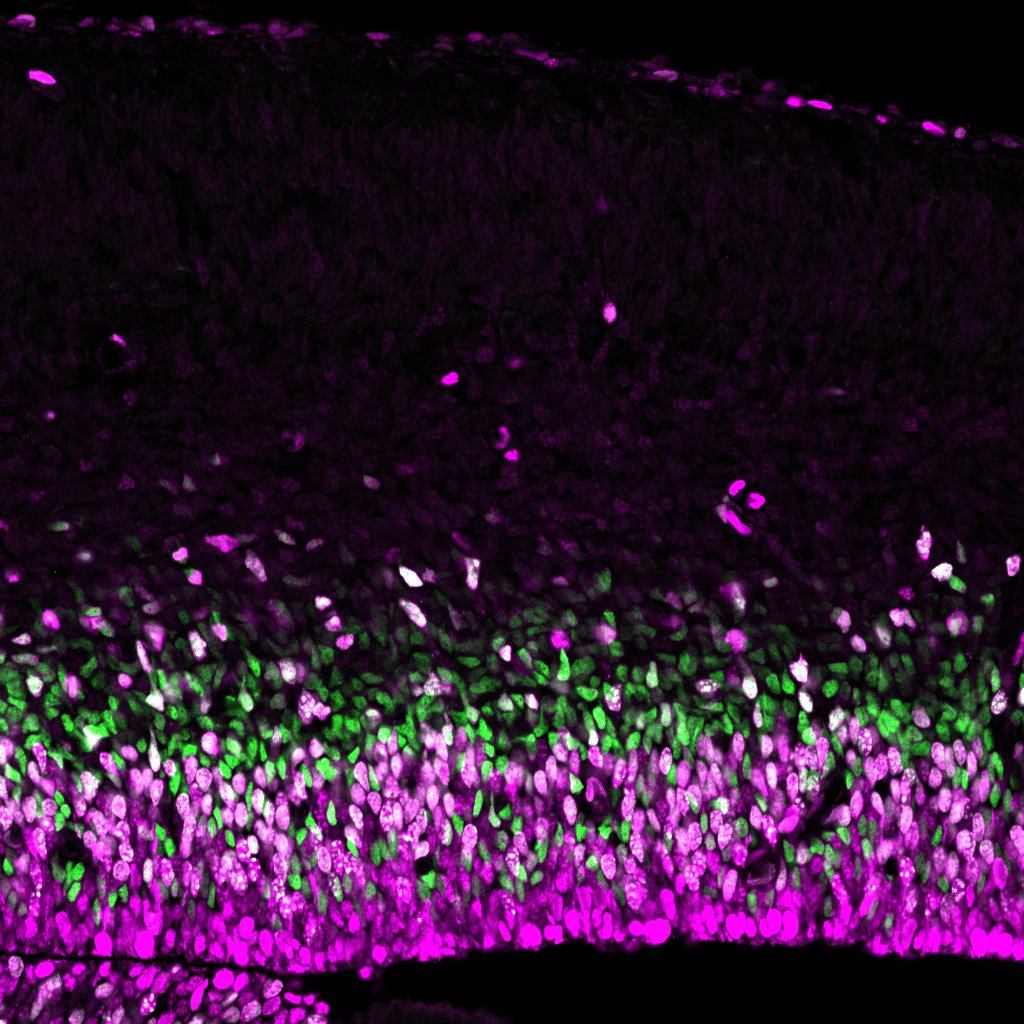

Supplement: Supplementary file 7 — Source data Fig. 2 [file 44318_2024_343_MOESM7_ESM.zip › Figure2/2D/Auts2-del8:del8_merge.jpg]

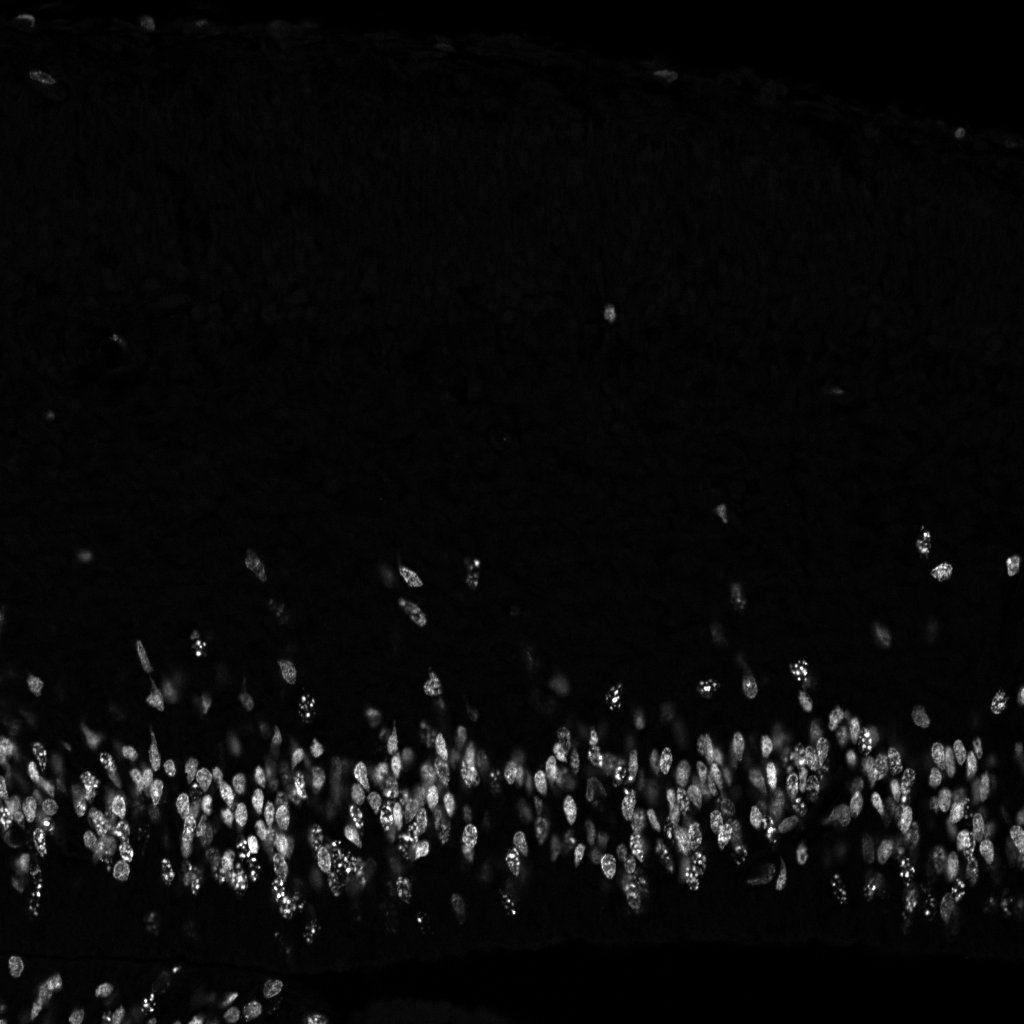

Supplement: Supplementary file 7 — Source data Fig. 2 [file 44318_2024_343_MOESM7_ESM.zip › Figure2/2D/Auts2-del8:del8_EdU.jpg]

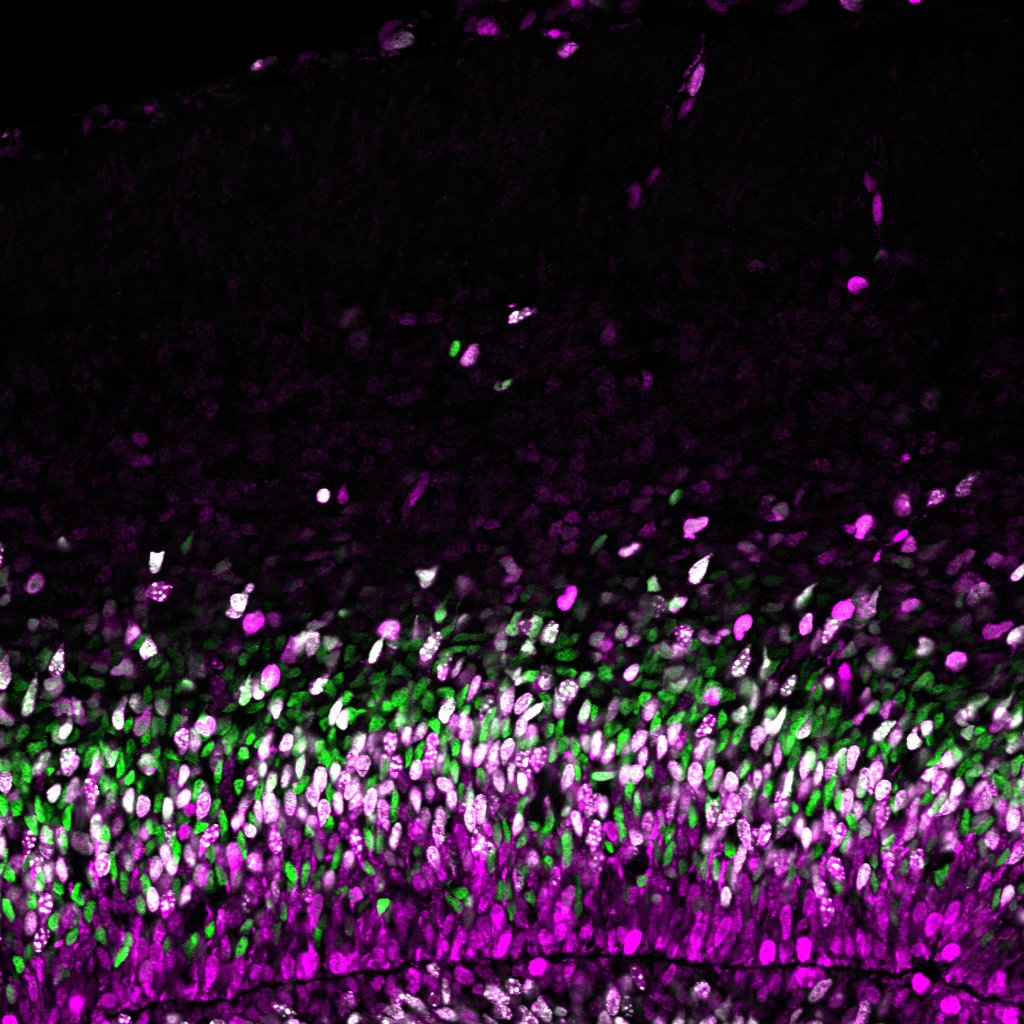

Supplement: Supplementary file 7 — Source data Fig. 2 [file 44318_2024_343_MOESM7_ESM.zip › Figure2/2D/WT_merge.jpg]

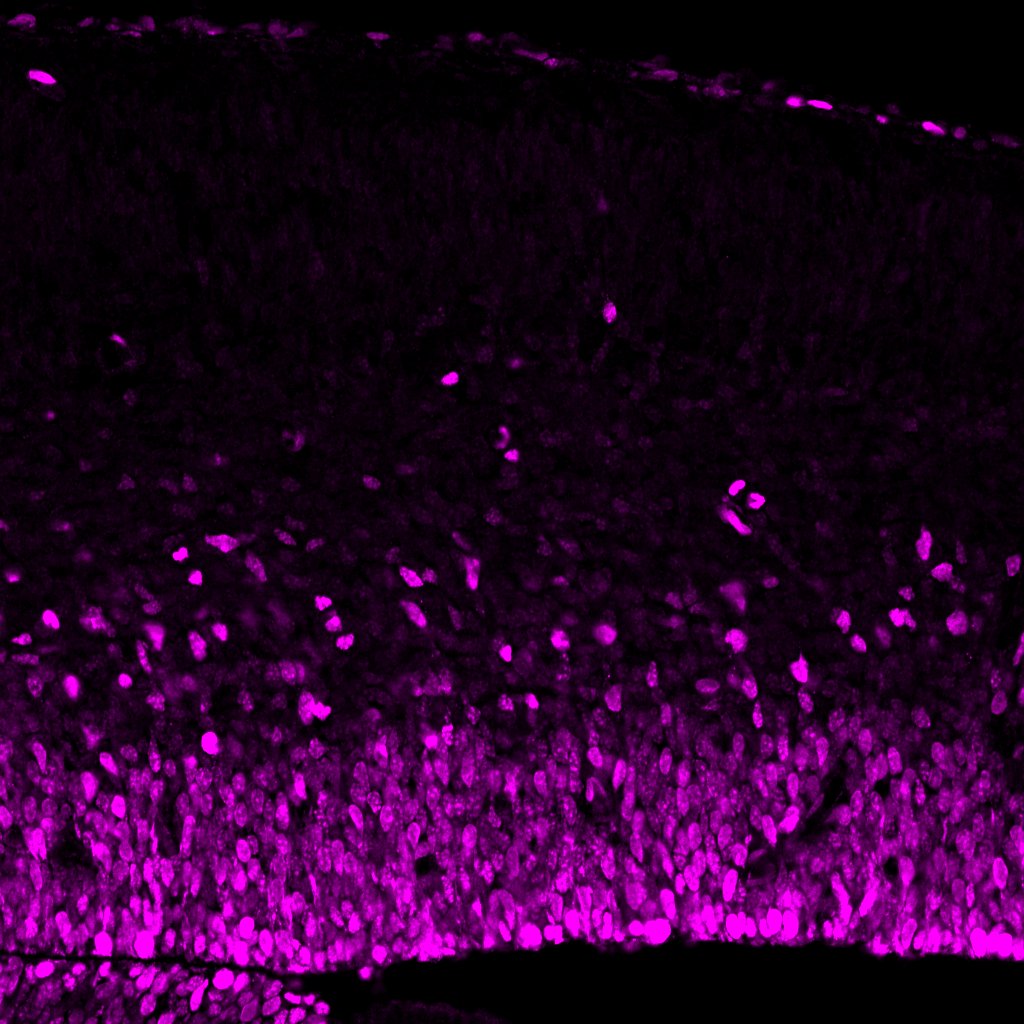

Supplement: Supplementary file 7 — Source data Fig. 2 [file 44318_2024_343_MOESM7_ESM.zip › Figure2/2D/Auts2-del8:del8_KI67.jpg]

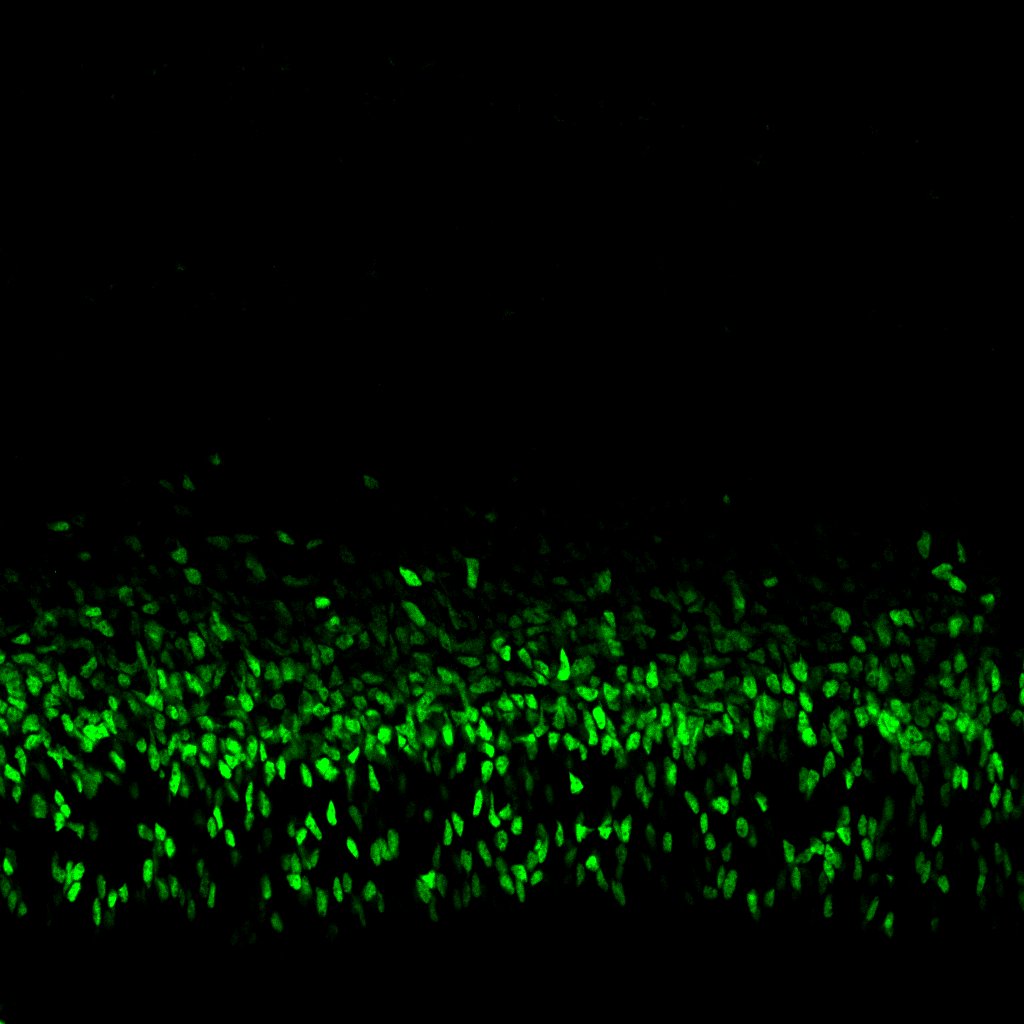

Supplement: Supplementary file 7 — Source data Fig. 2 [file 44318_2024_343_MOESM7_ESM.zip › Figure2/2D/Auts2-del8:del8_TBR2.jpg]

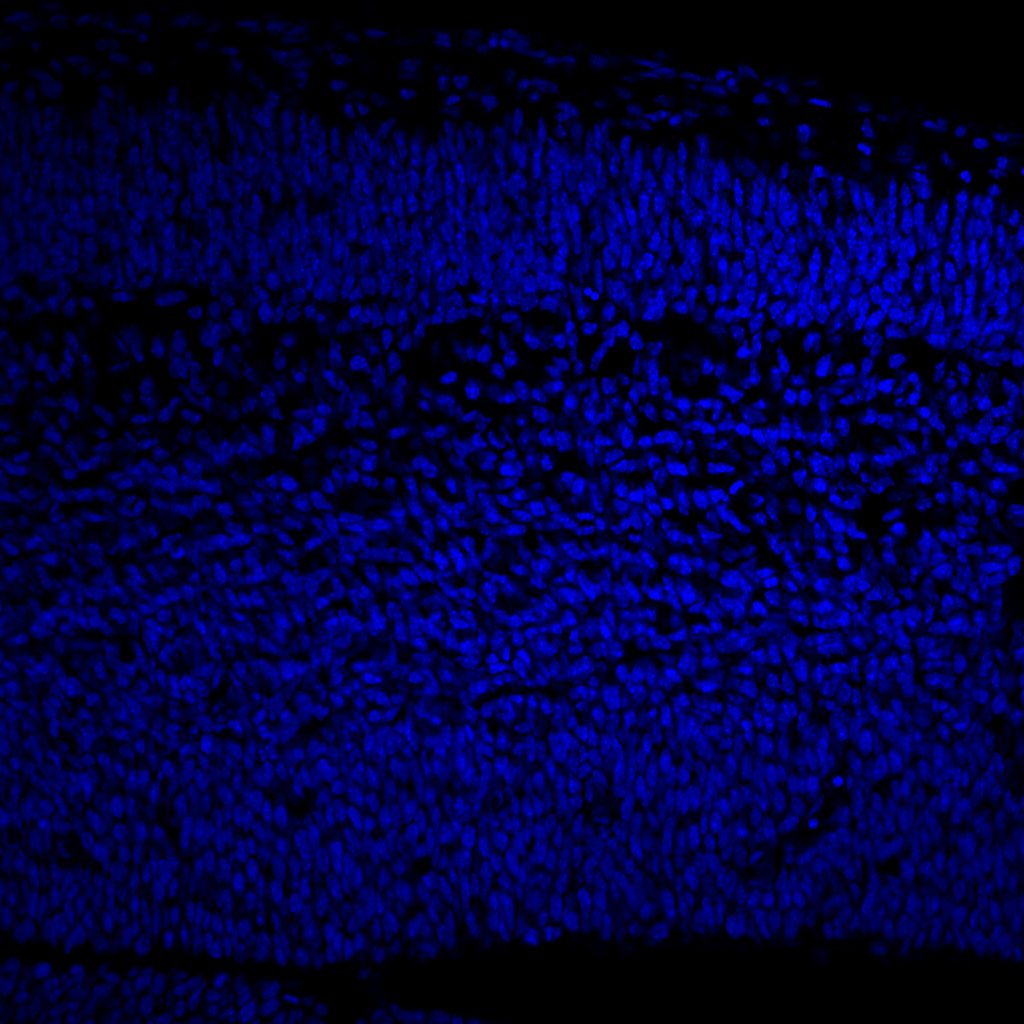

Supplement: Supplementary file 7 — Source data Fig. 2 [file 44318_2024_343_MOESM7_ESM.zip › Figure2/2D/Auts2-del8:del8_DAPI.jpg]

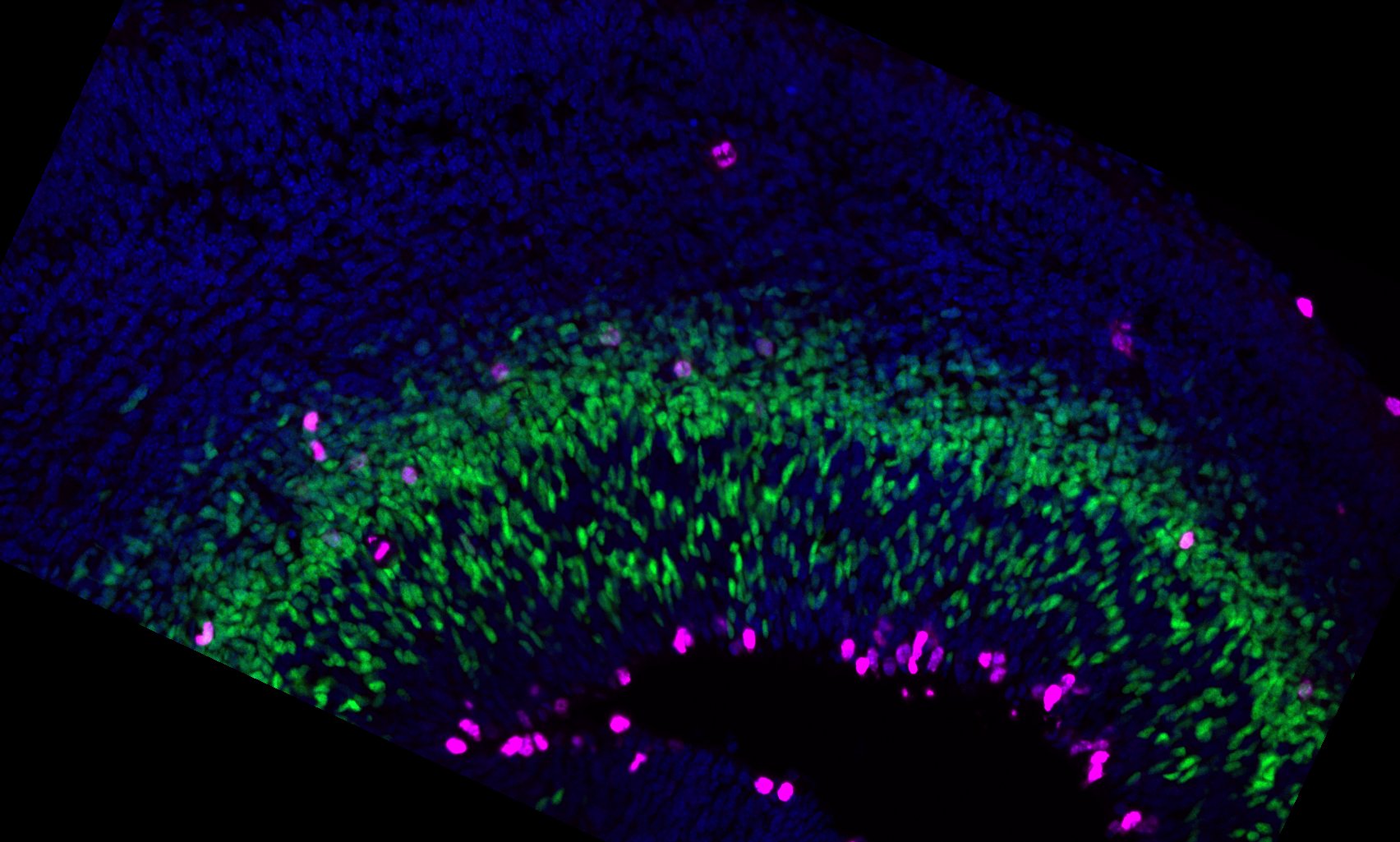

Supplement: Supplementary file 7 — Source data Fig. 2 [file 44318_2024_343_MOESM7_ESM.zip › Figure2/2B/Homo.jpg]

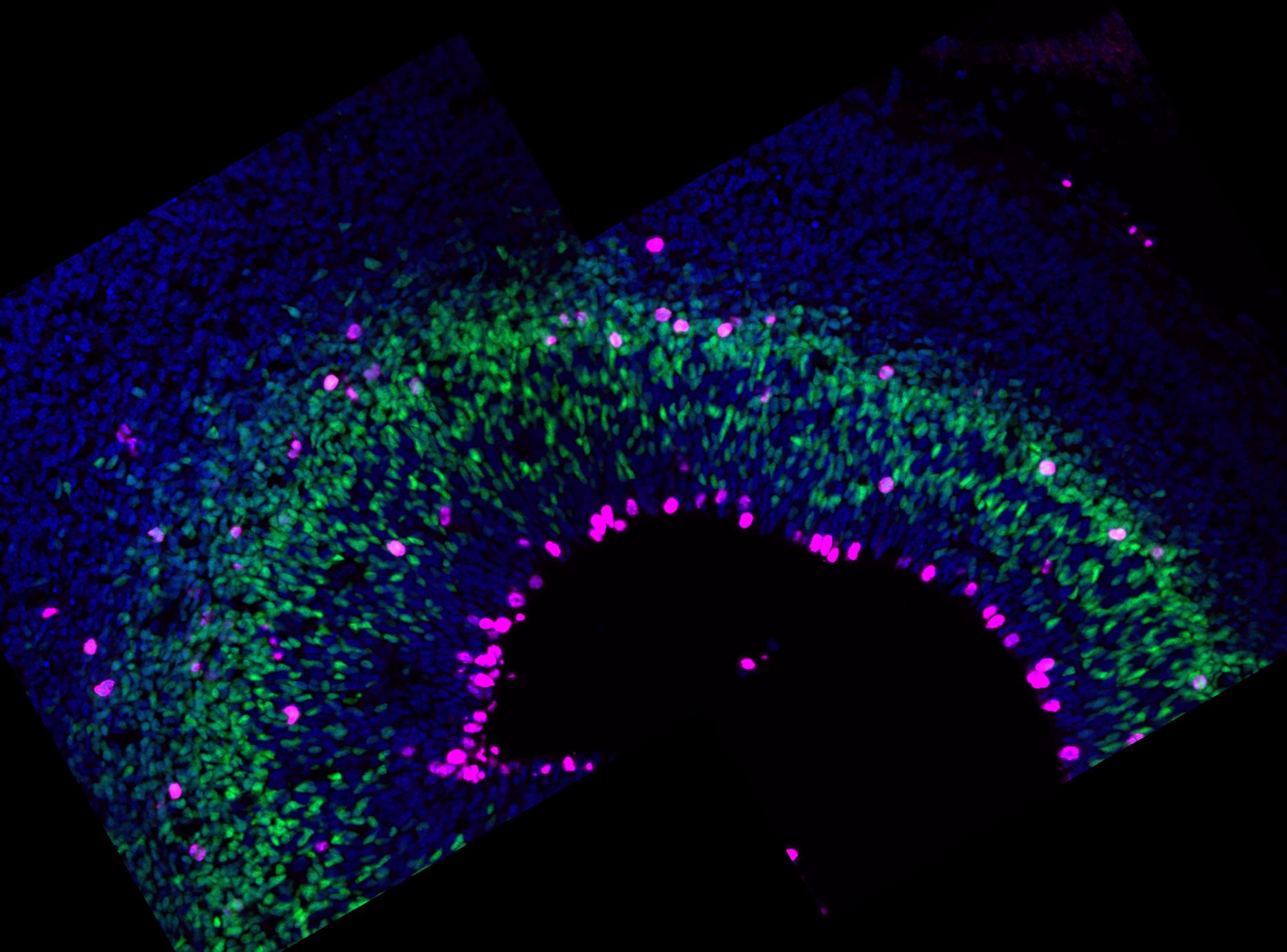

Supplement: Supplementary file 7 — Source data Fig. 2 [file 44318_2024_343_MOESM7_ESM.zip › Figure2/2B/WT.jpg]

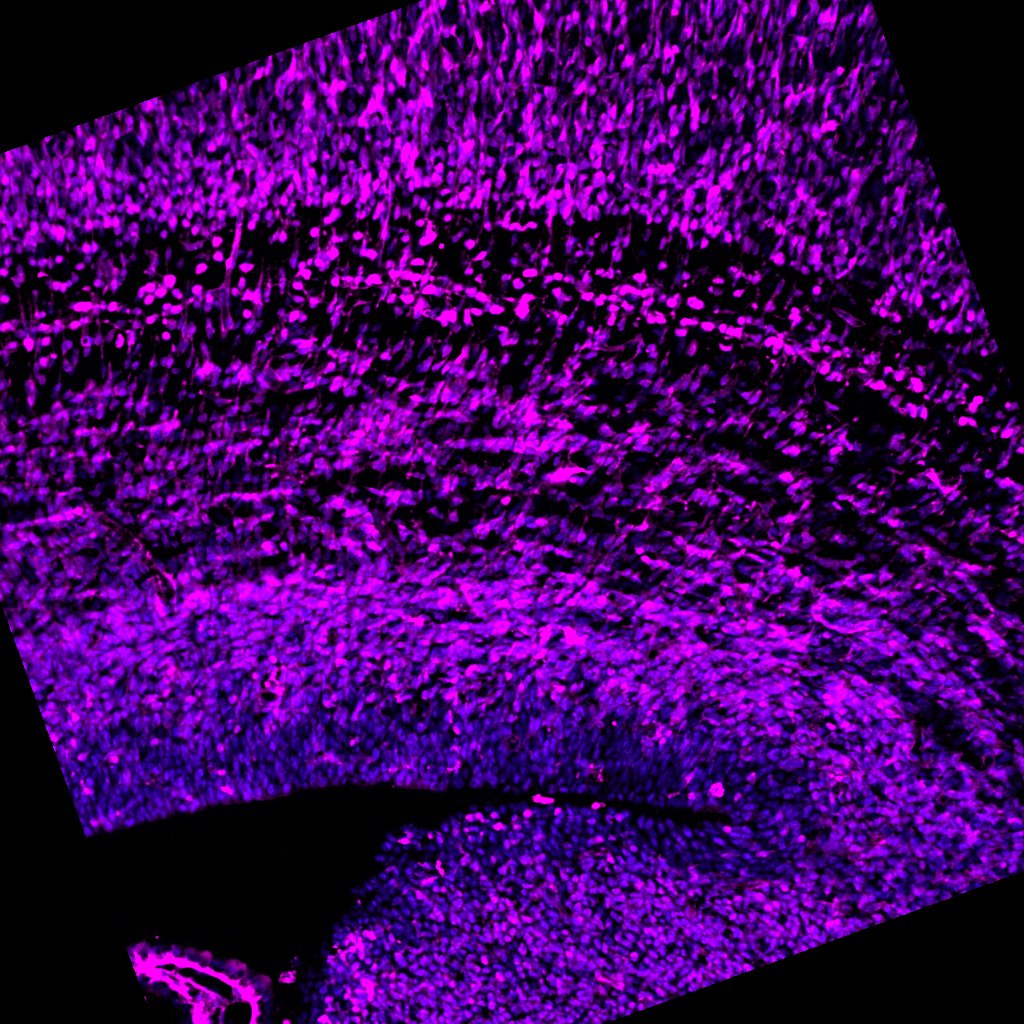

Supplement: Supplementary file 8 — Source data Fig. 3 [file 44318_2024_343_MOESM8_ESM.zip › Figure3/3A/shAuts2_DAPI,HuCD.jpg]

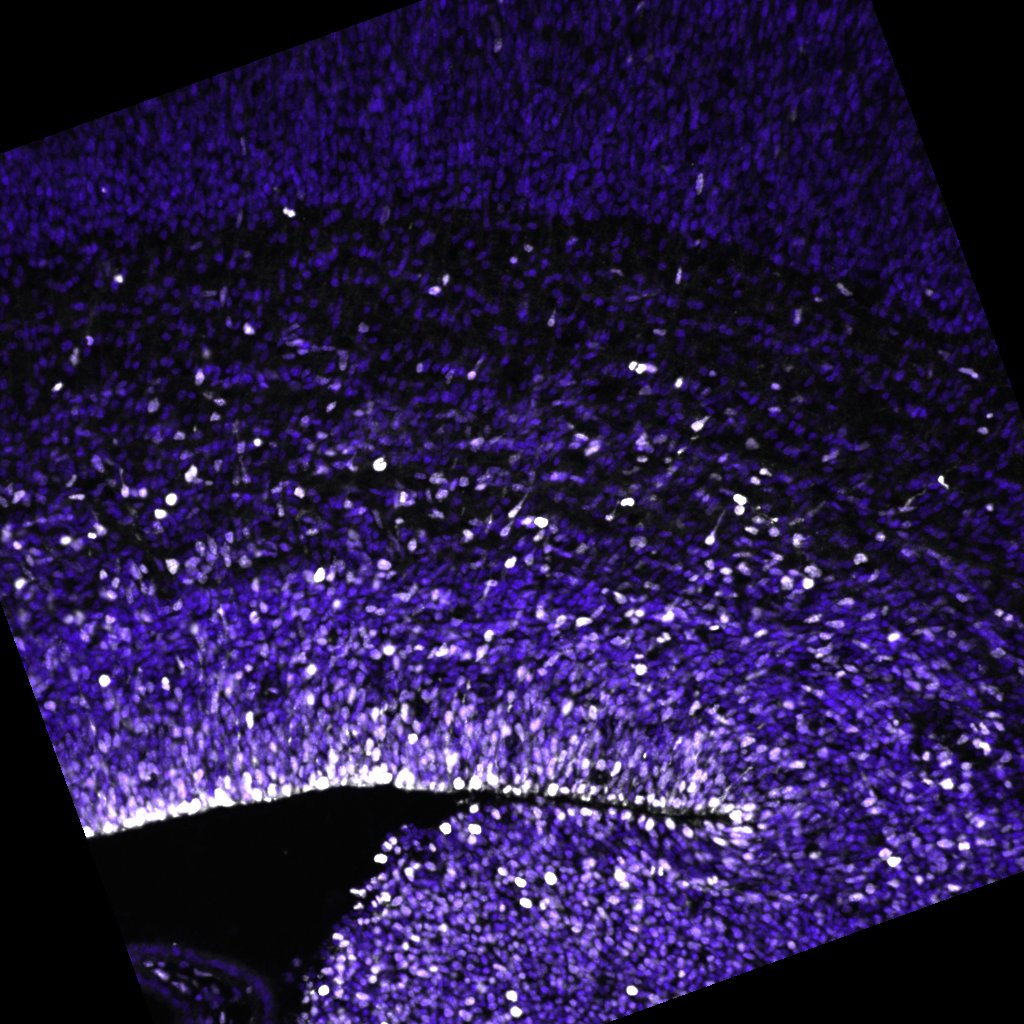

Supplement: Supplementary file 8 — Source data Fig. 3 [file 44318_2024_343_MOESM8_ESM.zip › Figure3/3A/shAuts2_DAPI,KI67.jpg]

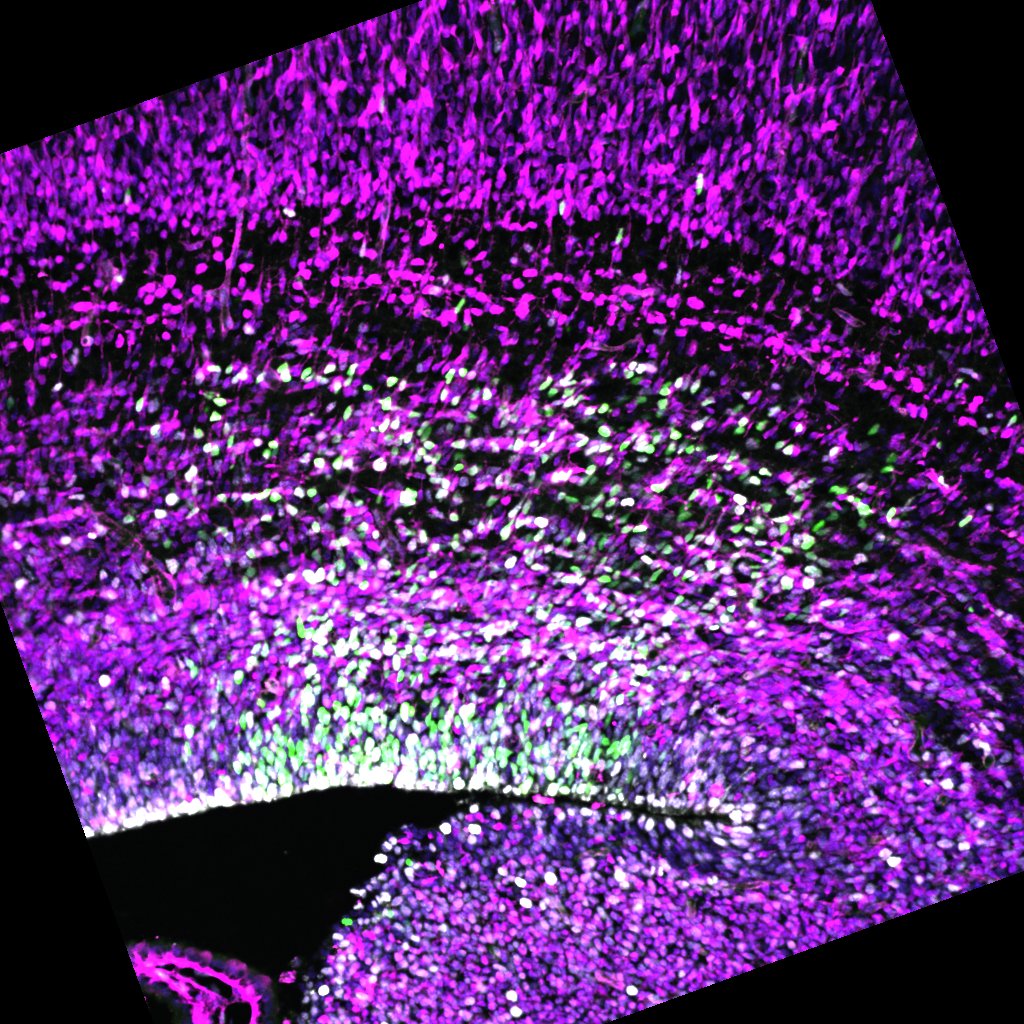

Supplement: Supplementary file 8 — Source data Fig. 3 [file 44318_2024_343_MOESM8_ESM.zip › Figure3/3A/shAuts2_merge.jpg]

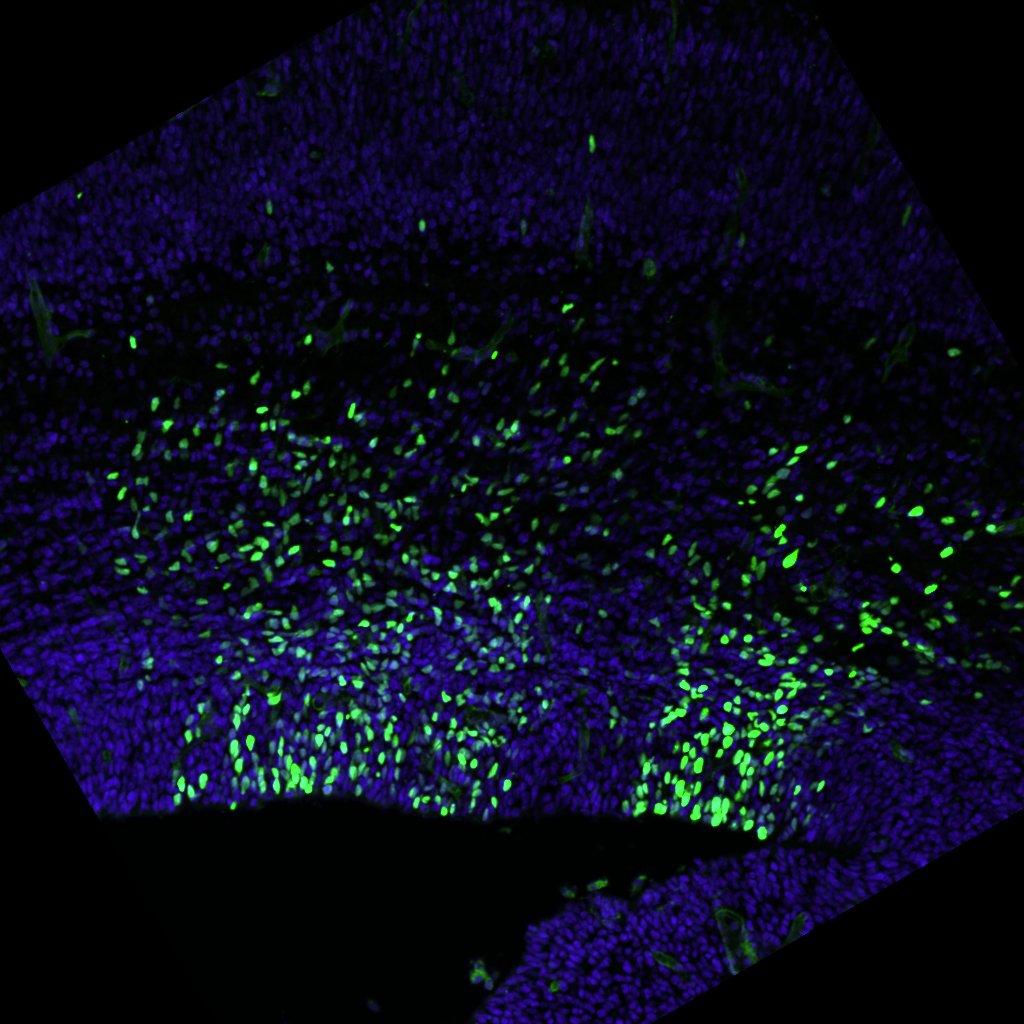

Supplement: Supplementary file 8 — Source data Fig. 3 [file 44318_2024_343_MOESM8_ESM.zip › Figure3/3A/shScramble_DAPI,GFP.jpg]

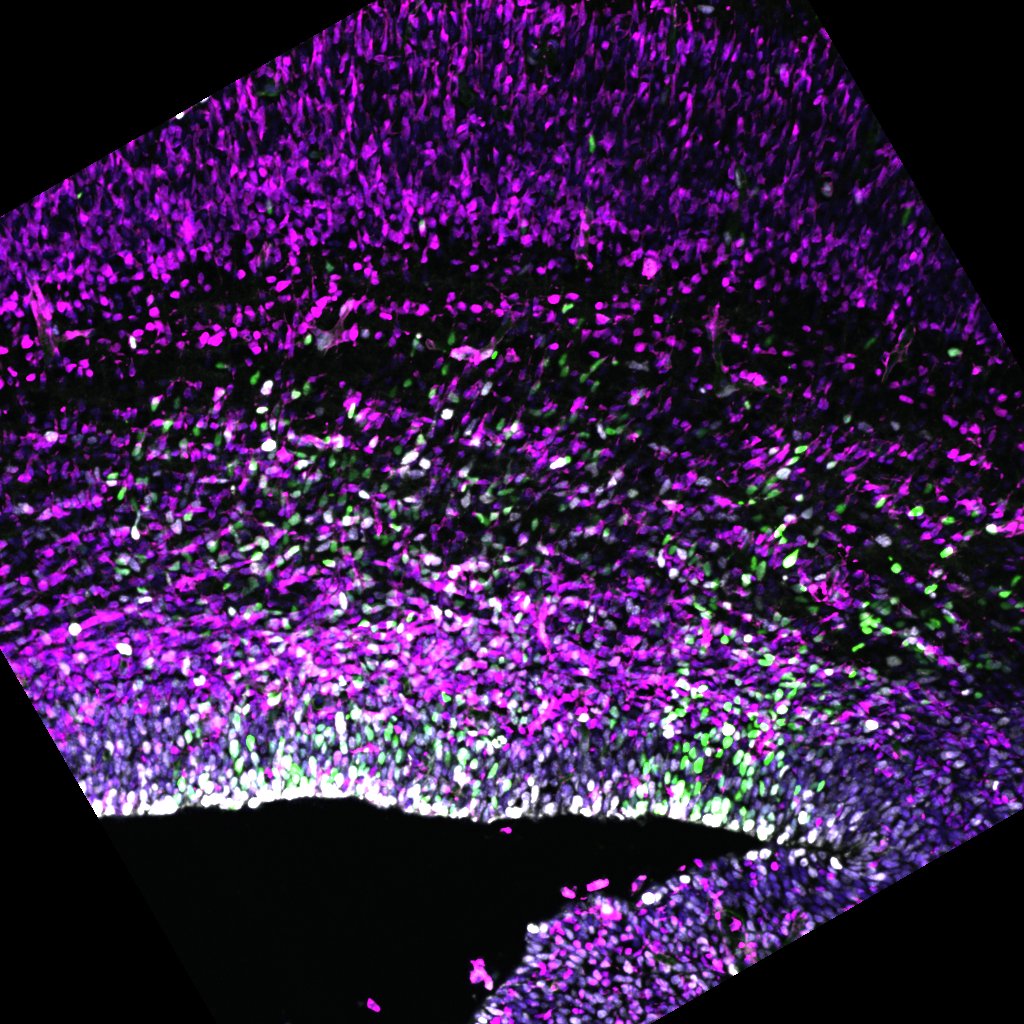

Supplement: Supplementary file 8 — Source data Fig. 3 [file 44318_2024_343_MOESM8_ESM.zip › Figure3/3A/shScramble_merge.jpg]

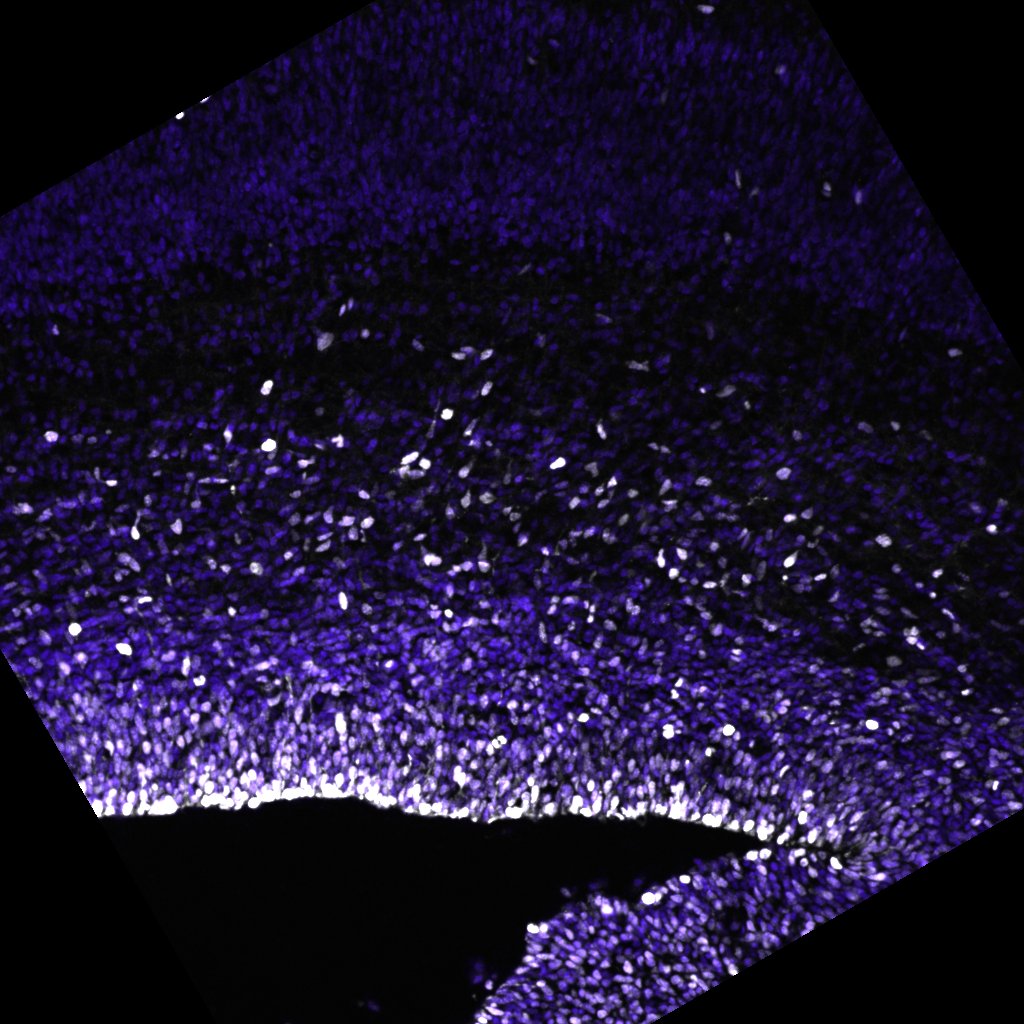

Supplement: Supplementary file 8 — Source data Fig. 3 [file 44318_2024_343_MOESM8_ESM.zip › Figure3/3A/shScramble_DAPI,KI67.jpg]

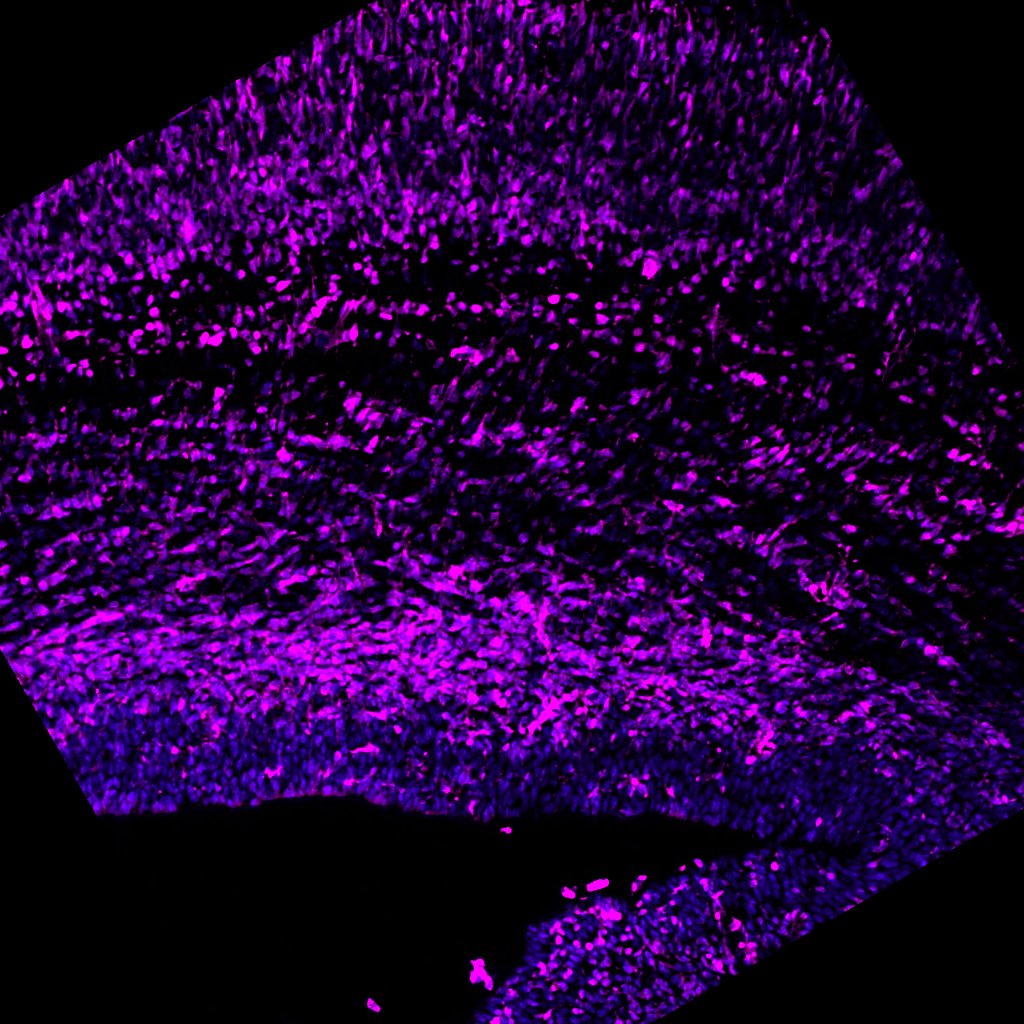

Supplement: Supplementary file 8 — Source data Fig. 3 [file 44318_2024_343_MOESM8_ESM.zip › Figure3/3A/shScramble_DAPI,HuCD.jpg]

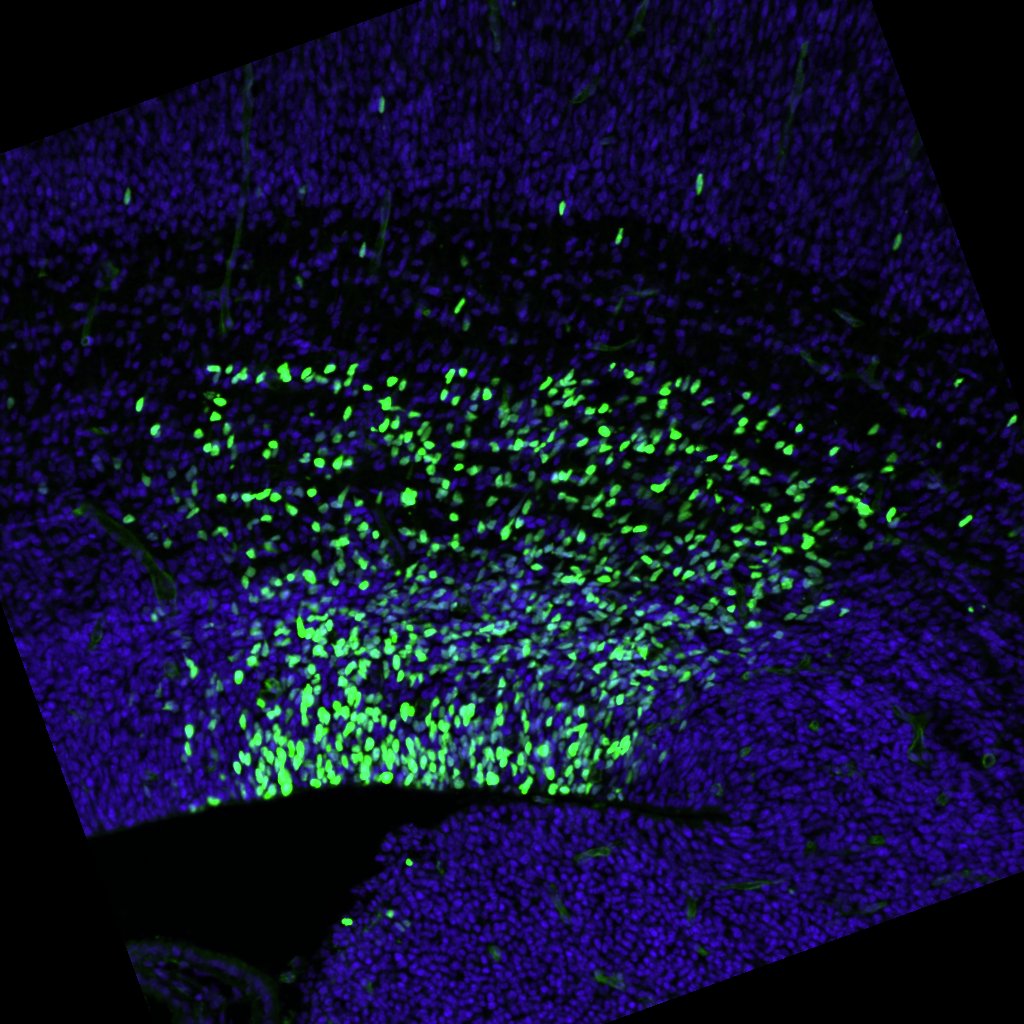

Supplement: Supplementary file 8 — Source data Fig. 3 [file 44318_2024_343_MOESM8_ESM.zip › Figure3/3A/shAuts2_DAPI,GFP.jpg]

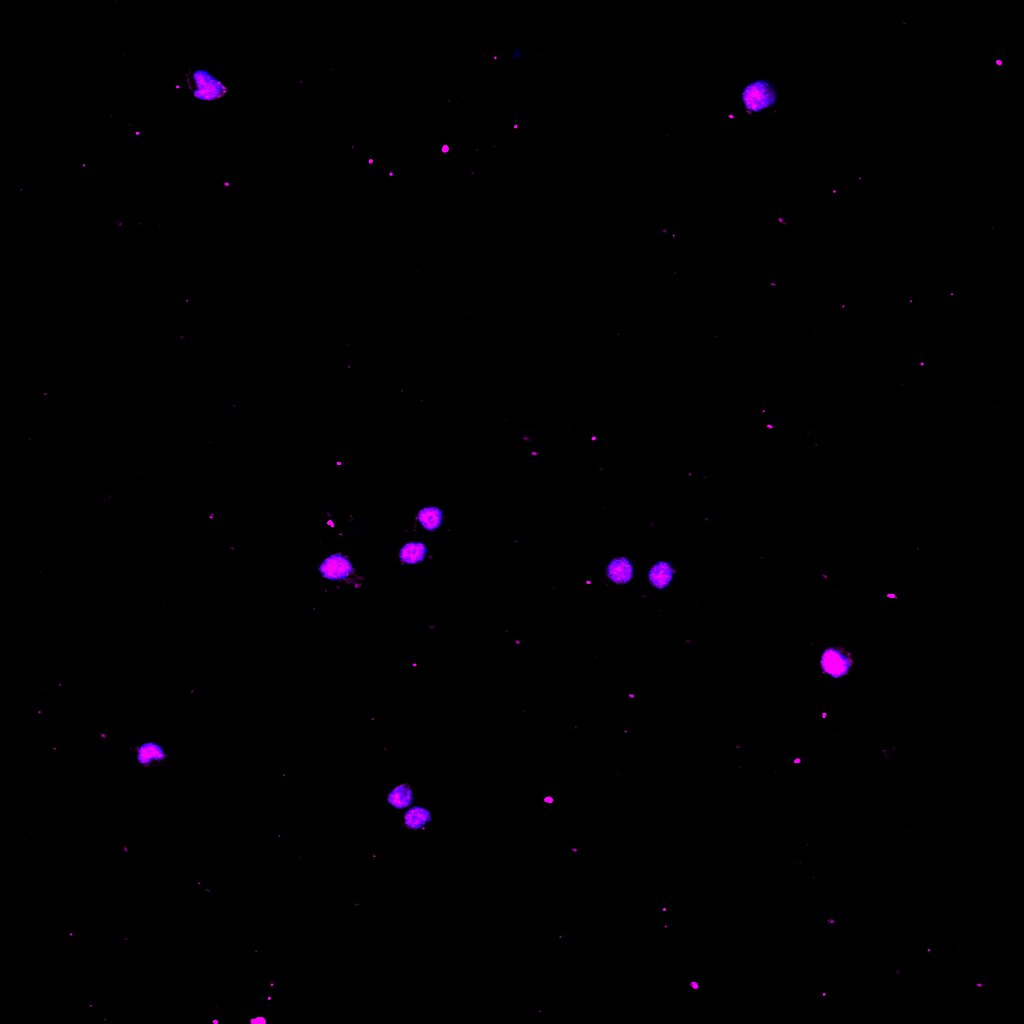

Supplement: Supplementary file 9 — Source data Fig. 4 [file 44318_2024_343_MOESM9_ESM.zip › Figure4/4D/CD133_DAPI,SOX2.jpg]

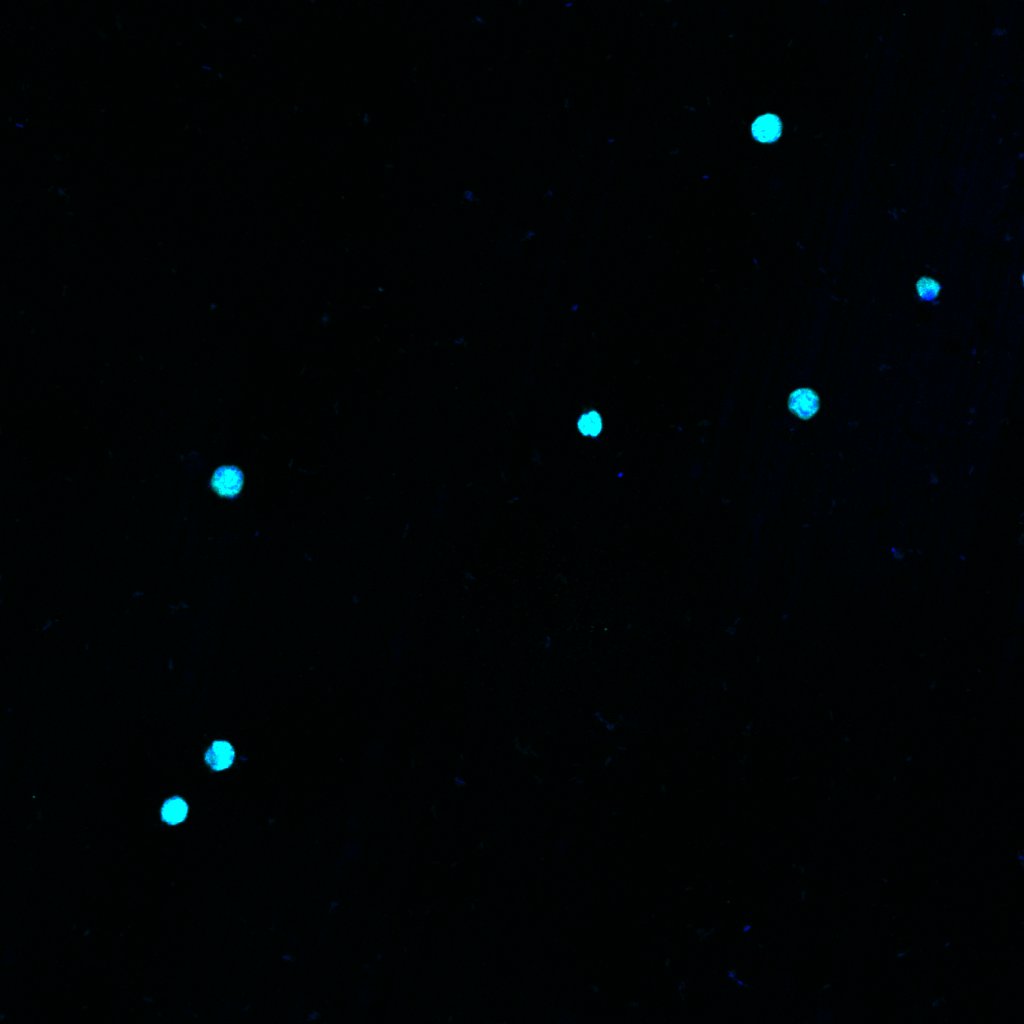

Supplement: Supplementary file 9 — Source data Fig. 4 [file 44318_2024_343_MOESM9_ESM.zip › Figure4/4D/EGFP_FAPI,TBR2.jpg]

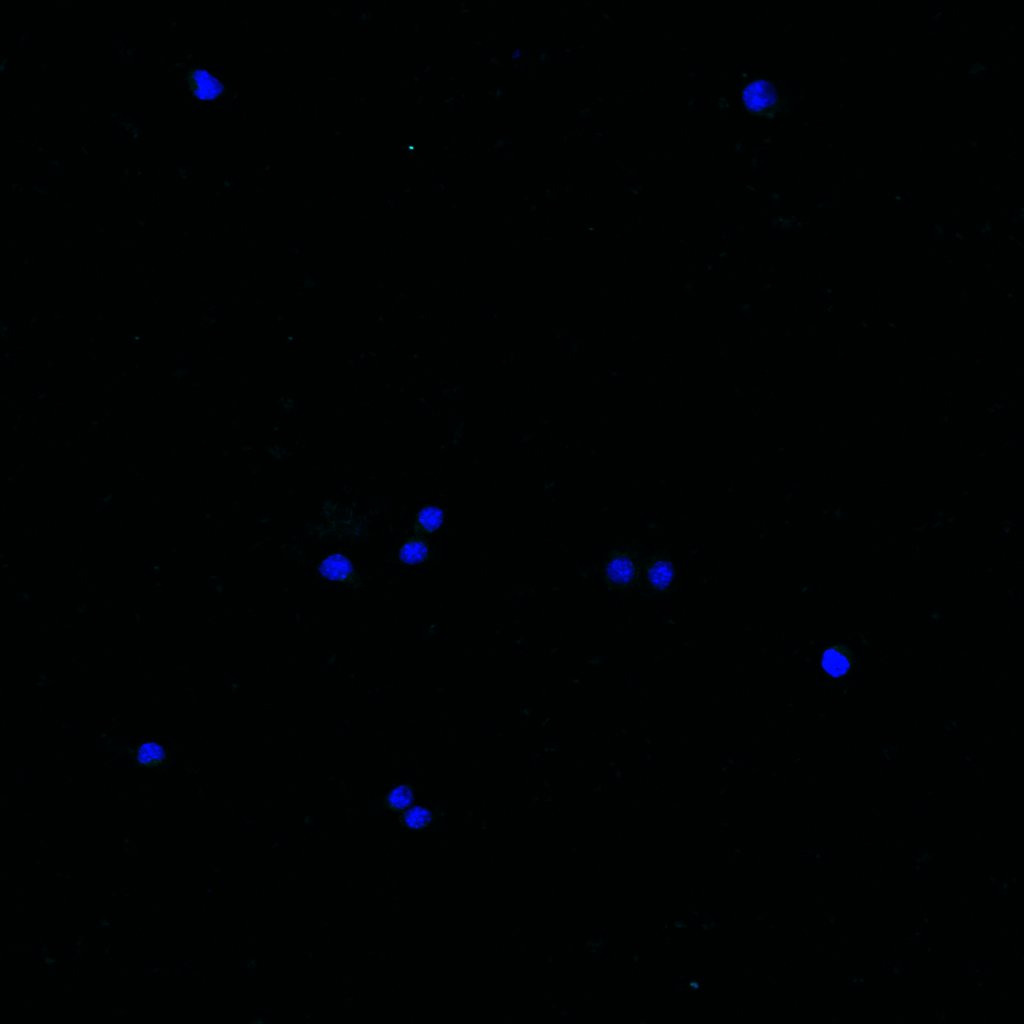

Supplement: Supplementary file 9 — Source data Fig. 4 [file 44318_2024_343_MOESM9_ESM.zip › Figure4/4D/CD133_DAPI,TBR2.jpg]

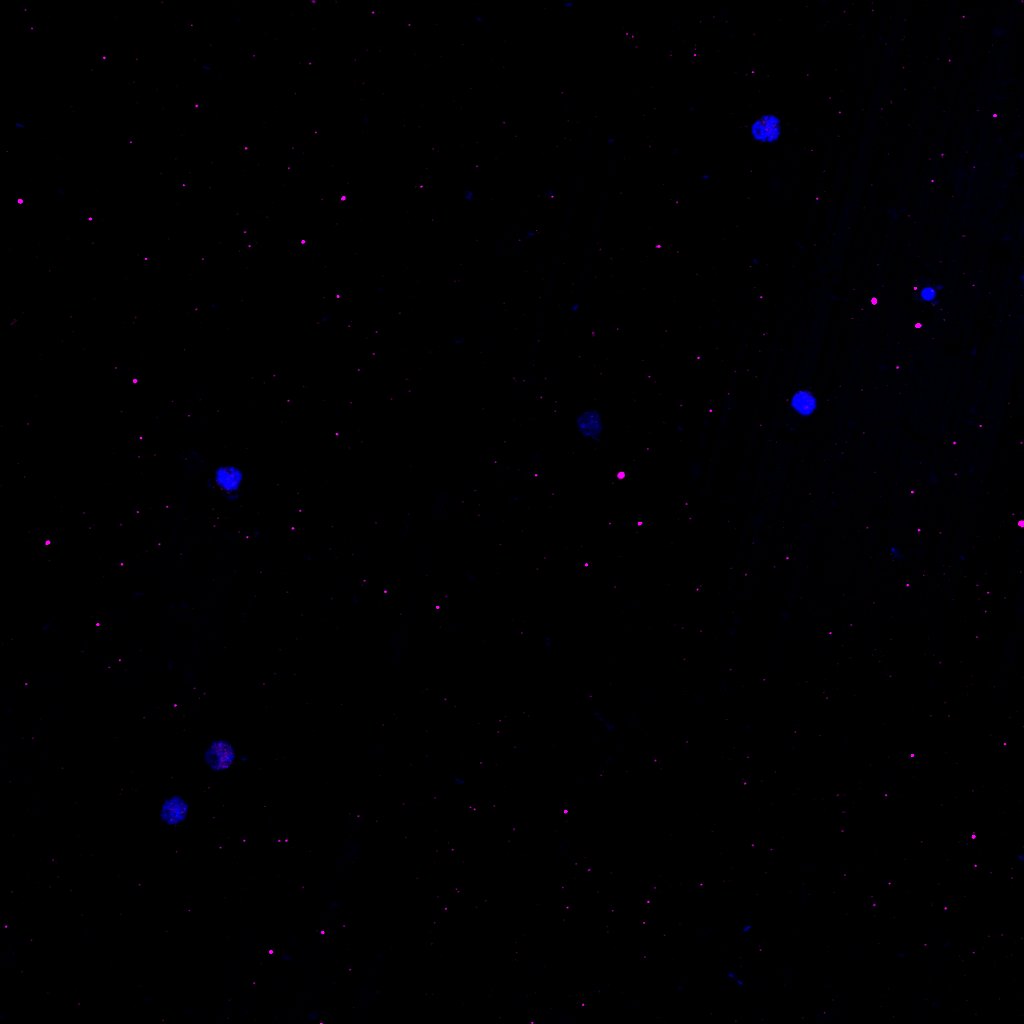

Supplement: Supplementary file 9 — Source data Fig. 4 [file 44318_2024_343_MOESM9_ESM.zip › Figure4/4D/EGFP_DAPI,SOX2.jpg]

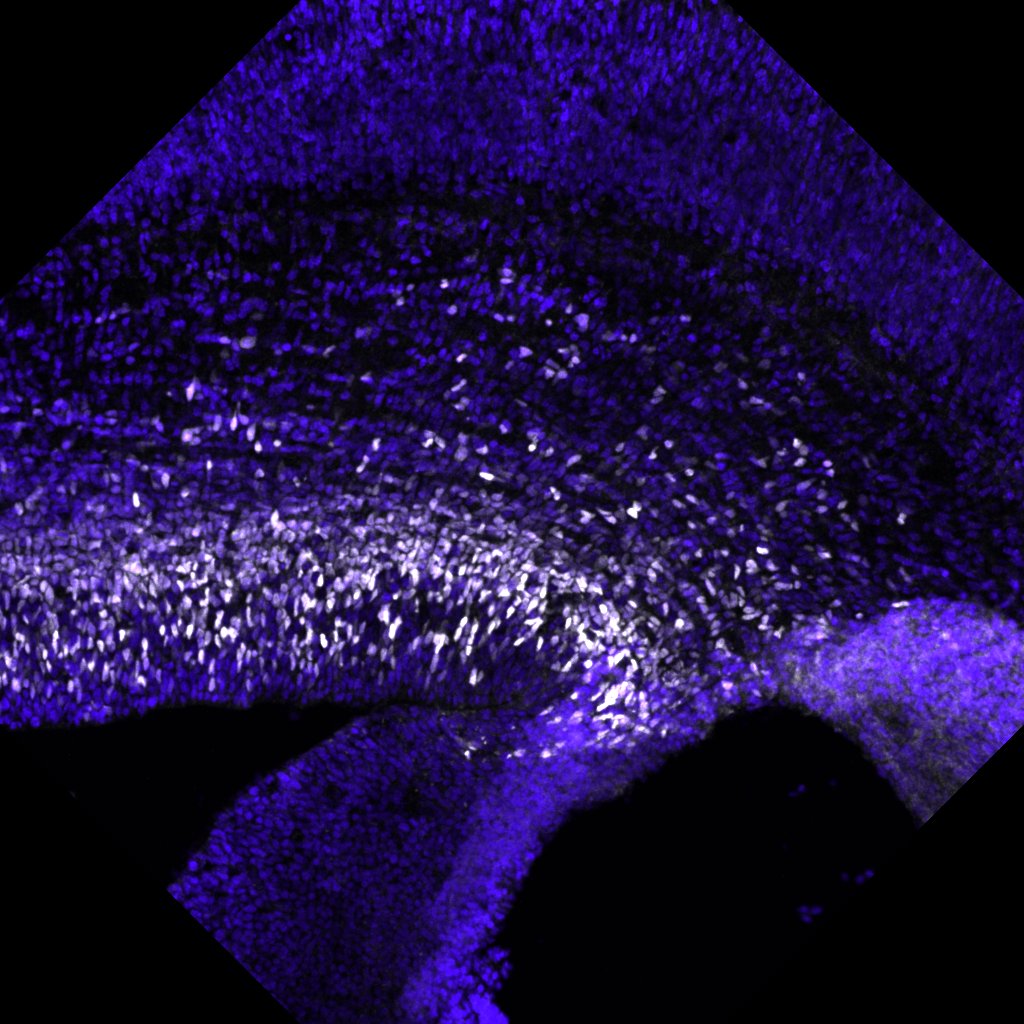

Supplement: Supplementary file 10 — Source data Fig. 5 [file 44318_2024_343_MOESM10_ESM.zip › Figure5/5A/Control_DAPI,TBR2.jpg]

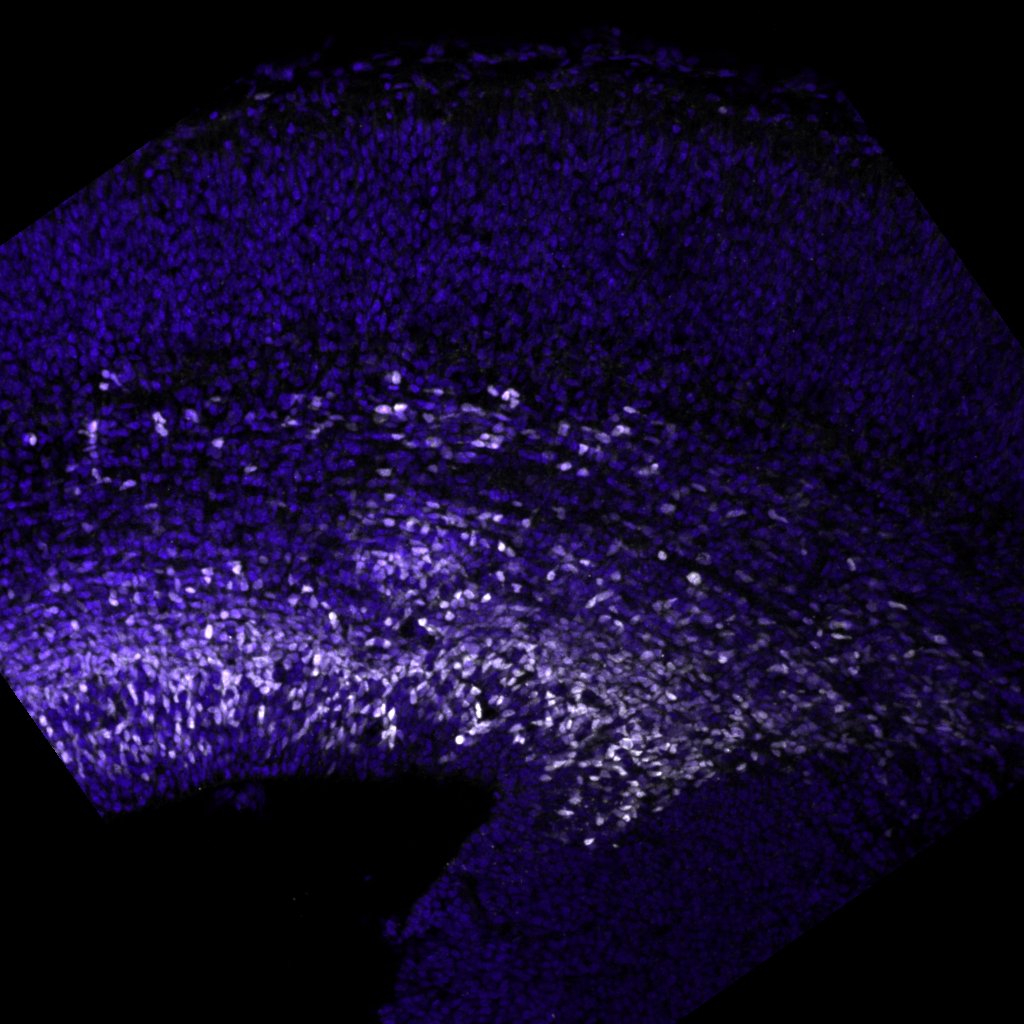

Supplement: Supplementary file 10 — Source data Fig. 5 [file 44318_2024_343_MOESM10_ESM.zip › Figure5/5A/Robo1OE_DAPI,TBR2.jpg]

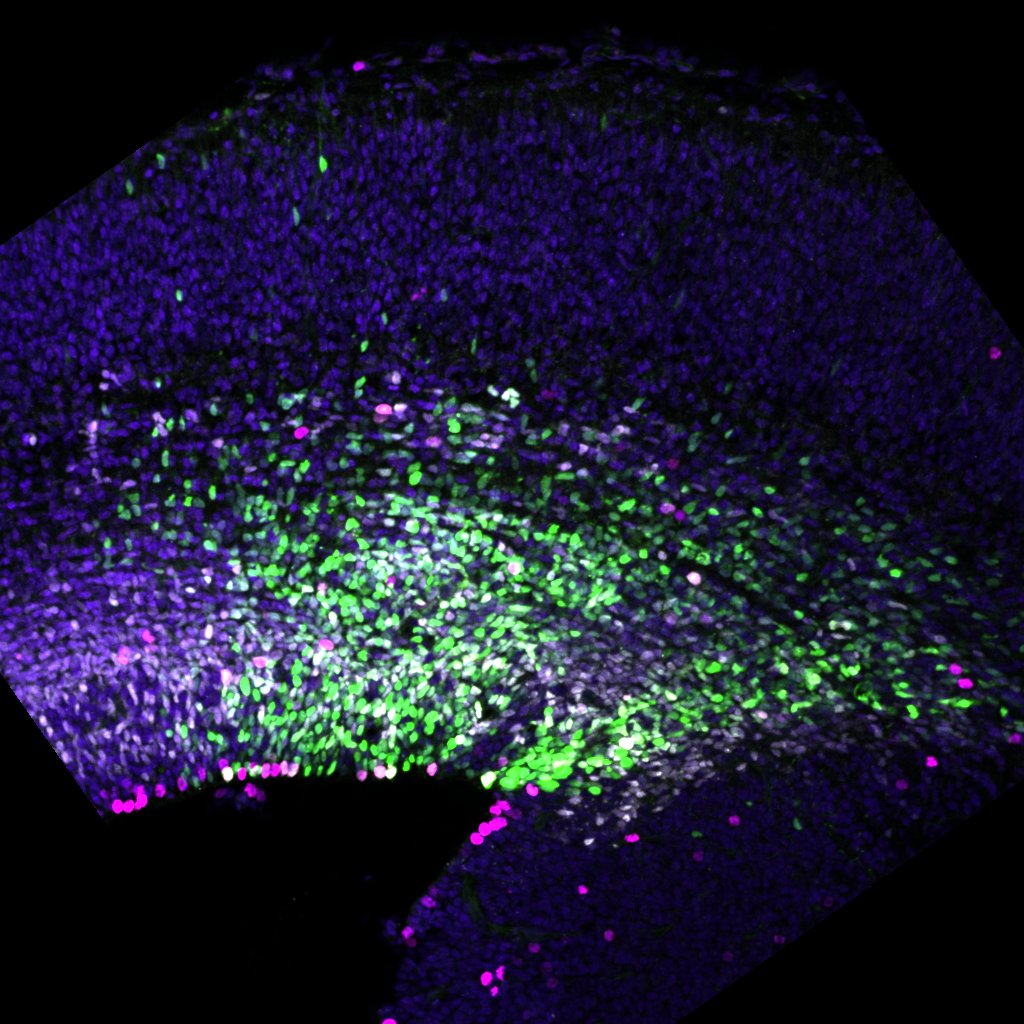

Supplement: Supplementary file 10 — Source data Fig. 5 [file 44318_2024_343_MOESM10_ESM.zip › Figure5/5A/Robo1OE_merge.jpg]

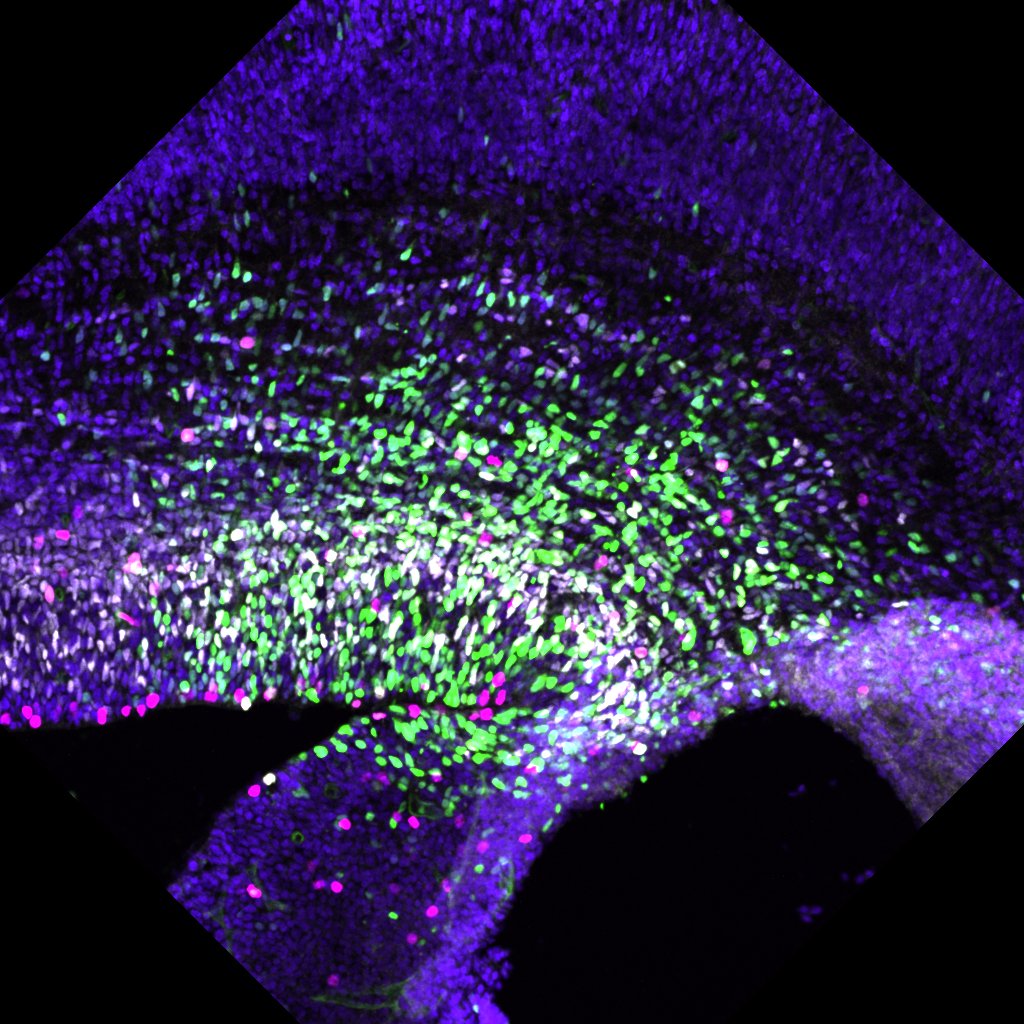

Supplement: Supplementary file 10 — Source data Fig. 5 [file 44318_2024_343_MOESM10_ESM.zip › Figure5/5A/Control_merge.jpg]

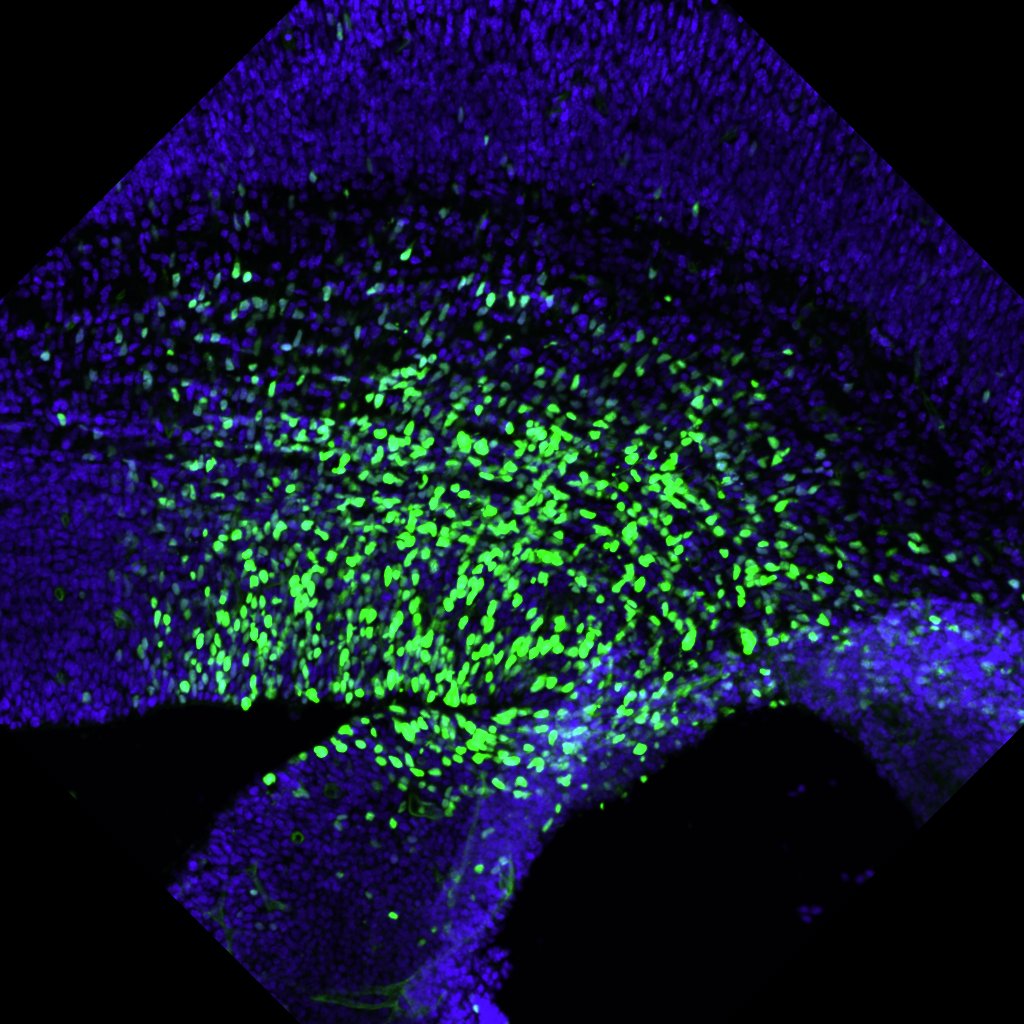

Supplement: Supplementary file 10 — Source data Fig. 5 [file 44318_2024_343_MOESM10_ESM.zip › Figure5/5A/Control_DAPI,GFP.jpg]

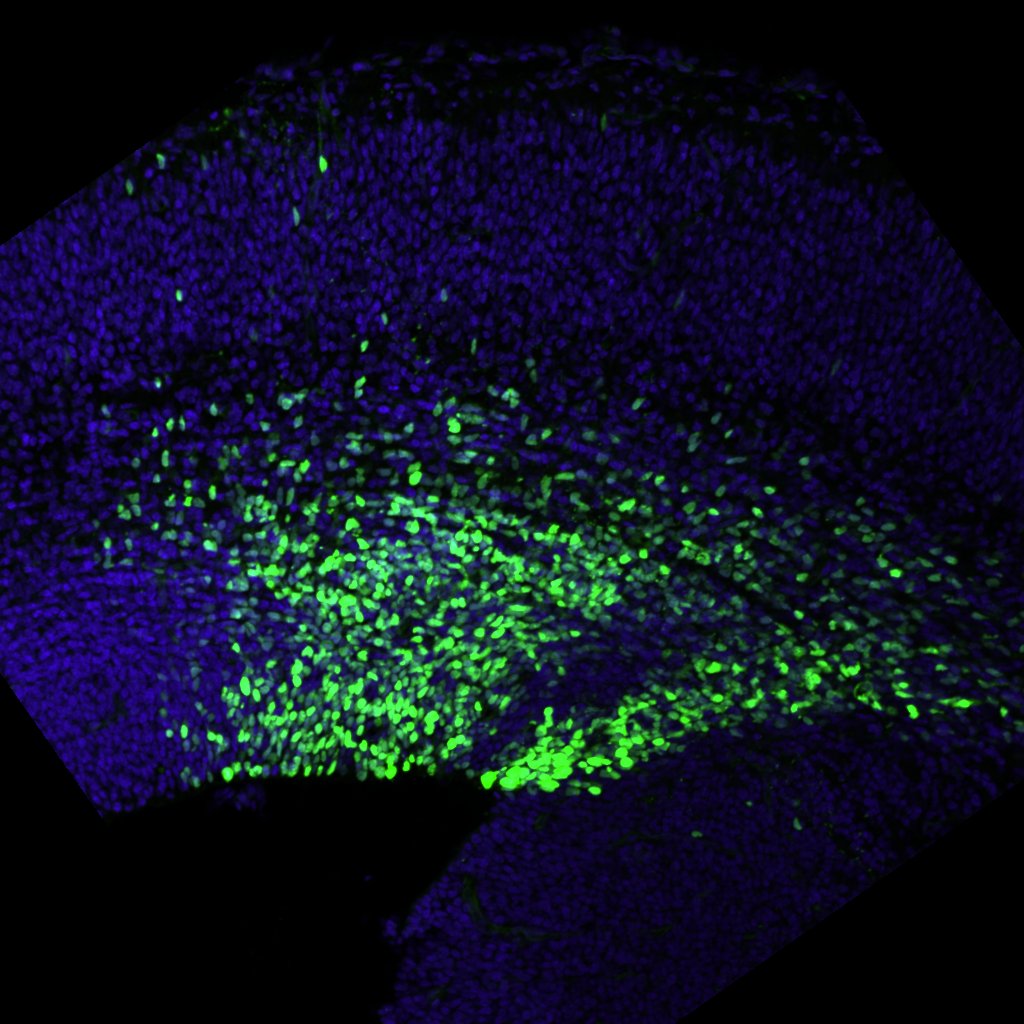

Supplement: Supplementary file 10 — Source data Fig. 5 [file 44318_2024_343_MOESM10_ESM.zip › Figure5/5A/Robo1OE_DAPI,GFP.jpg]

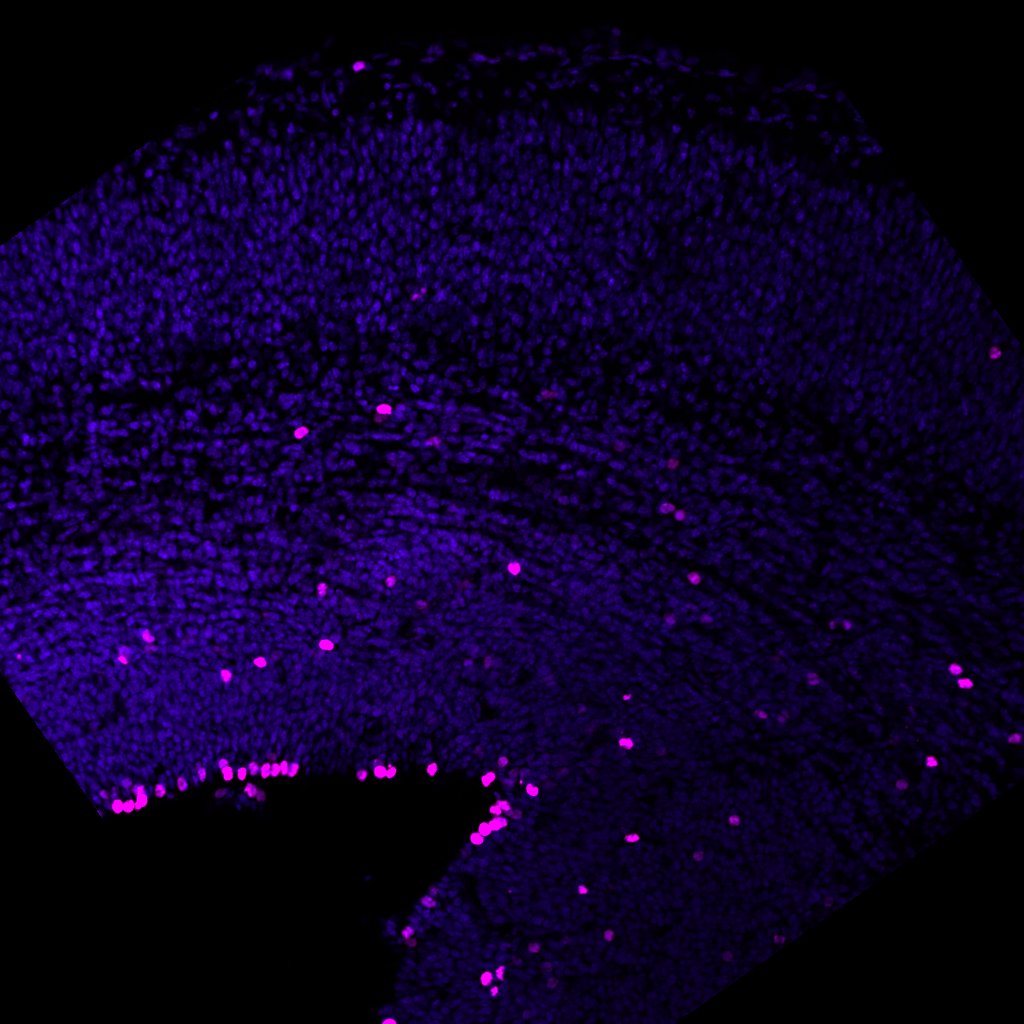

Supplement: Supplementary file 10 — Source data Fig. 5 [file 44318_2024_343_MOESM10_ESM.zip › Figure5/5A/Robo1OE_DAPI,PH3.jpg]

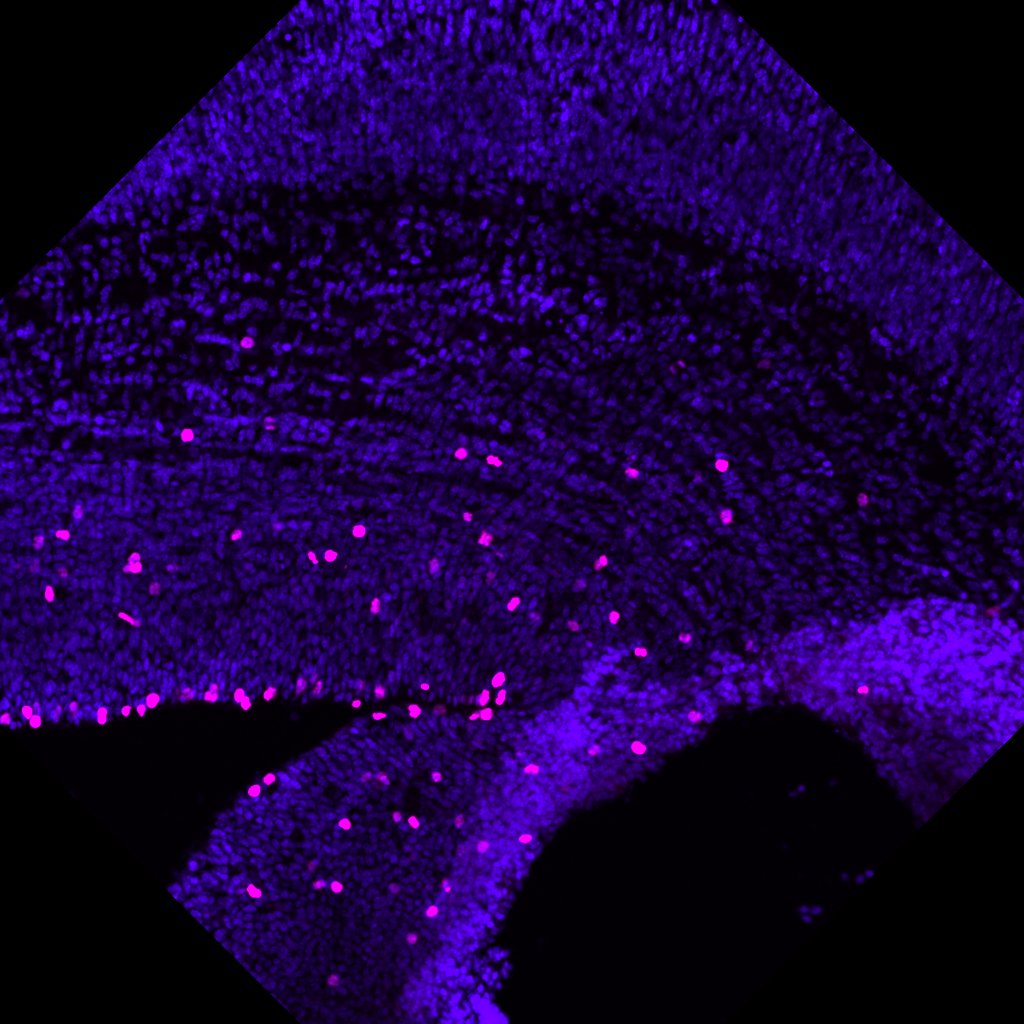

Supplement: Supplementary file 10 — Source data Fig. 5 [file 44318_2024_343_MOESM10_ESM.zip › Figure5/5A/Control_DAPI,PH3.jpg]

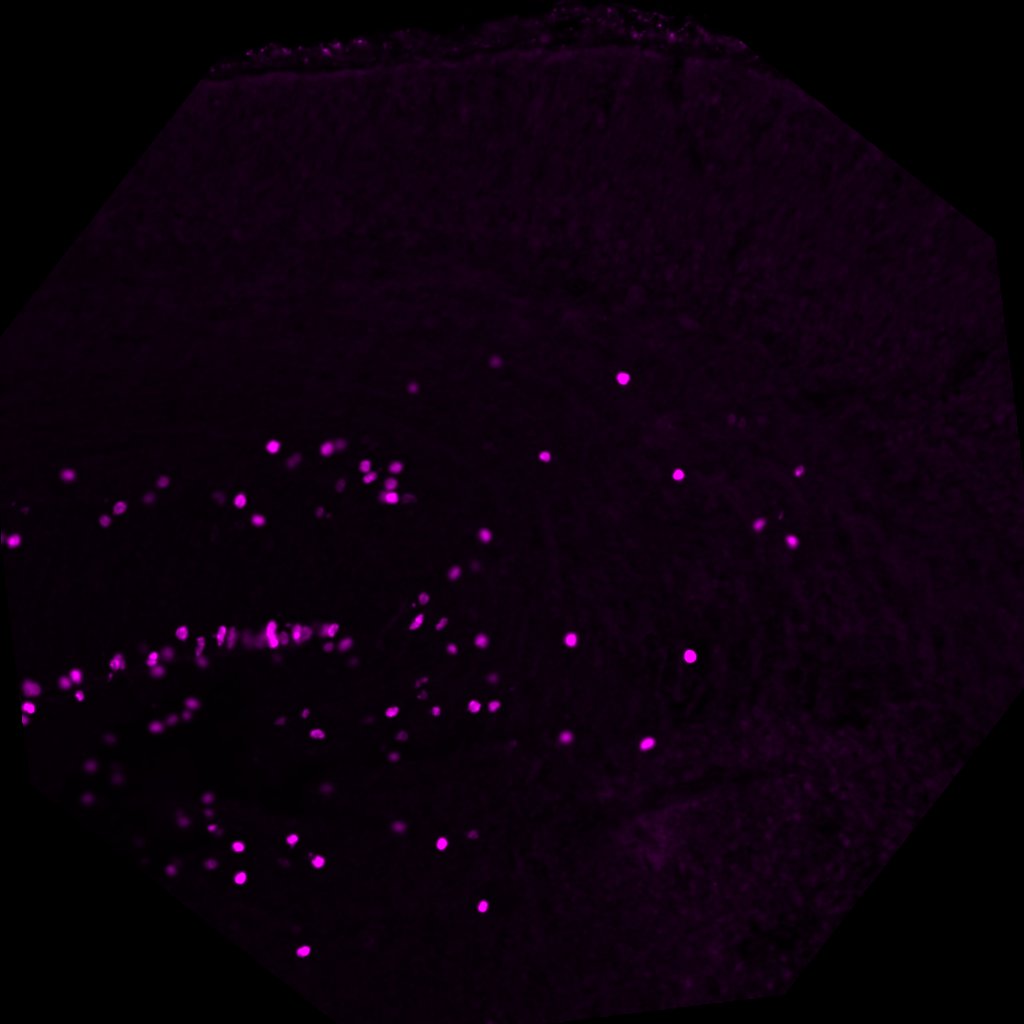

Supplement: Supplementary file 10 — Source data Fig. 5 [file 44318_2024_343_MOESM10_ESM.zip › Figure5/5C/Auts2cKO+Robo1sh2_PH3.jpg]

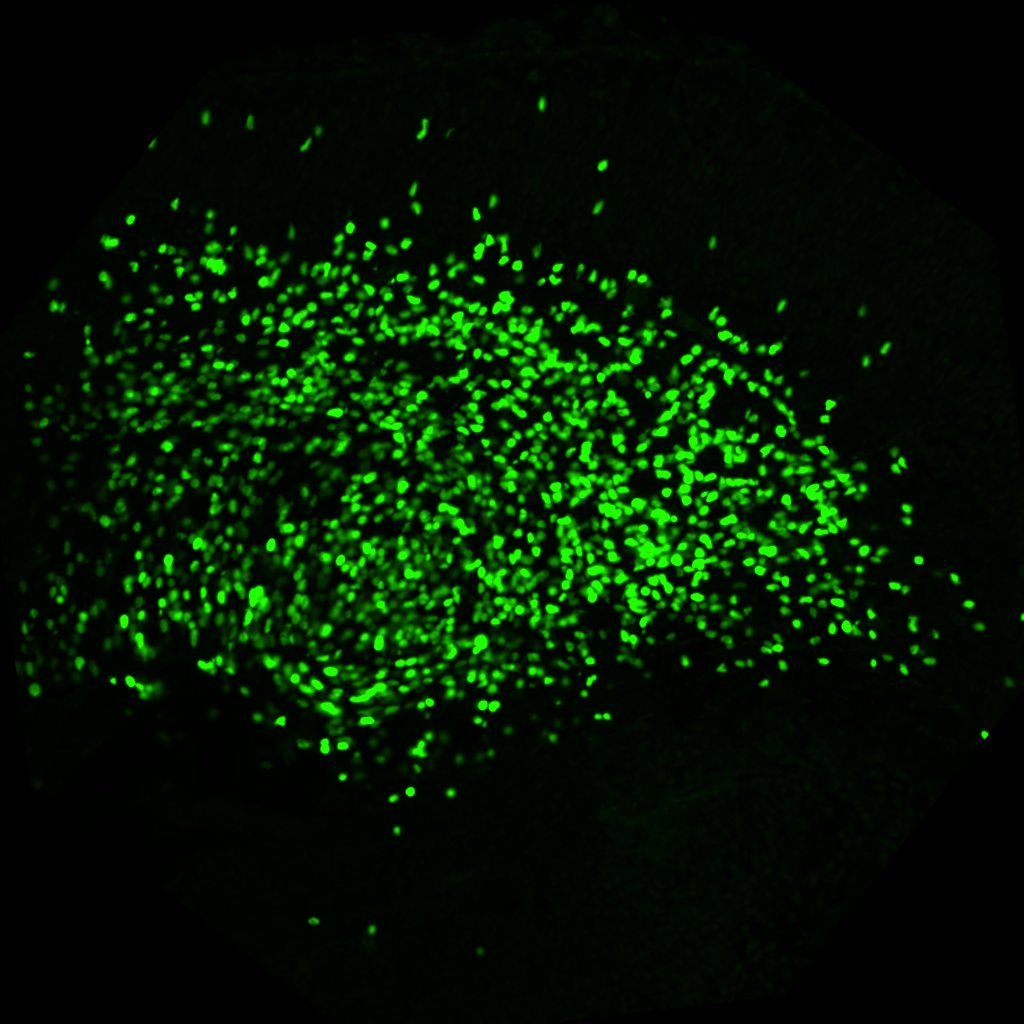

Supplement: Supplementary file 10 — Source data Fig. 5 [file 44318_2024_343_MOESM10_ESM.zip › Figure5/5C/Auts2cKO+Robo1sh2_GFP.jpg]

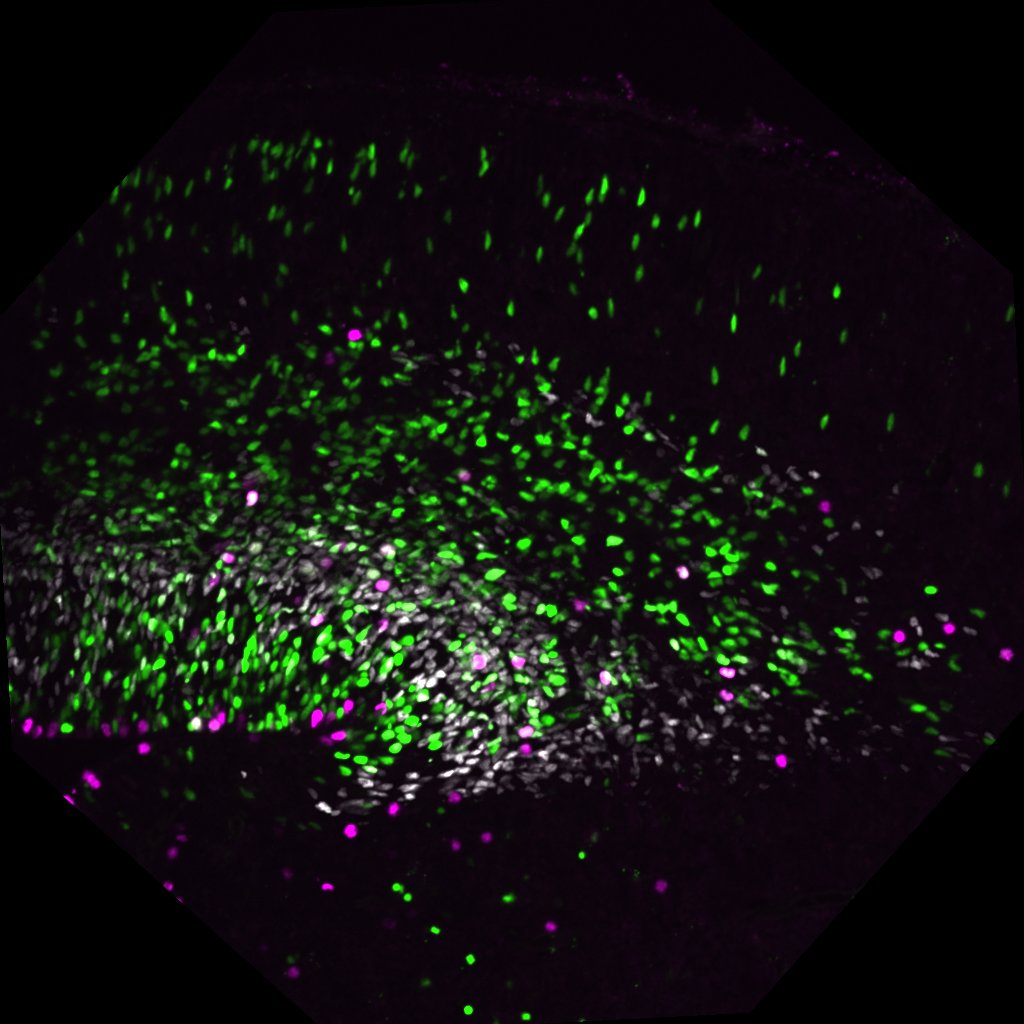

Supplement: Supplementary file 10 — Source data Fig. 5 [file 44318_2024_343_MOESM10_ESM.zip › Figure5/5C/Auts2fl:fl+shScramble_merge.jpg]

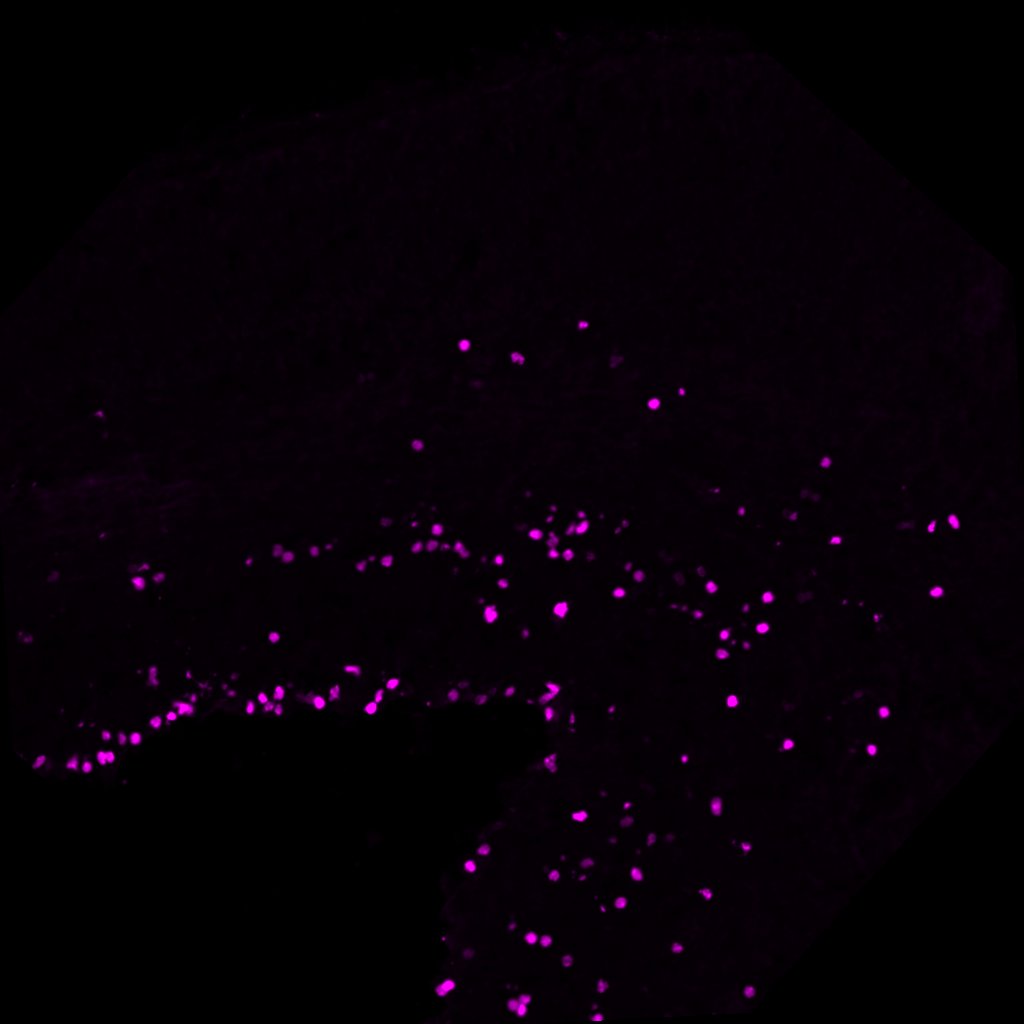

Supplement: Supplementary file 10 — Source data Fig. 5 [file 44318_2024_343_MOESM10_ESM.zip › Figure5/5C/Auts2cKO+Robo1sh1_PH3.jpg]

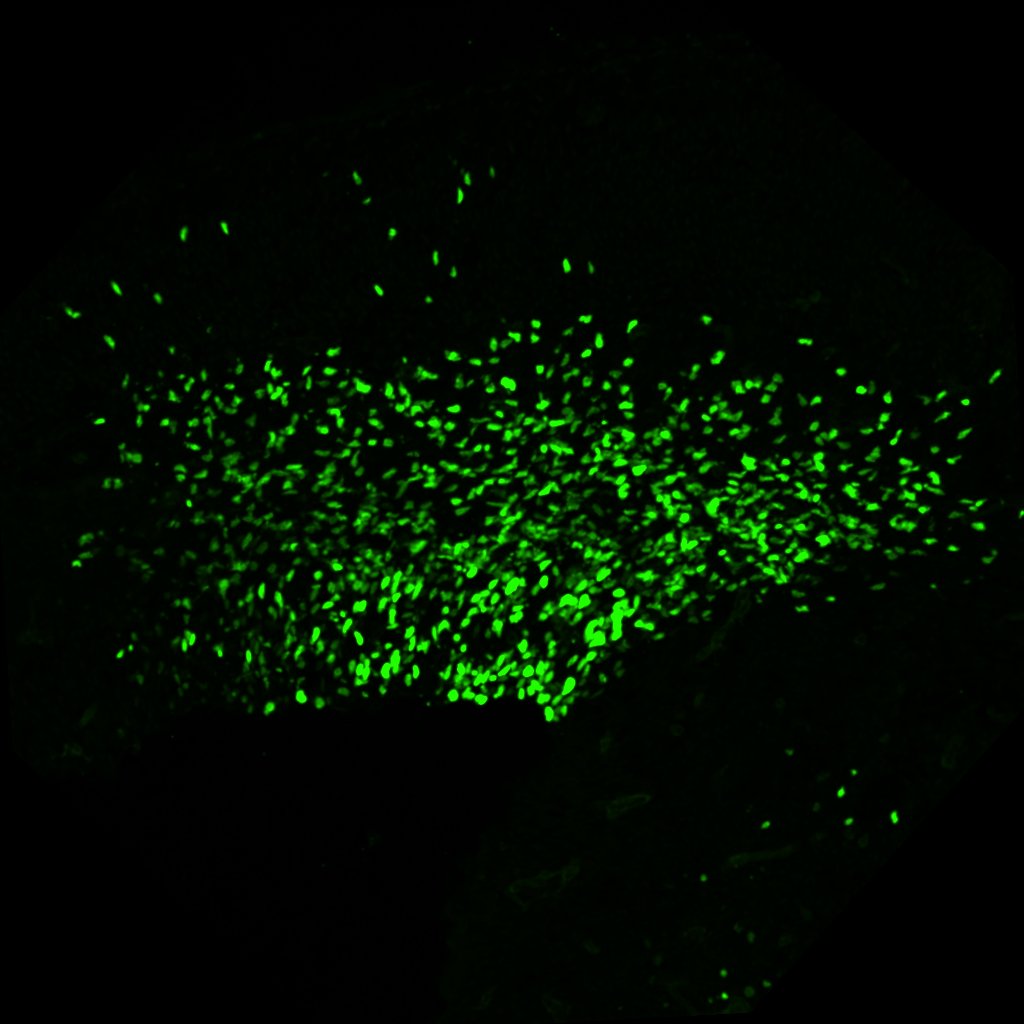

Supplement: Supplementary file 10 — Source data Fig. 5 [file 44318_2024_343_MOESM10_ESM.zip › Figure5/5C/Auts2cKO+Robo1sh1_GFP.jpg]

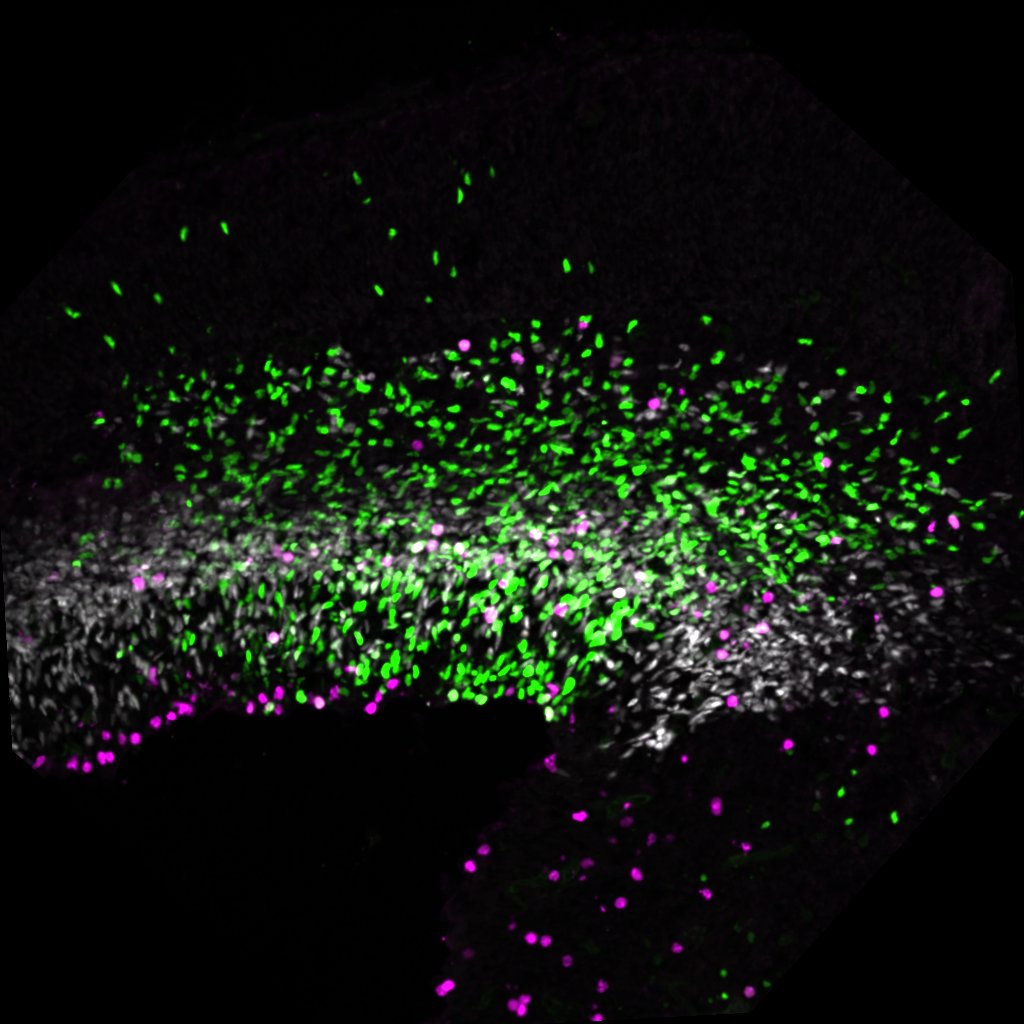

Supplement: Supplementary file 10 — Source data Fig. 5 [file 44318_2024_343_MOESM10_ESM.zip › Figure5/5C/Auts2cKO+Robo1sh1_merge.jpg]

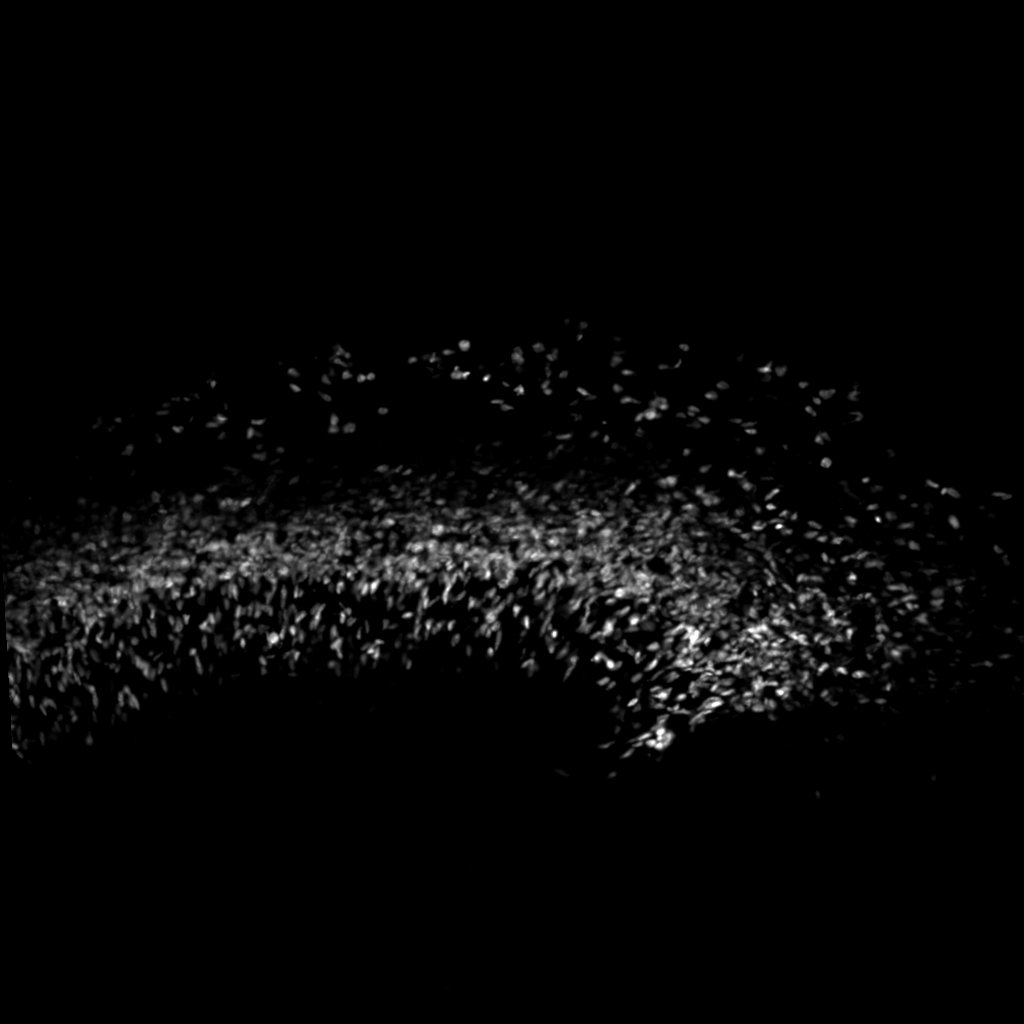

Supplement: Supplementary file 10 — Source data Fig. 5 [file 44318_2024_343_MOESM10_ESM.zip › Figure5/5C/Auts2cKO+Robo1sh1_TBR2.jpg]

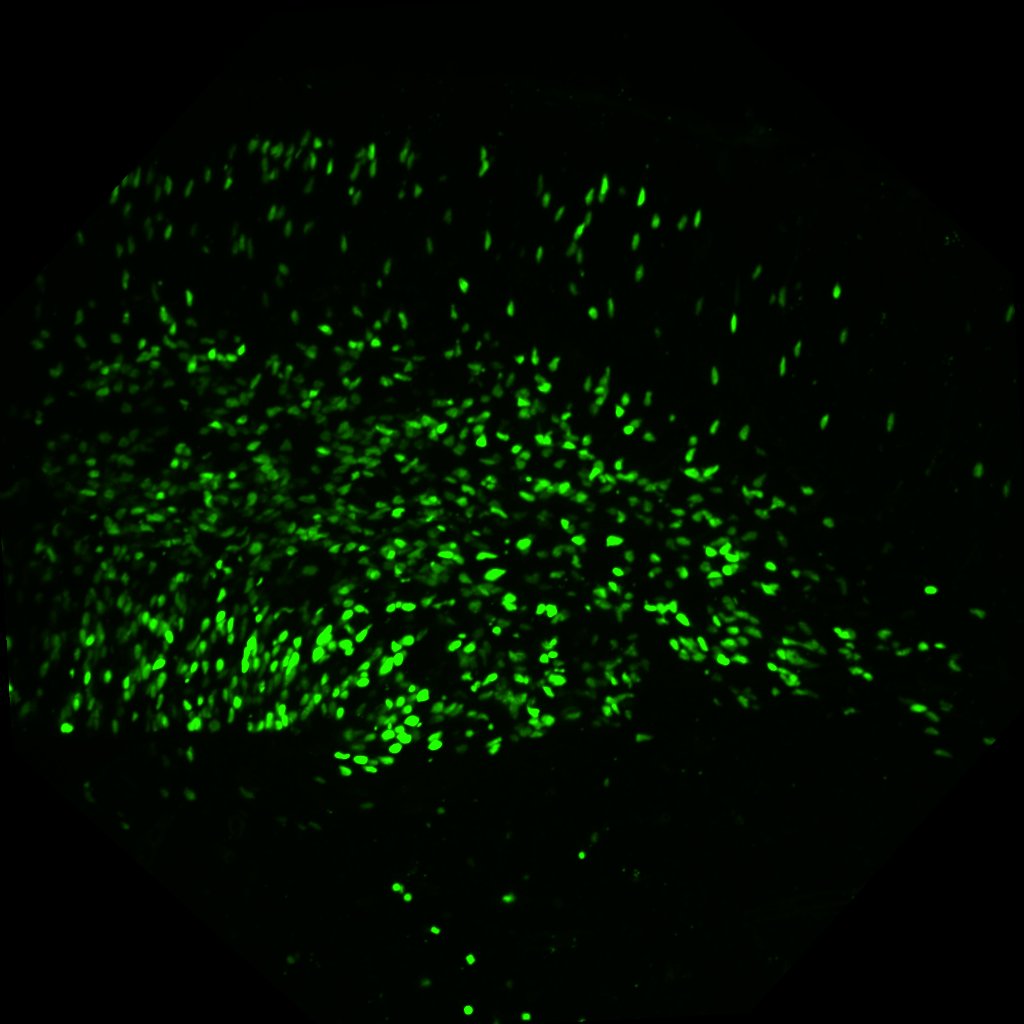

Supplement: Supplementary file 10 — Source data Fig. 5 [file 44318_2024_343_MOESM10_ESM.zip › Figure5/5C/Auts2fl:fl+shScramble_GFP.jpg]

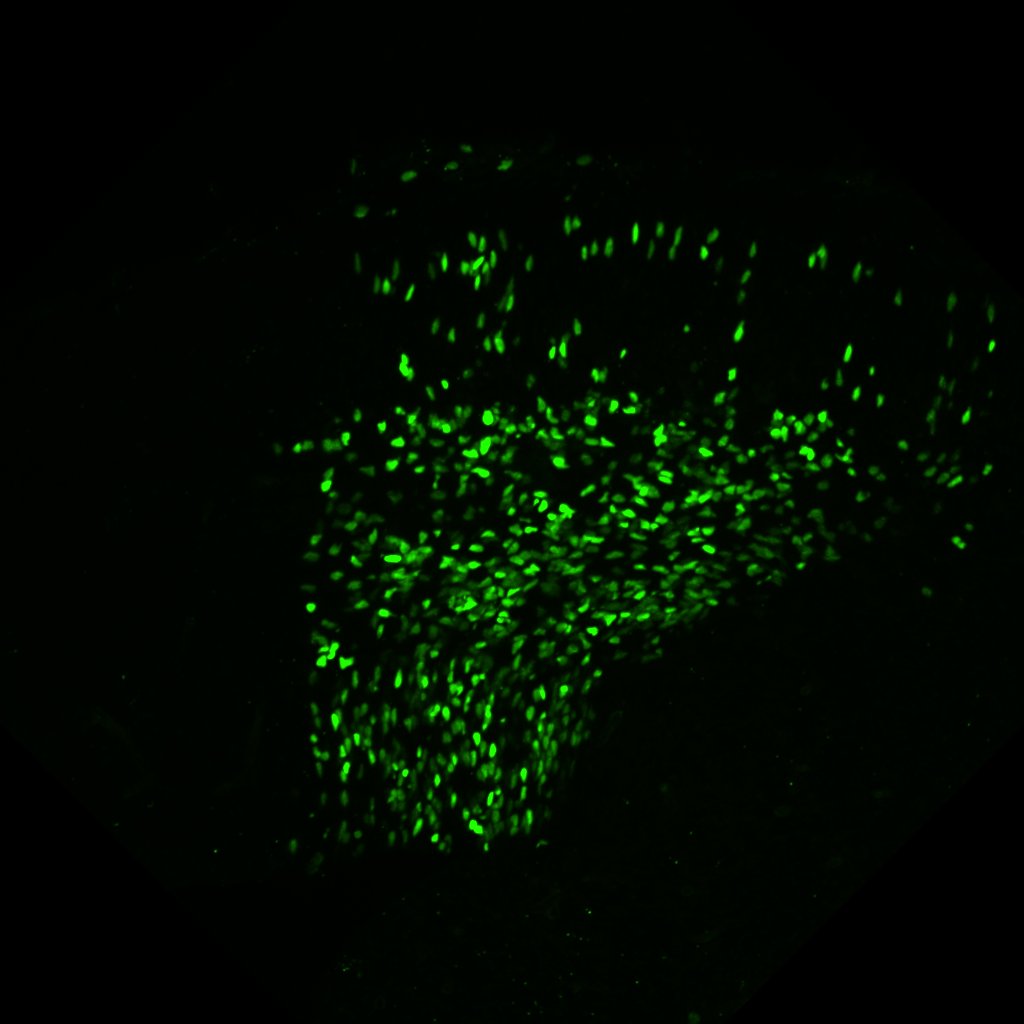

Supplement: Supplementary file 10 — Source data Fig. 5 [file 44318_2024_343_MOESM10_ESM.zip › Figure5/5C/Auts2cKO+shScramble_GFP.jpg]

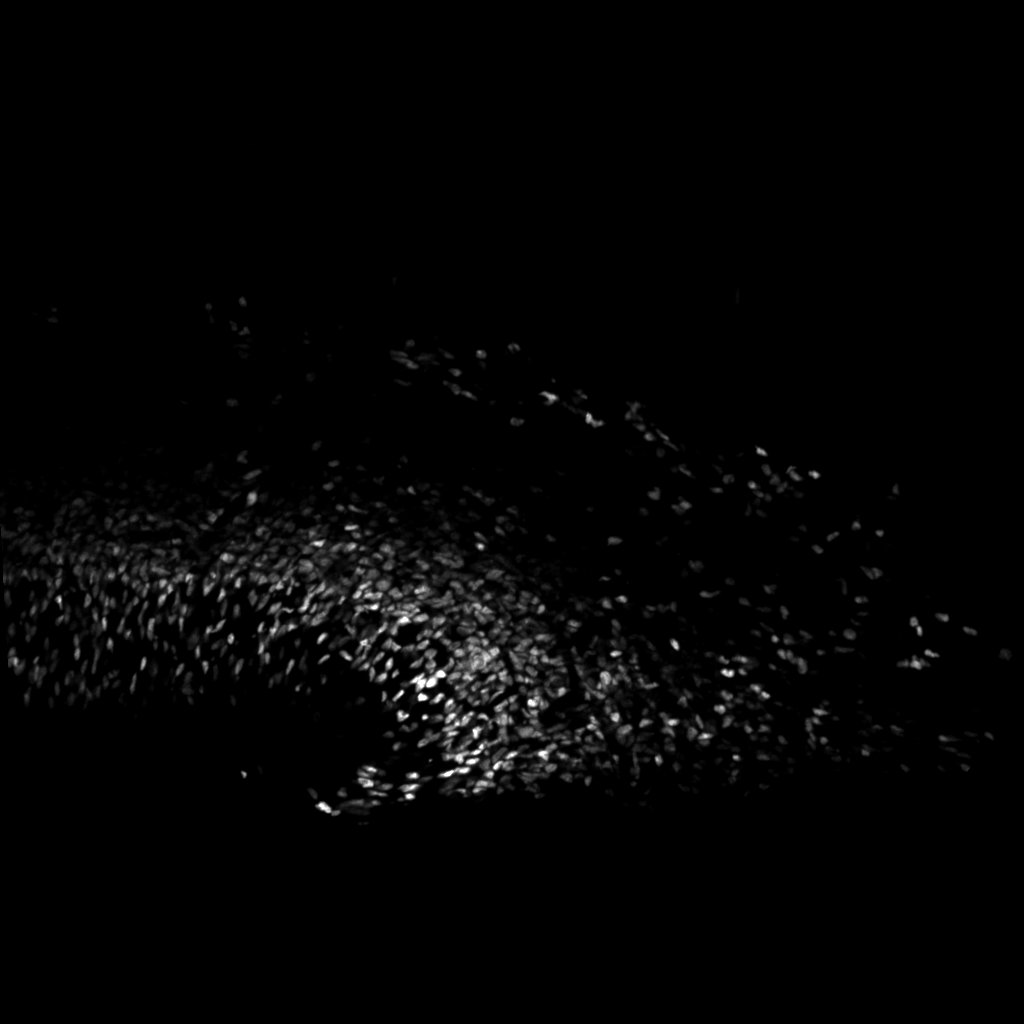

Supplement: Supplementary file 10 — Source data Fig. 5 [file 44318_2024_343_MOESM10_ESM.zip › Figure5/5C/Auts2fl:fl+shScramble_TBR2.jpg]

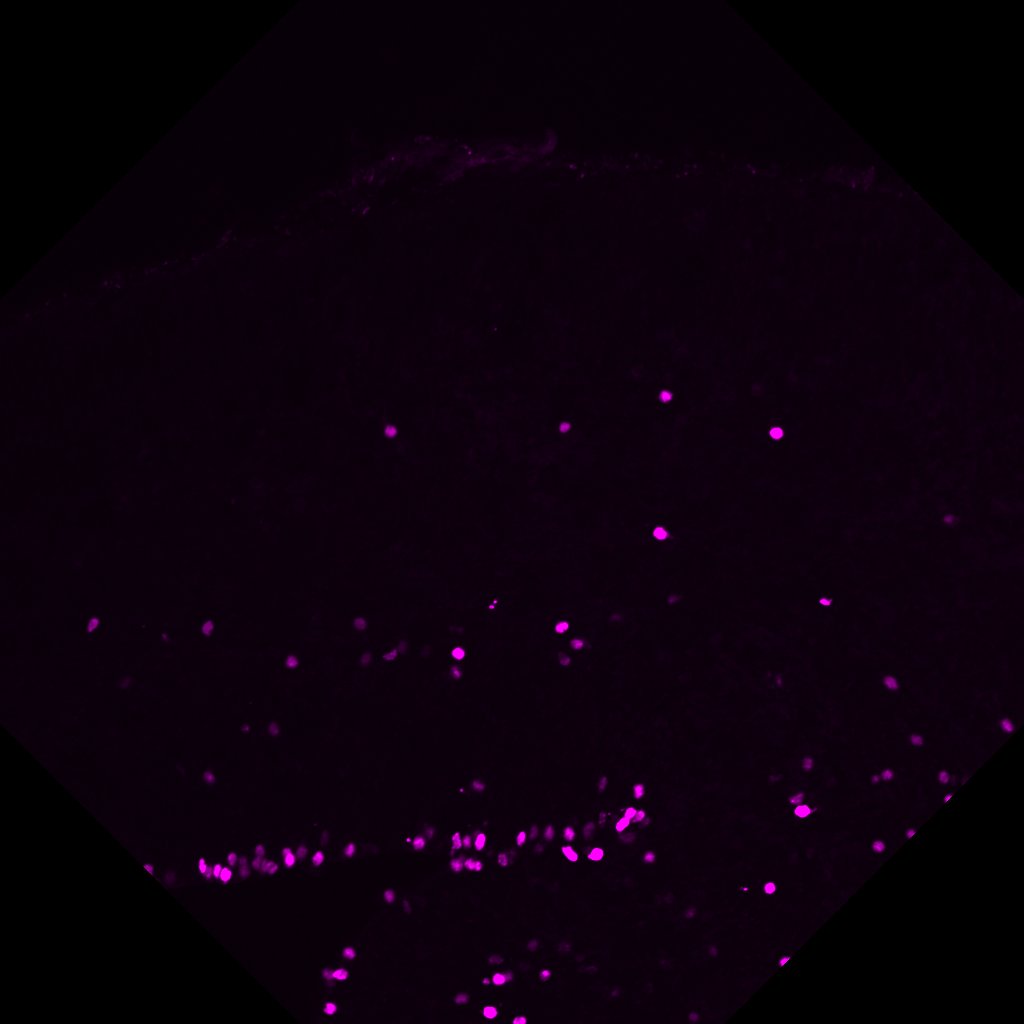

Supplement: Supplementary file 10 — Source data Fig. 5 [file 44318_2024_343_MOESM10_ESM.zip › Figure5/5C/Auts2cKO+shScramble_PH3.jpg]

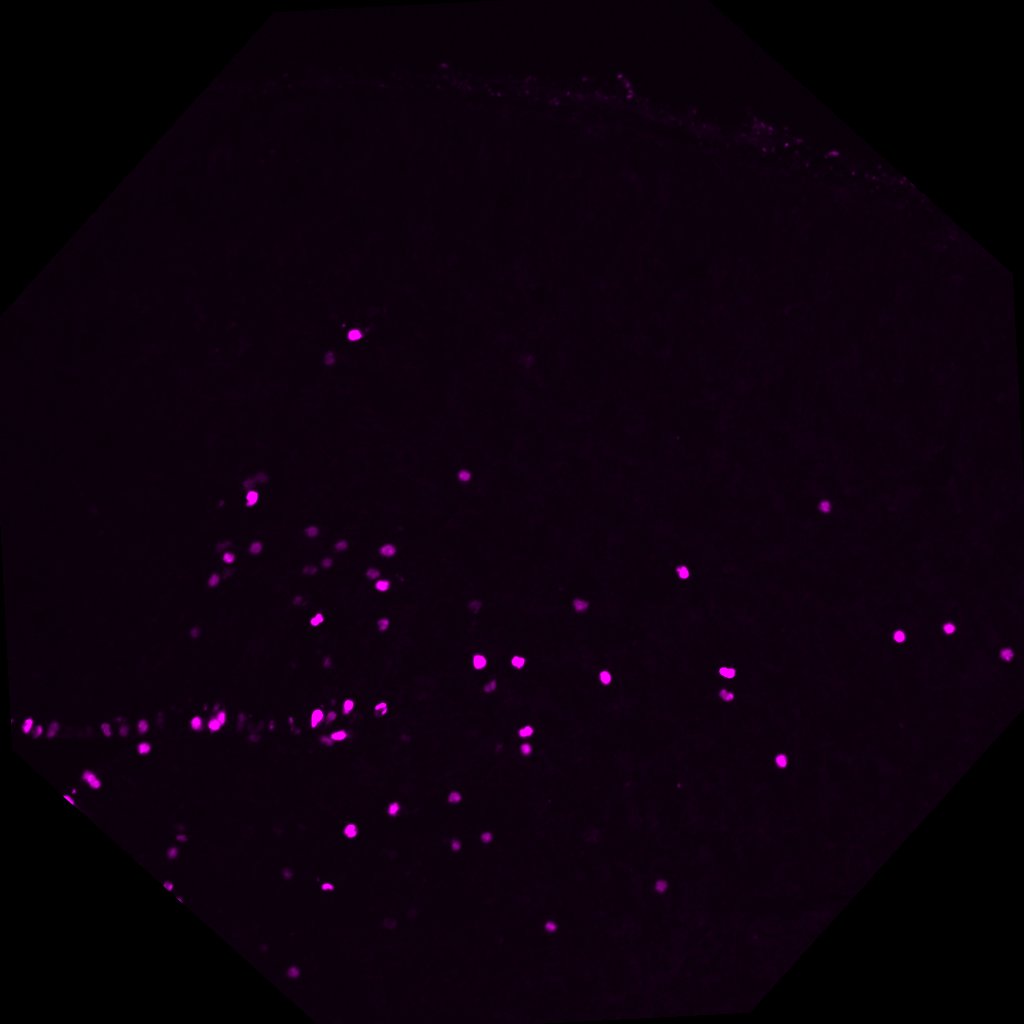

Supplement: Supplementary file 10 — Source data Fig. 5 [file 44318_2024_343_MOESM10_ESM.zip › Figure5/5C/Auts2fl:fl+shScramble_PH3.jpg]

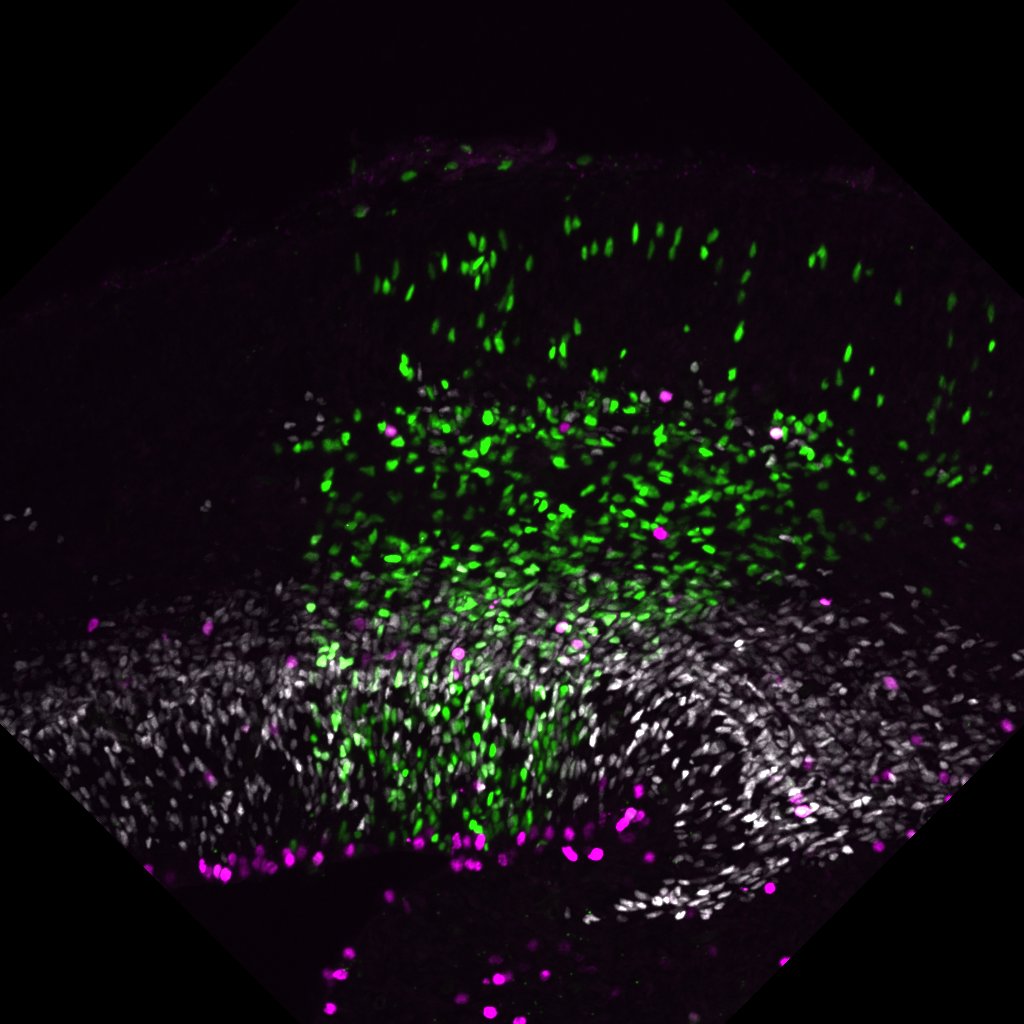

Supplement: Supplementary file 10 — Source data Fig. 5 [file 44318_2024_343_MOESM10_ESM.zip › Figure5/5C/Auts2cKO+shScramble_merge.jpg]

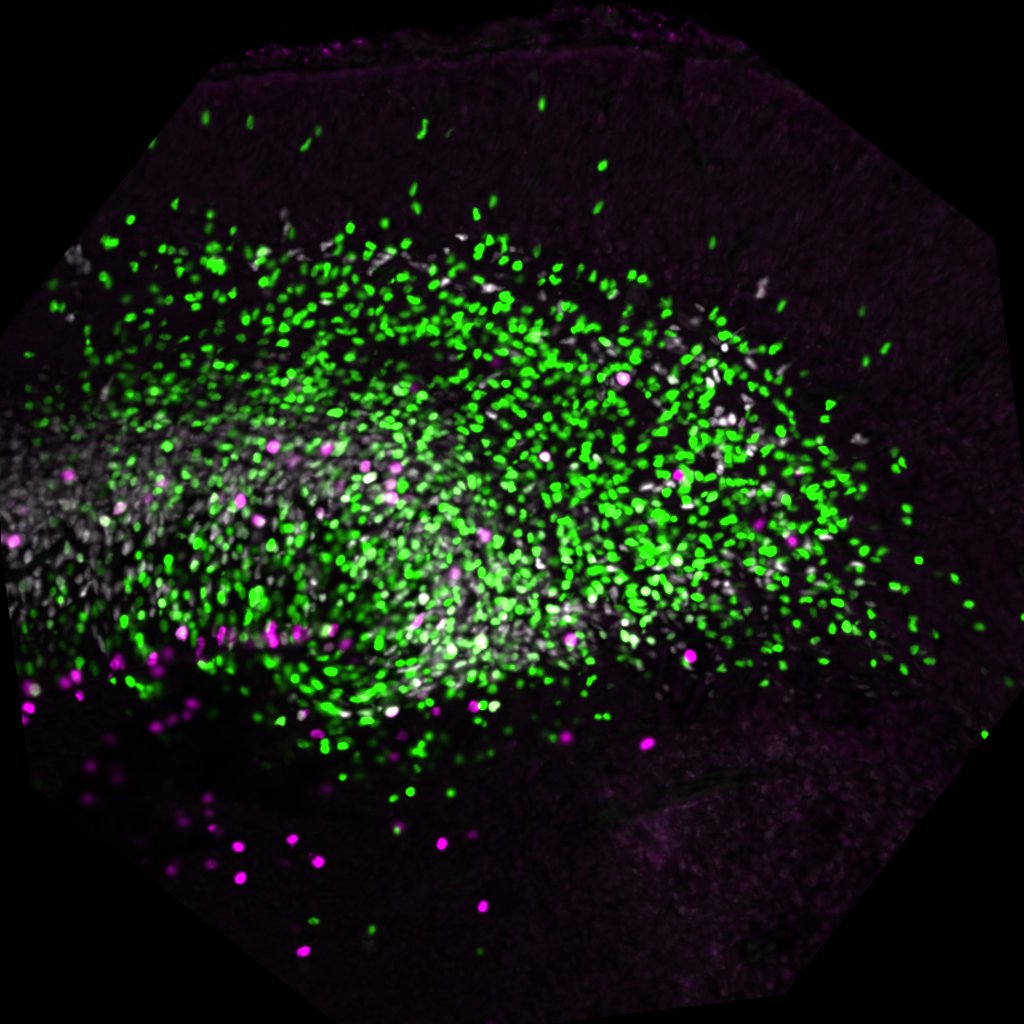

Supplement: Supplementary file 10 — Source data Fig. 5 [file 44318_2024_343_MOESM10_ESM.zip › Figure5/5C/Auts2cKO+Robo1sh2_merge.jpg]

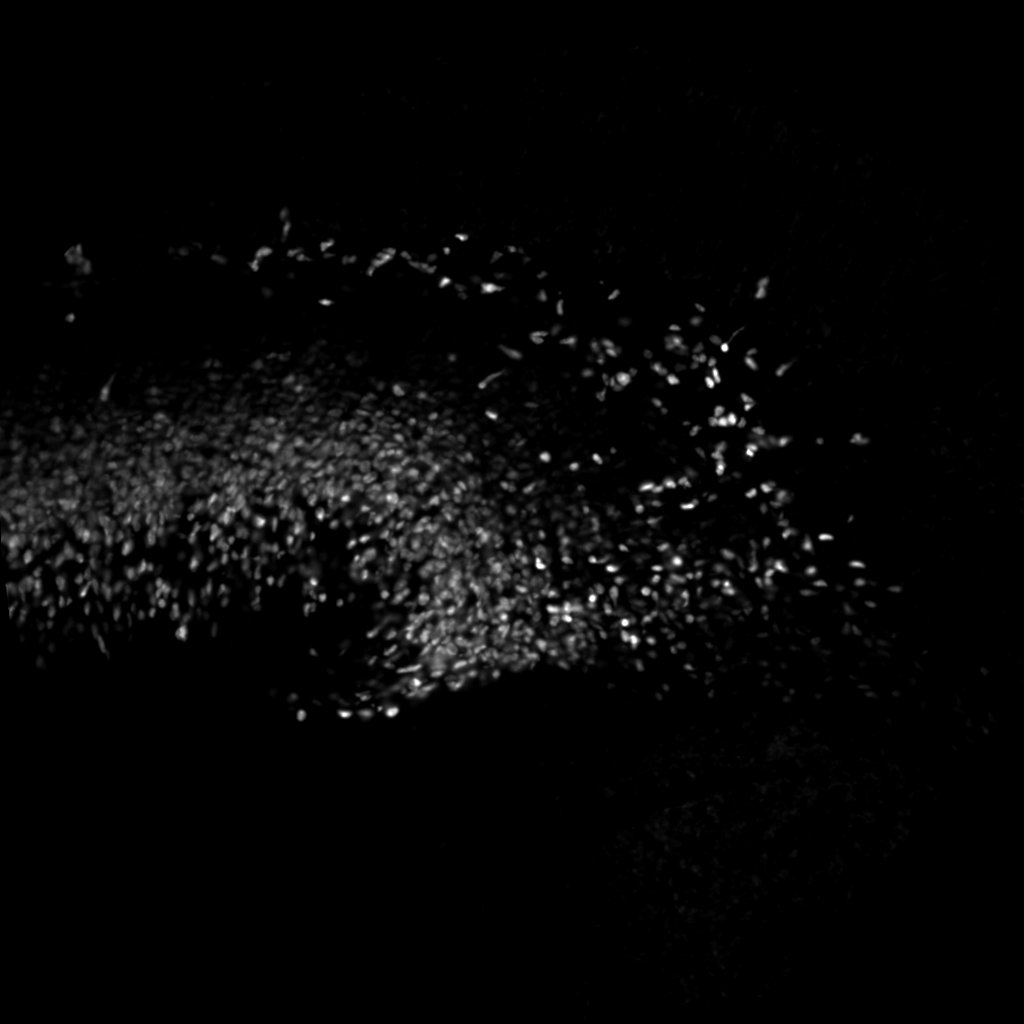

Supplement: Supplementary file 10 — Source data Fig. 5 [file 44318_2024_343_MOESM10_ESM.zip › Figure5/5C/Auts2cKO+Robo1sh2_TBR2.jpg]

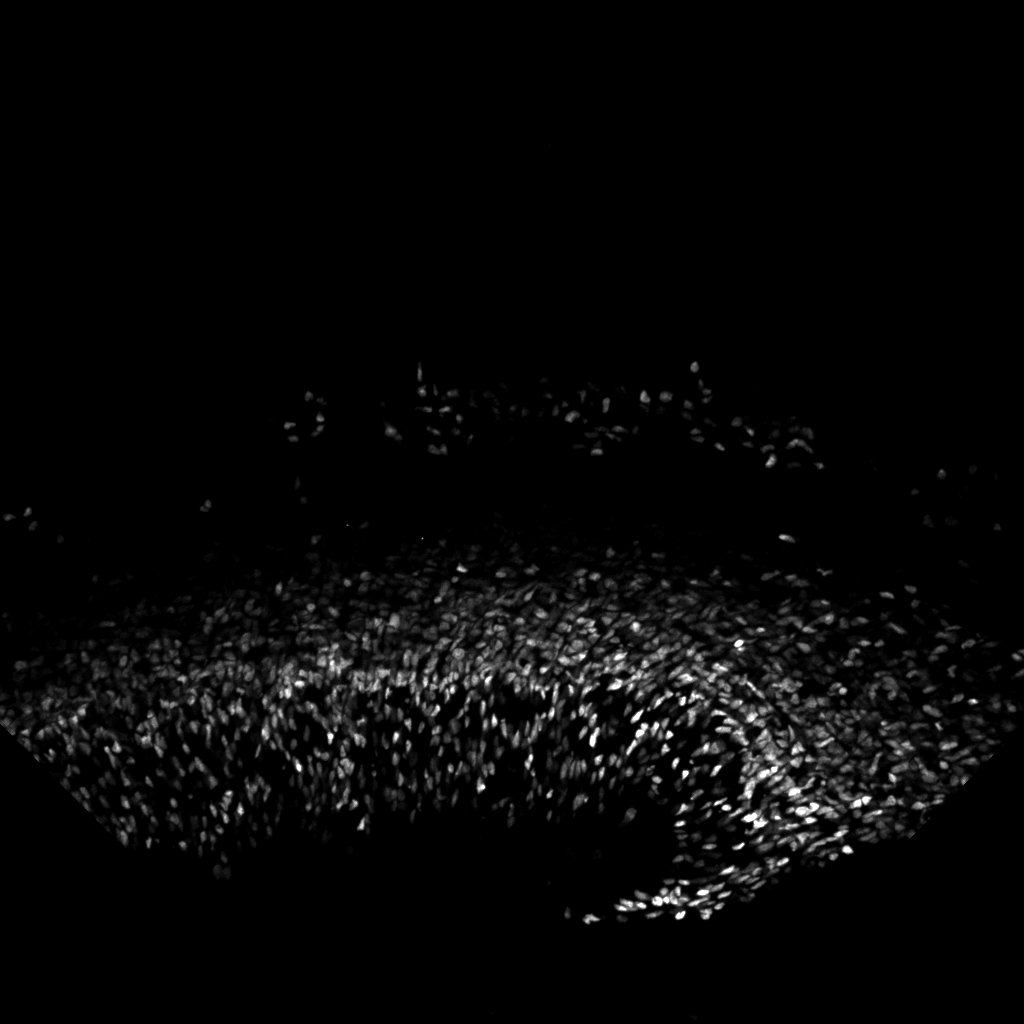

Supplement: Supplementary file 10 — Source data Fig. 5 [file 44318_2024_343_MOESM10_ESM.zip › Figure5/5C/Auts2cKO+shScramble_TBR2.jpg]

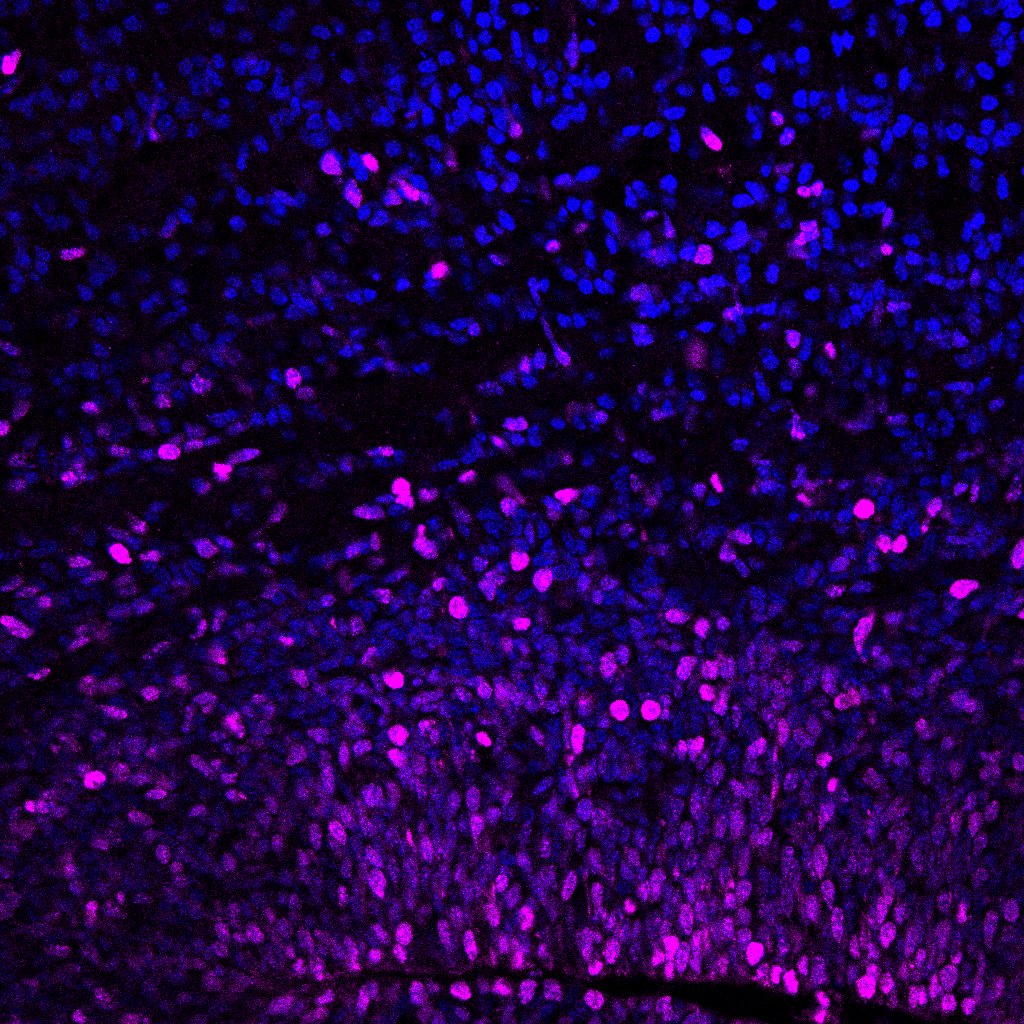

Supplement: Supplementary file 10 — Source data Fig. 5 [file 44318_2024_343_MOESM10_ESM.zip › Figure5/5B/Robo1OE_DAPI,KI67.jpg]

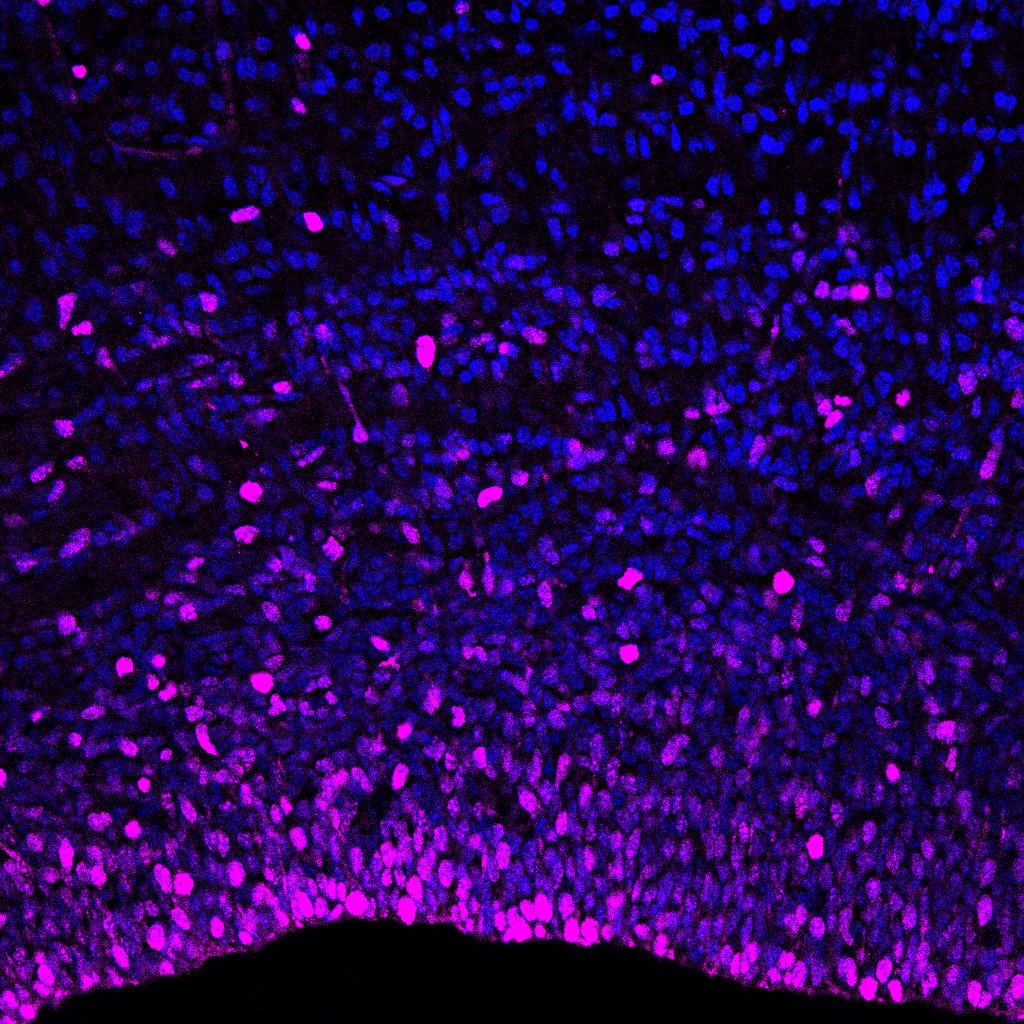

Supplement: Supplementary file 10 — Source data Fig. 5 [file 44318_2024_343_MOESM10_ESM.zip › Figure5/5B/Control_DAPI,KI67.jpg]

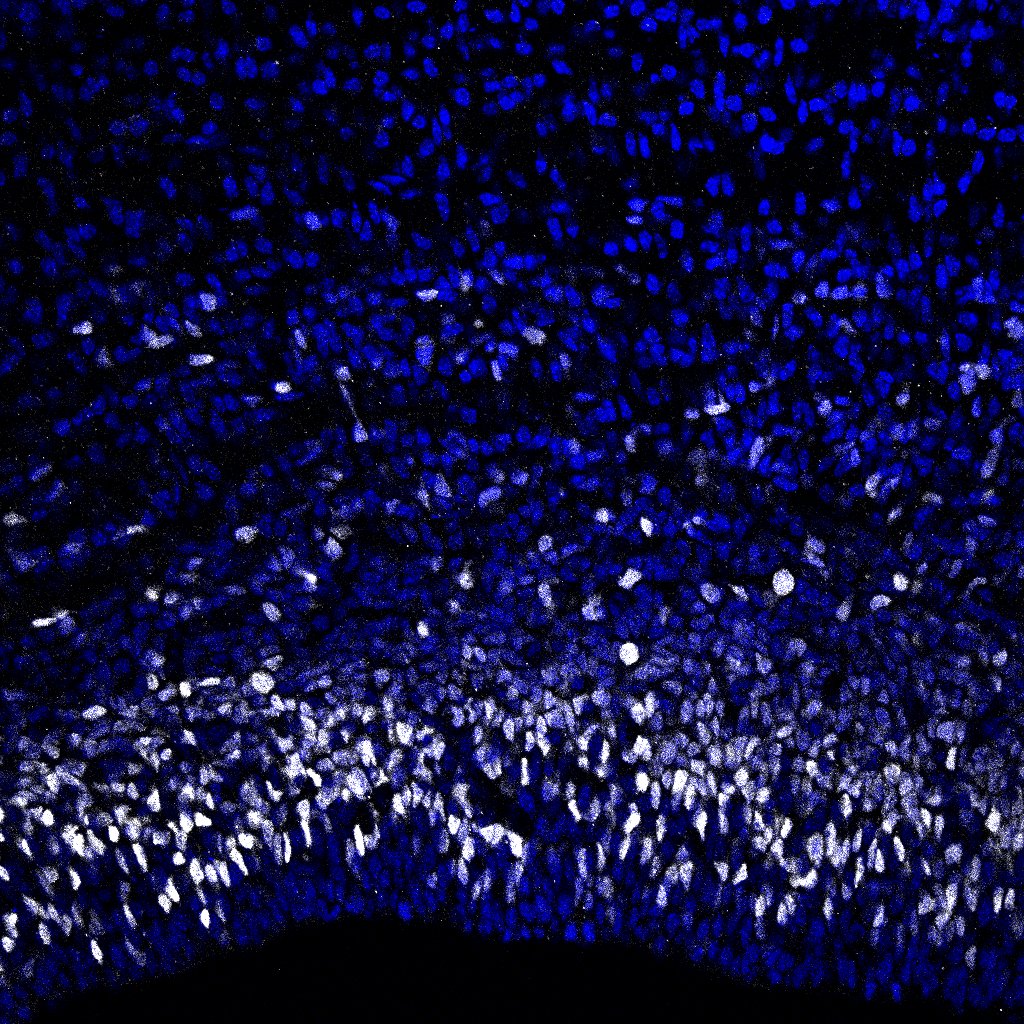

Supplement: Supplementary file 10 — Source data Fig. 5 [file 44318_2024_343_MOESM10_ESM.zip › Figure5/5B/Control_DAPI,TBR2.jpg]

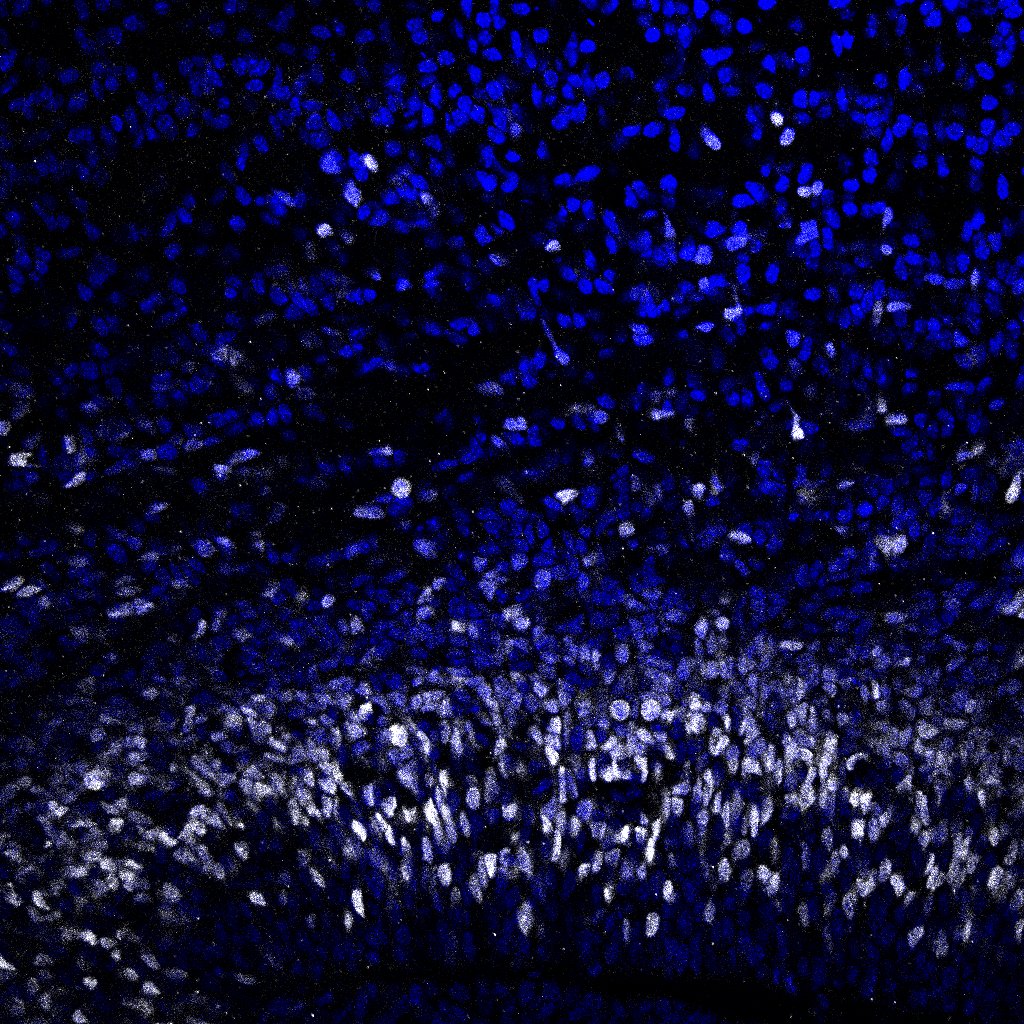

Supplement: Supplementary file 10 — Source data Fig. 5 [file 44318_2024_343_MOESM10_ESM.zip › Figure5/5B/Robo1OE_DAPI,TBR2.jpg]

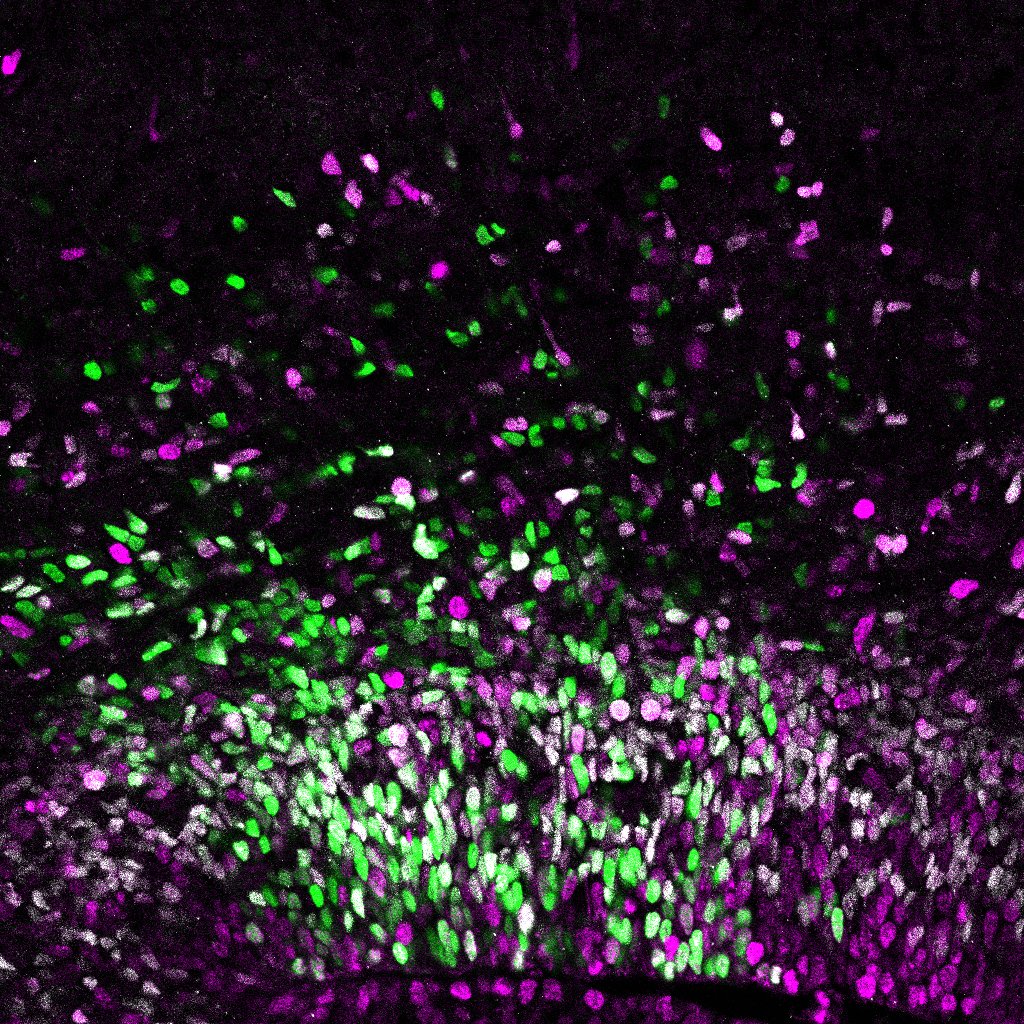

Supplement: Supplementary file 10 — Source data Fig. 5 [file 44318_2024_343_MOESM10_ESM.zip › Figure5/5B/Robo1OE_merge.jpg]

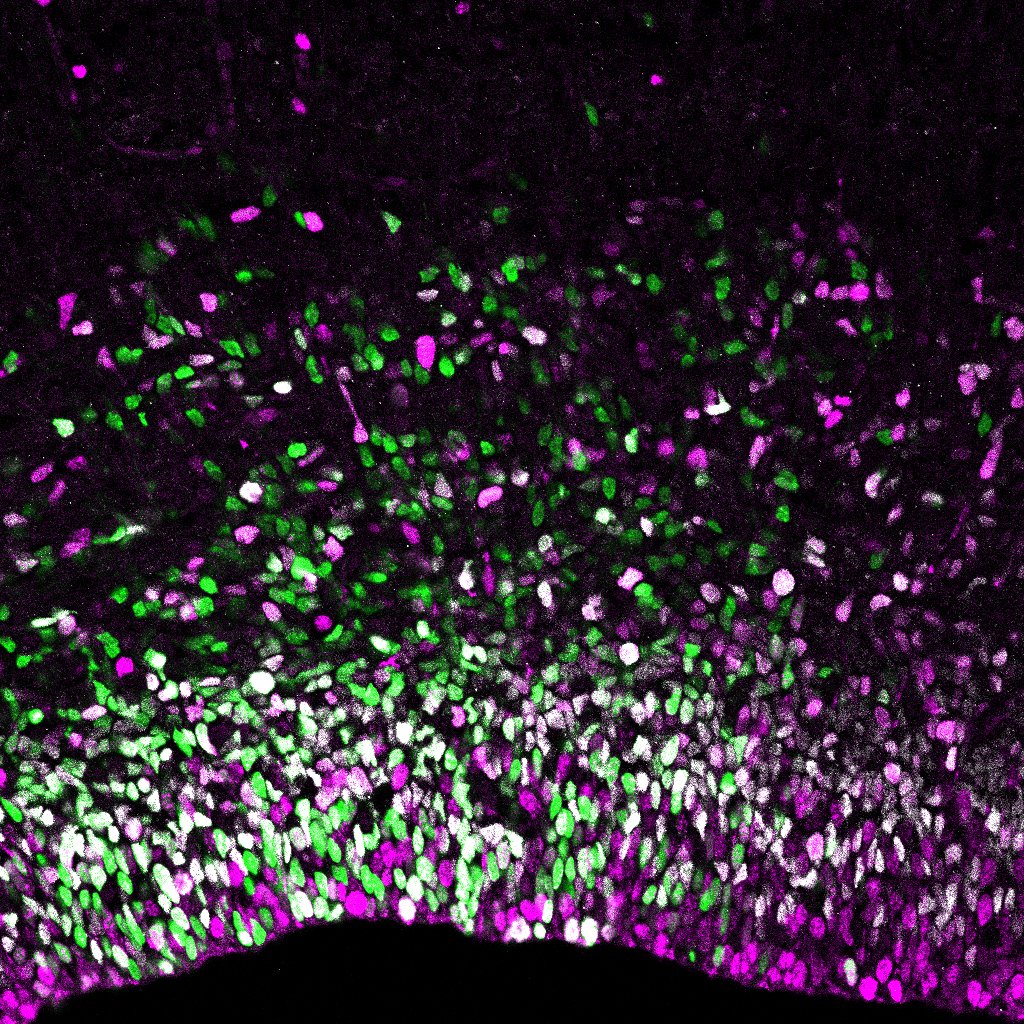

Supplement: Supplementary file 10 — Source data Fig. 5 [file 44318_2024_343_MOESM10_ESM.zip › Figure5/5B/Control_merge.jpg]

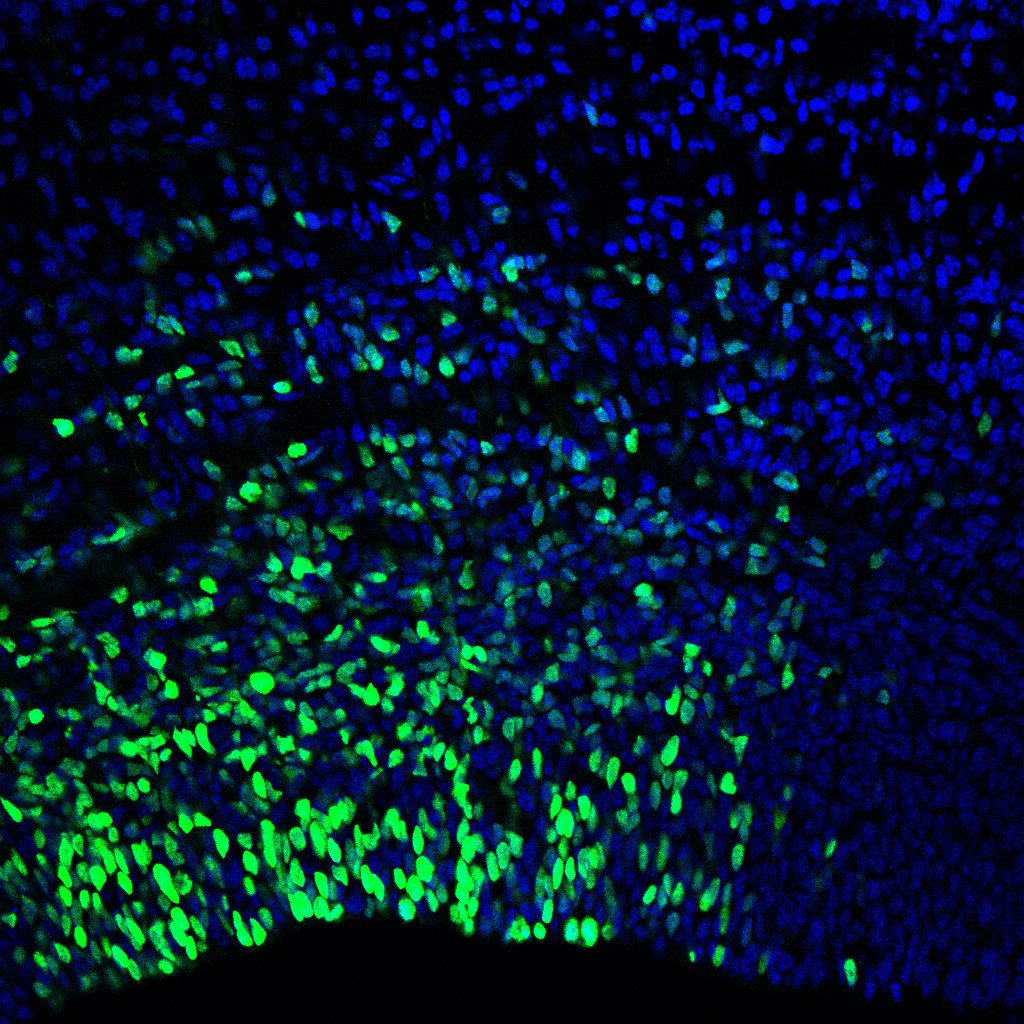

Supplement: Supplementary file 10 — Source data Fig. 5 [file 44318_2024_343_MOESM10_ESM.zip › Figure5/5B/Control_DAPI,GFP.jpg]

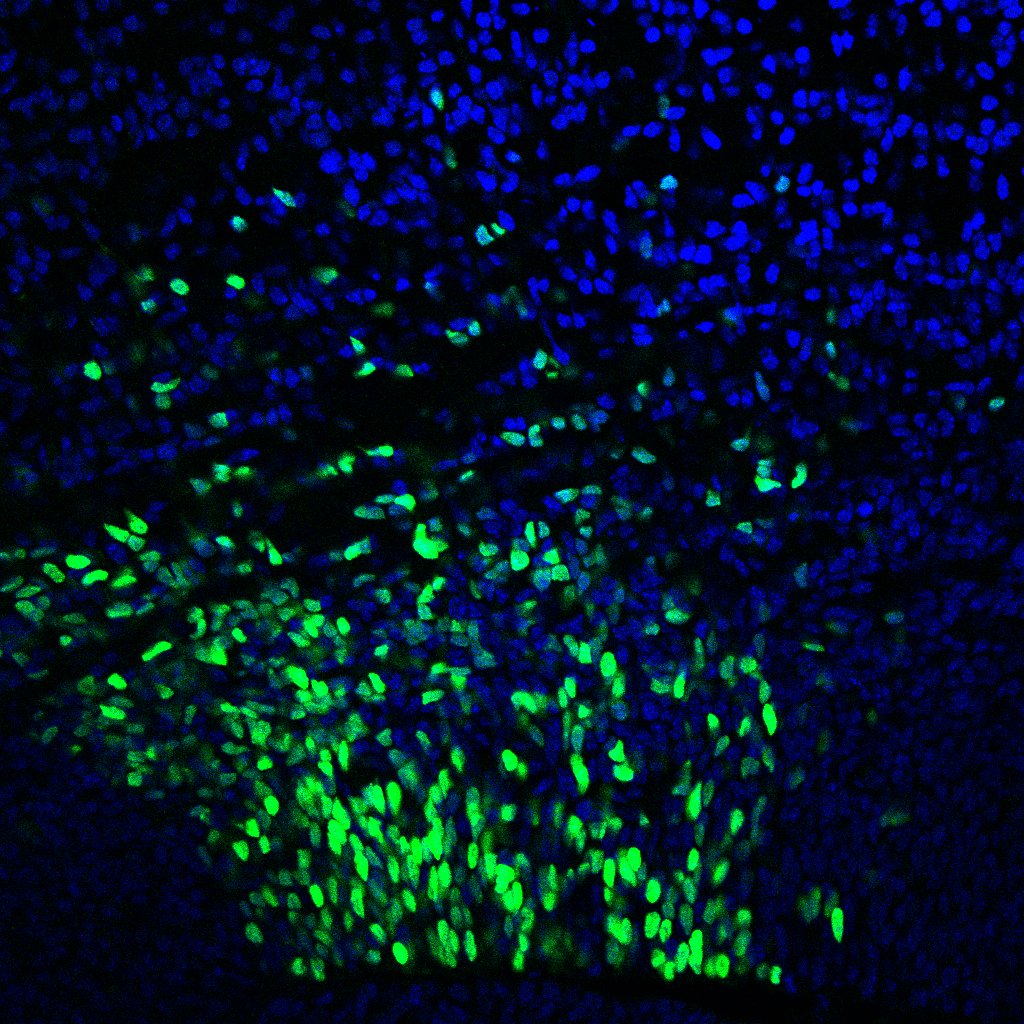

Supplement: Supplementary file 10 — Source data Fig. 5 [file 44318_2024_343_MOESM10_ESM.zip › Figure5/5B/Robo1OE_DAPI,GFP.jpg]

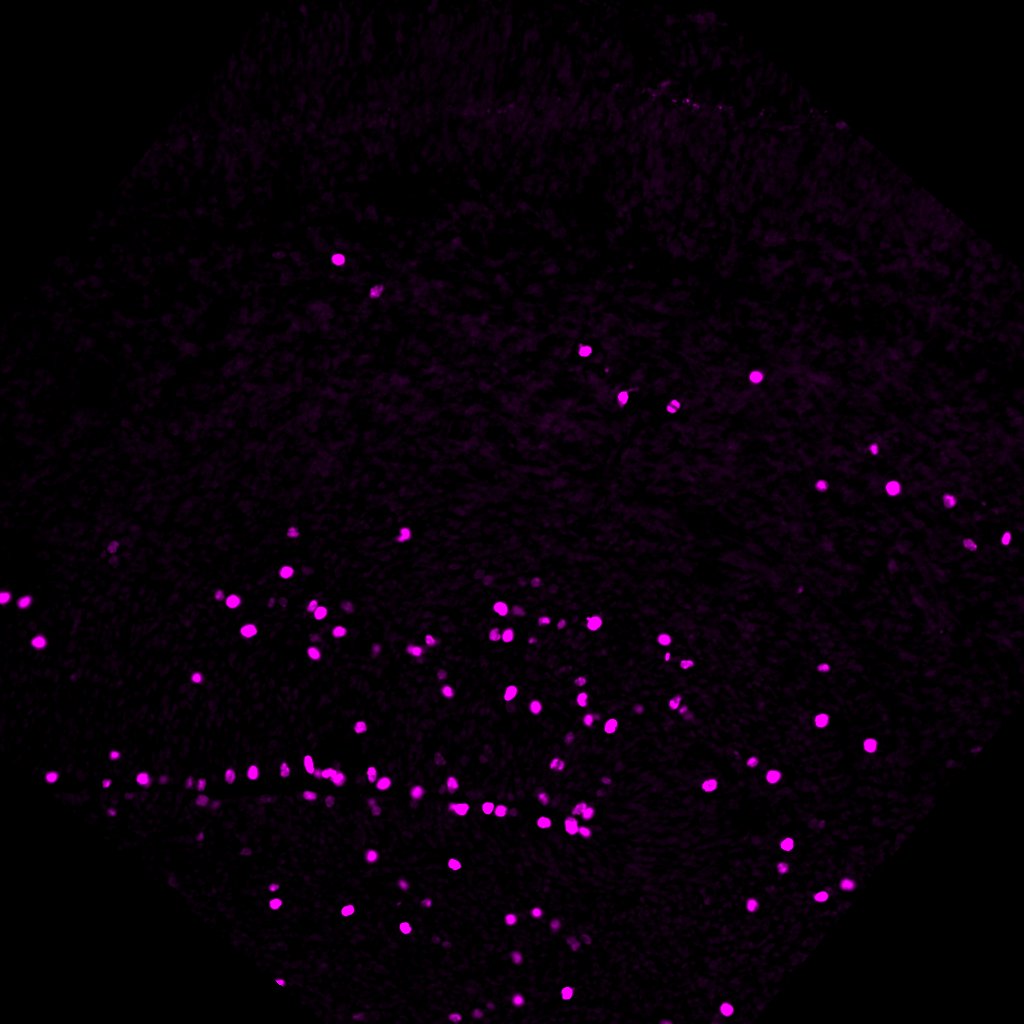

Supplement: Supplementary file 11 — Source data Fig. 8 [file 44318_2024_343_MOESM11_ESM.zip › Figure8/8F/shEzh2+FL-Auts2_PH3.jpg]

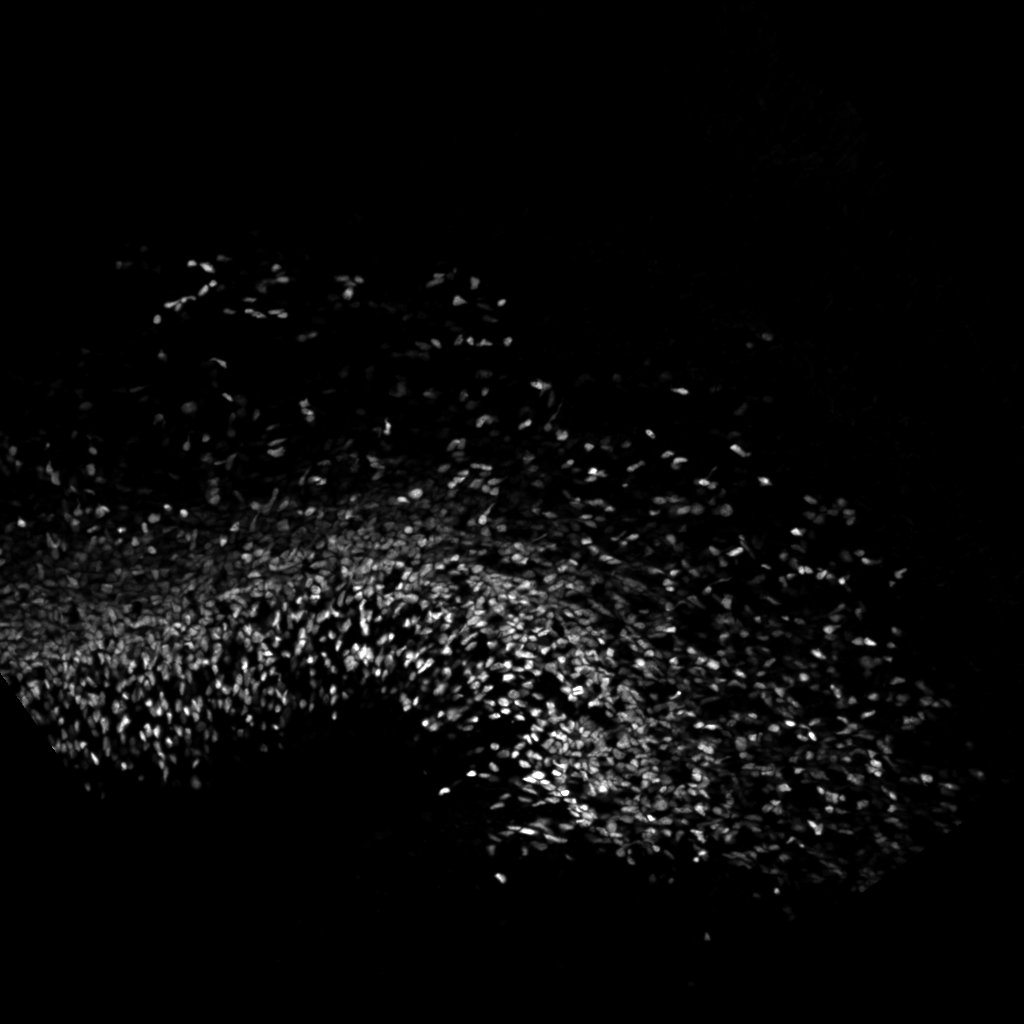

Supplement: Supplementary file 11 — Source data Fig. 8 [file 44318_2024_343_MOESM11_ESM.zip › Figure8/8F/shSuz12_TBR2.jpg]

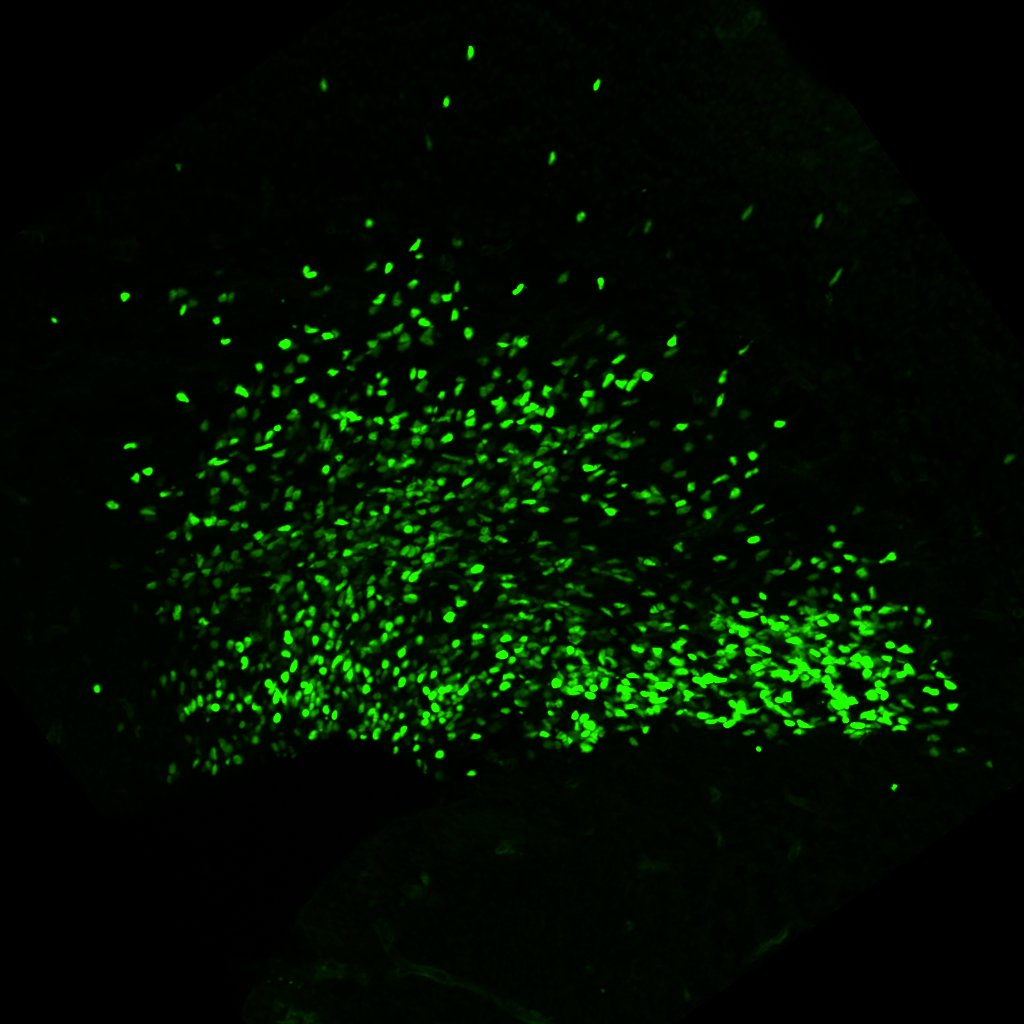

Supplement: Supplementary file 11 — Source data Fig. 8 [file 44318_2024_343_MOESM11_ESM.zip › Figure8/8F/shSuz12_GFP.jpg]

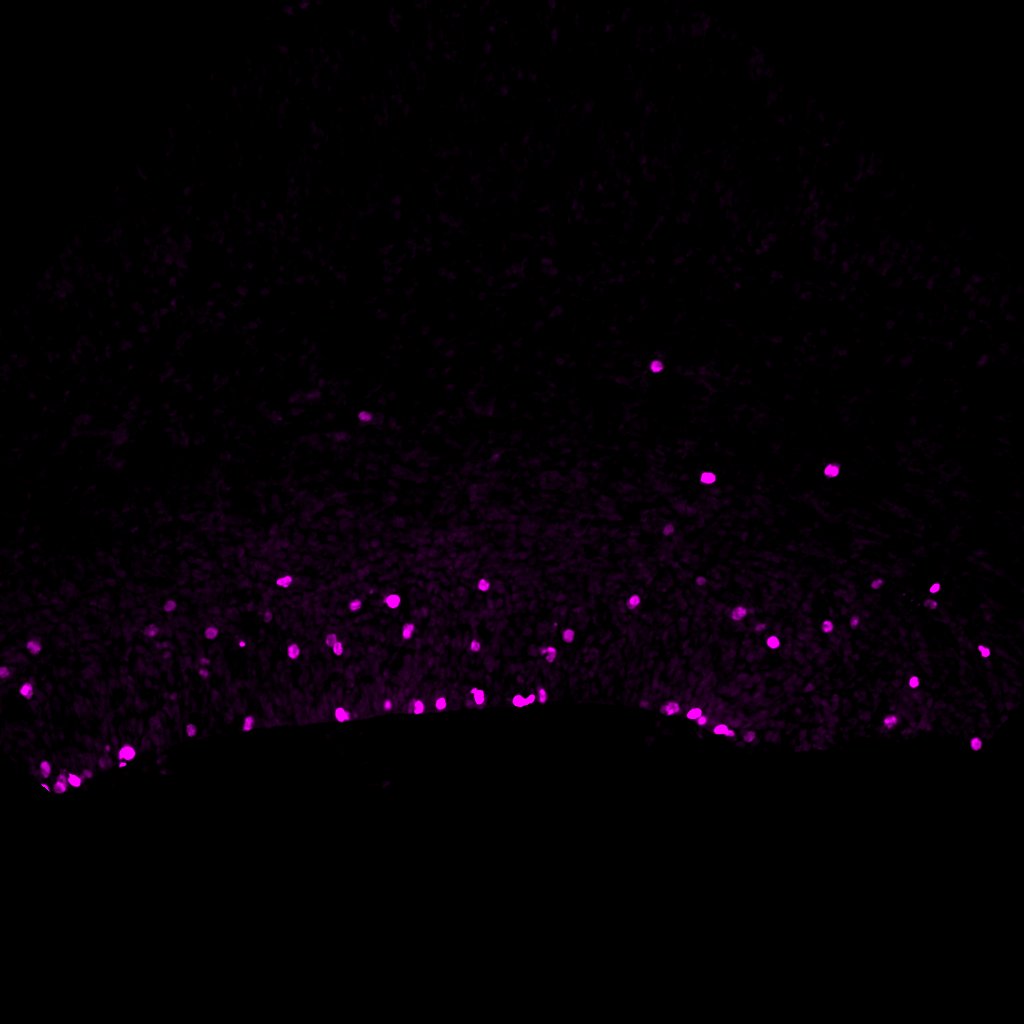

Supplement: Supplementary file 11 — Source data Fig. 8 [file 44318_2024_343_MOESM11_ESM.zip › Figure8/8F/shSuz12+FL-Auts2_PH3.jpg]

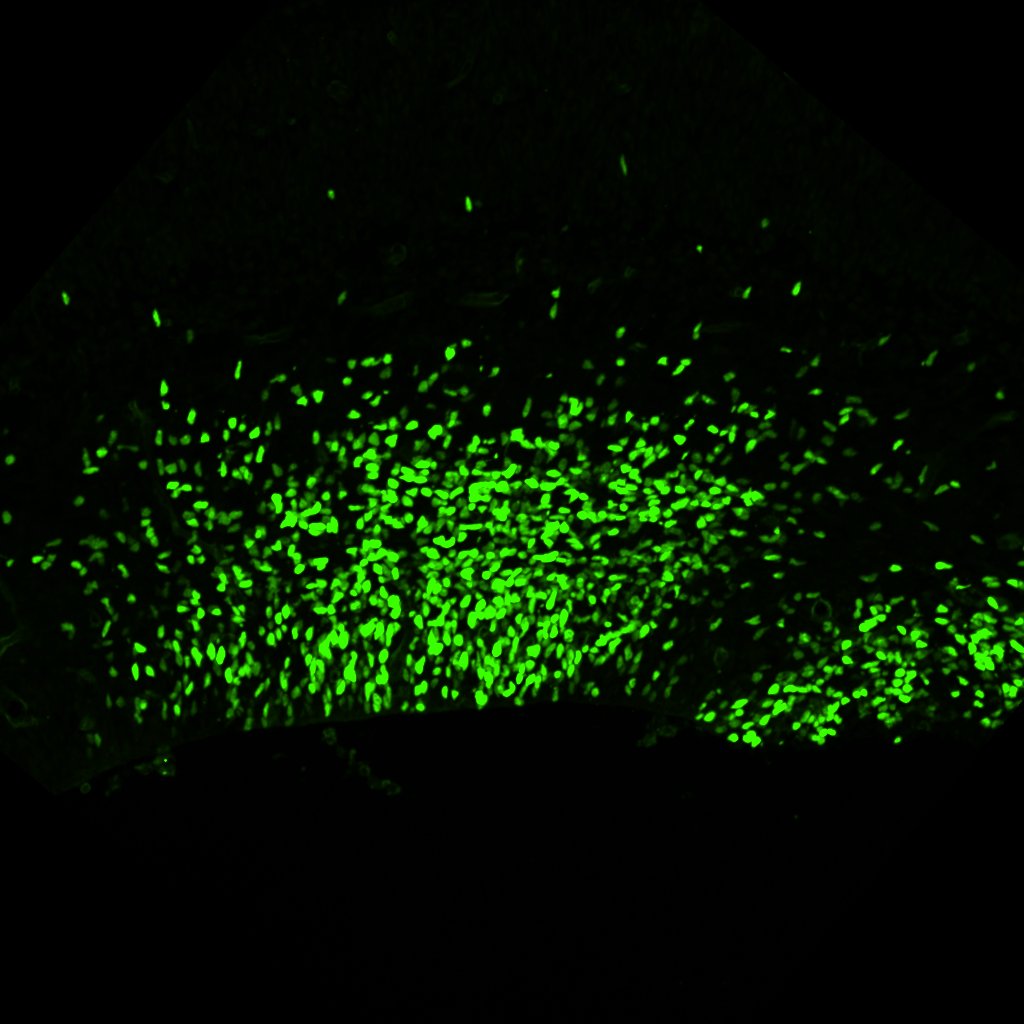

Supplement: Supplementary file 11 — Source data Fig. 8 [file 44318_2024_343_MOESM11_ESM.zip › Figure8/8F/shSuz12+FL-Auts2_GFP.jpg]

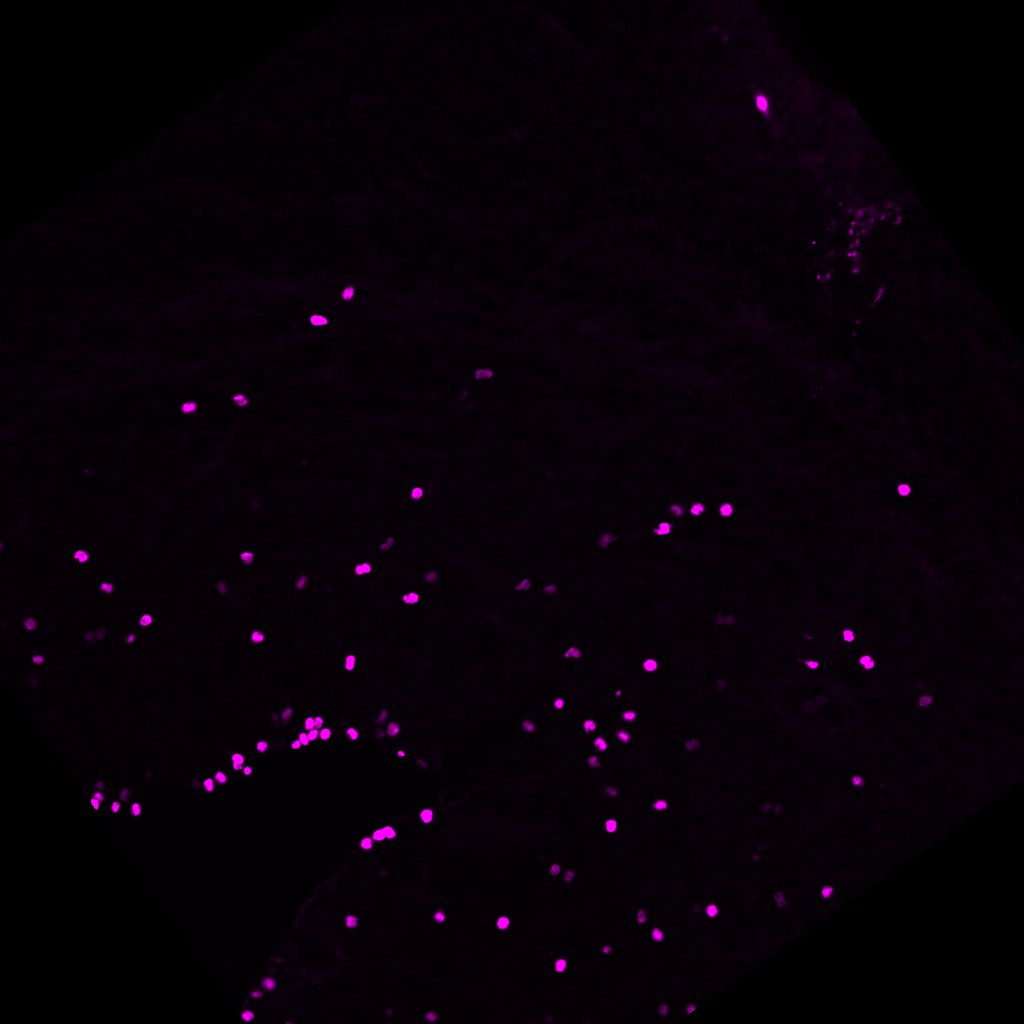

Supplement: Supplementary file 11 — Source data Fig. 8 [file 44318_2024_343_MOESM11_ESM.zip › Figure8/8F/shSuz12_PH3.jpg]

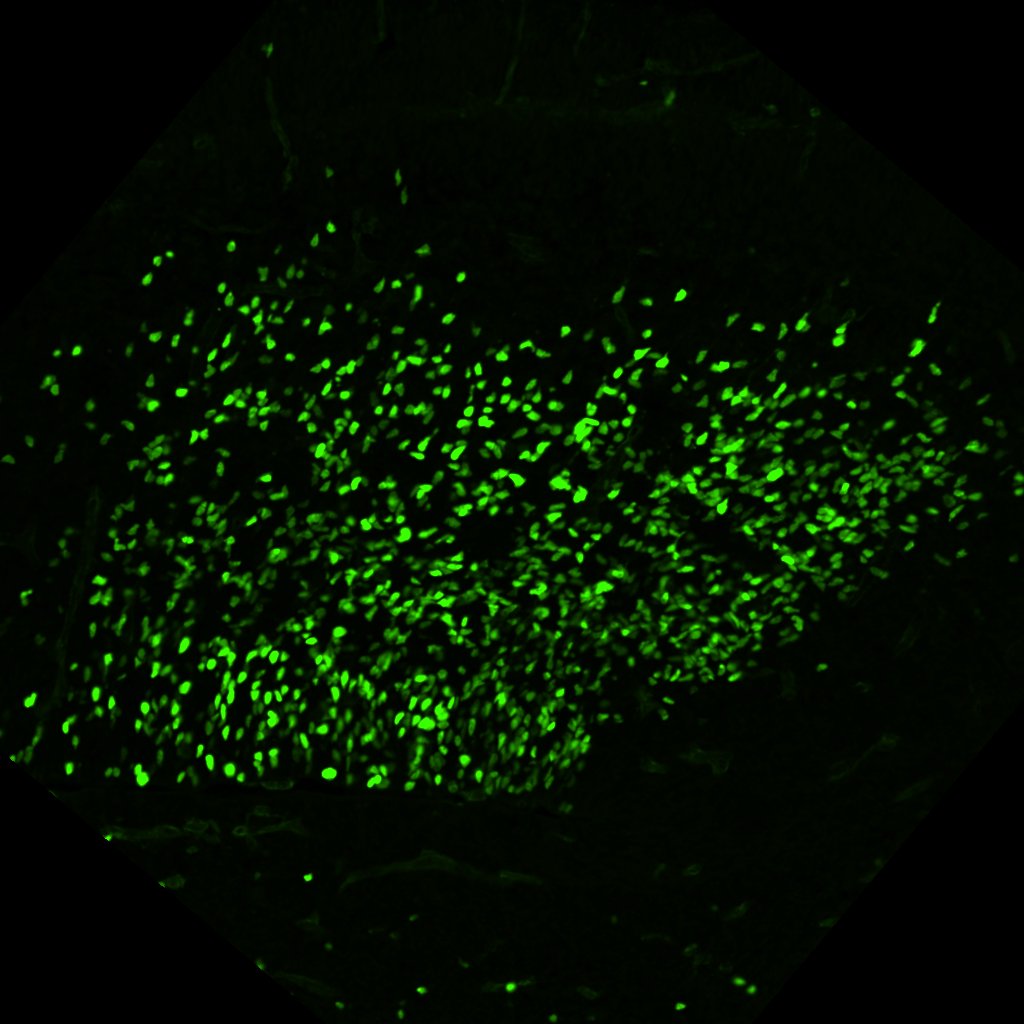

Supplement: Supplementary file 11 — Source data Fig. 8 [file 44318_2024_343_MOESM11_ESM.zip › Figure8/8F/shEzh2+FL-Auts2_GFP.jpg]

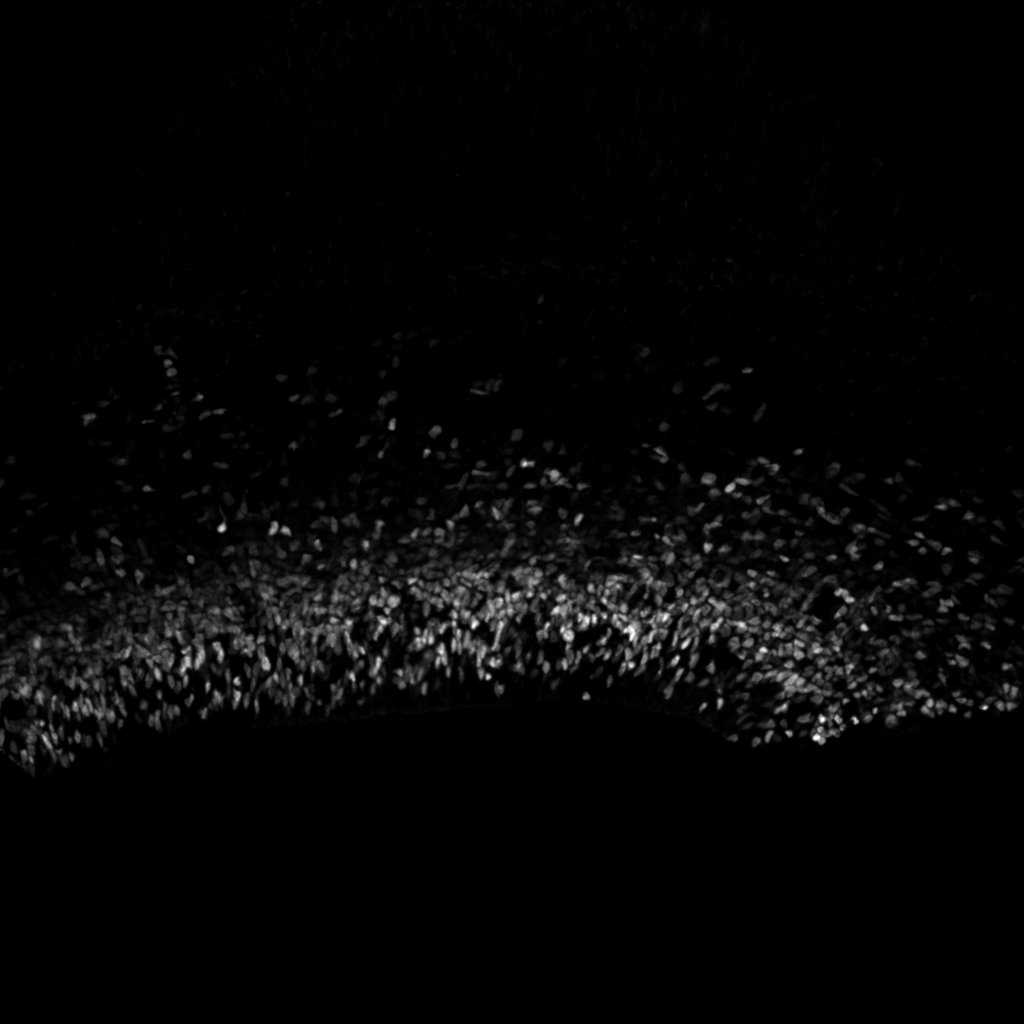

Supplement: Supplementary file 11 — Source data Fig. 8 [file 44318_2024_343_MOESM11_ESM.zip › Figure8/8F/shSuz12+FL-Auts2_TBR2.jpg]

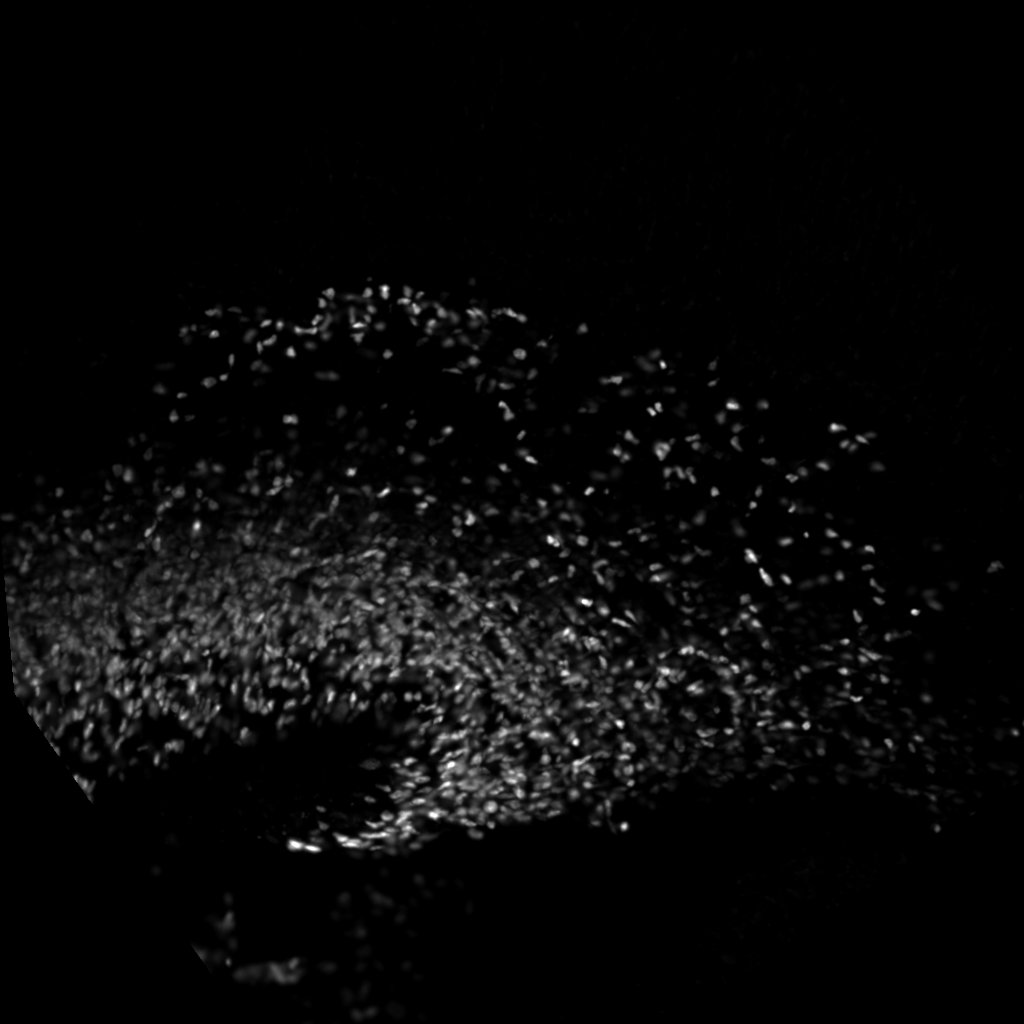

Supplement: Supplementary file 11 — Source data Fig. 8 [file 44318_2024_343_MOESM11_ESM.zip › Figure8/8F/shScramble_TBR2.jpg]

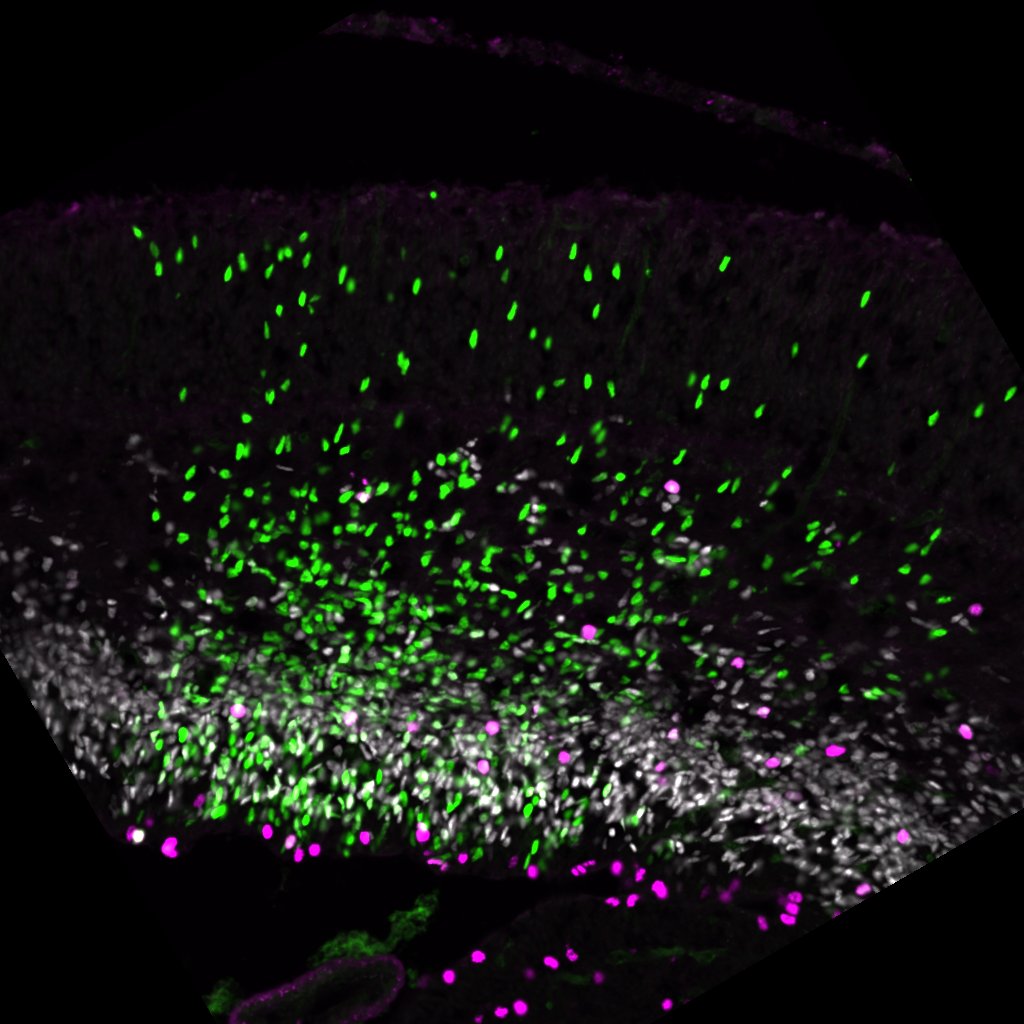

Supplement: Supplementary file 11 — Source data Fig. 8 [file 44318_2024_343_MOESM11_ESM.zip › Figure8/8F/shEzh2_merge.jpg]

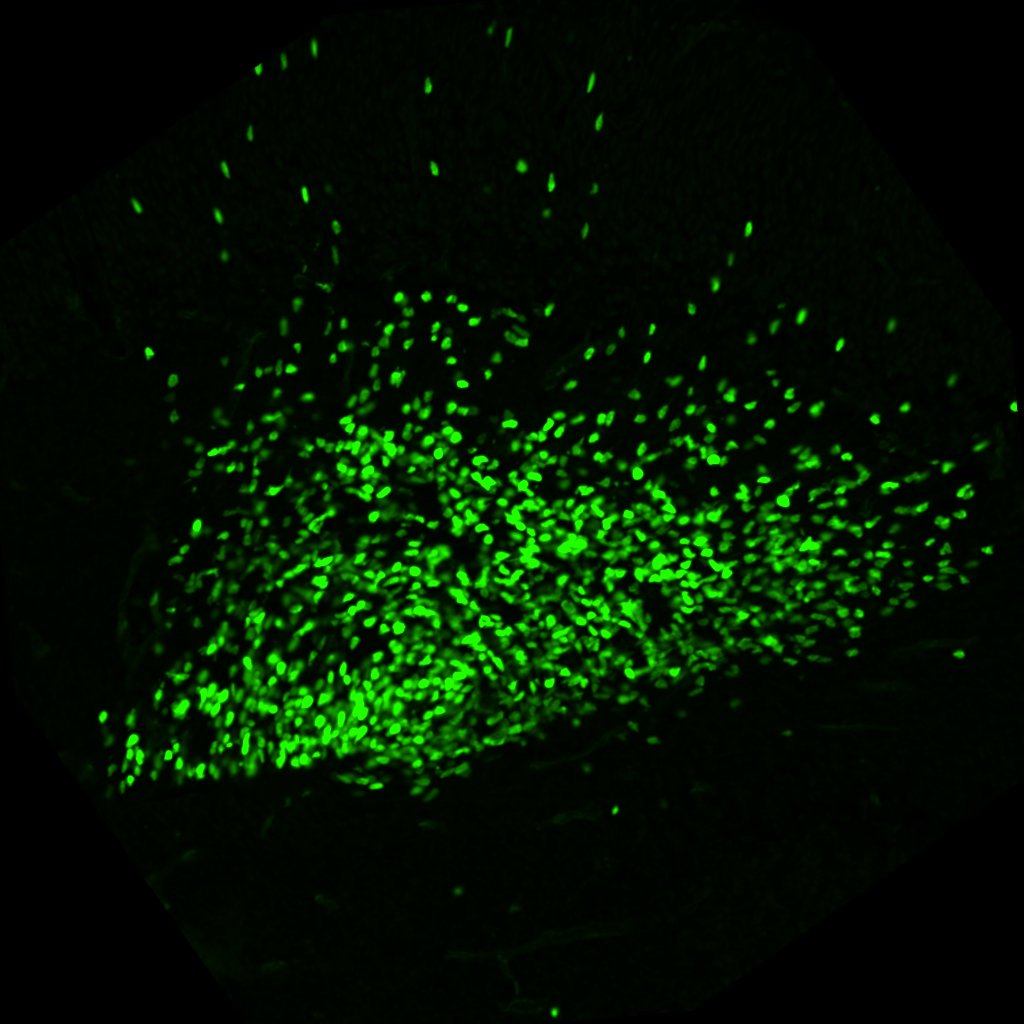

Supplement: Supplementary file 11 — Source data Fig. 8 [file 44318_2024_343_MOESM11_ESM.zip › Figure8/8F/shScramble_GFP.jpg]

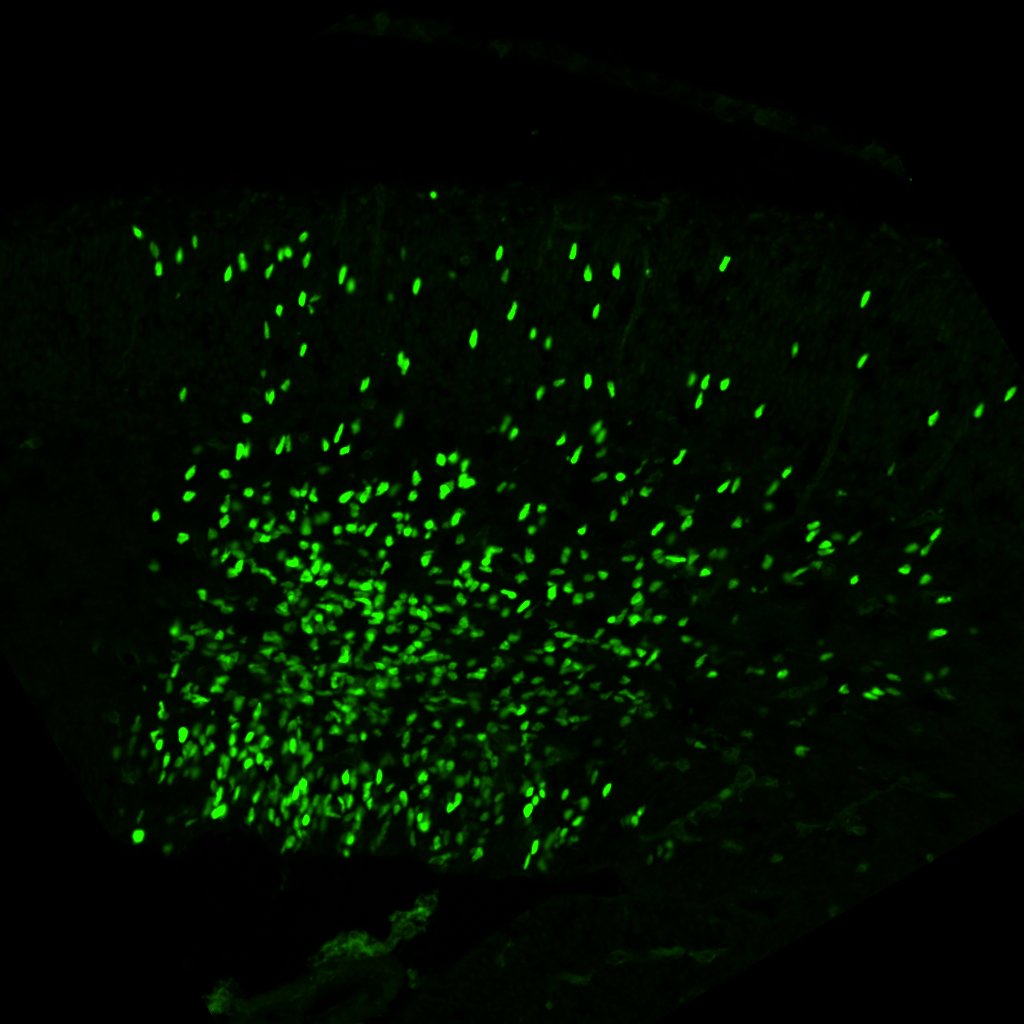

Supplement: Supplementary file 11 — Source data Fig. 8 [file 44318_2024_343_MOESM11_ESM.zip › Figure8/8F/shEzh2_GFP.jpg]

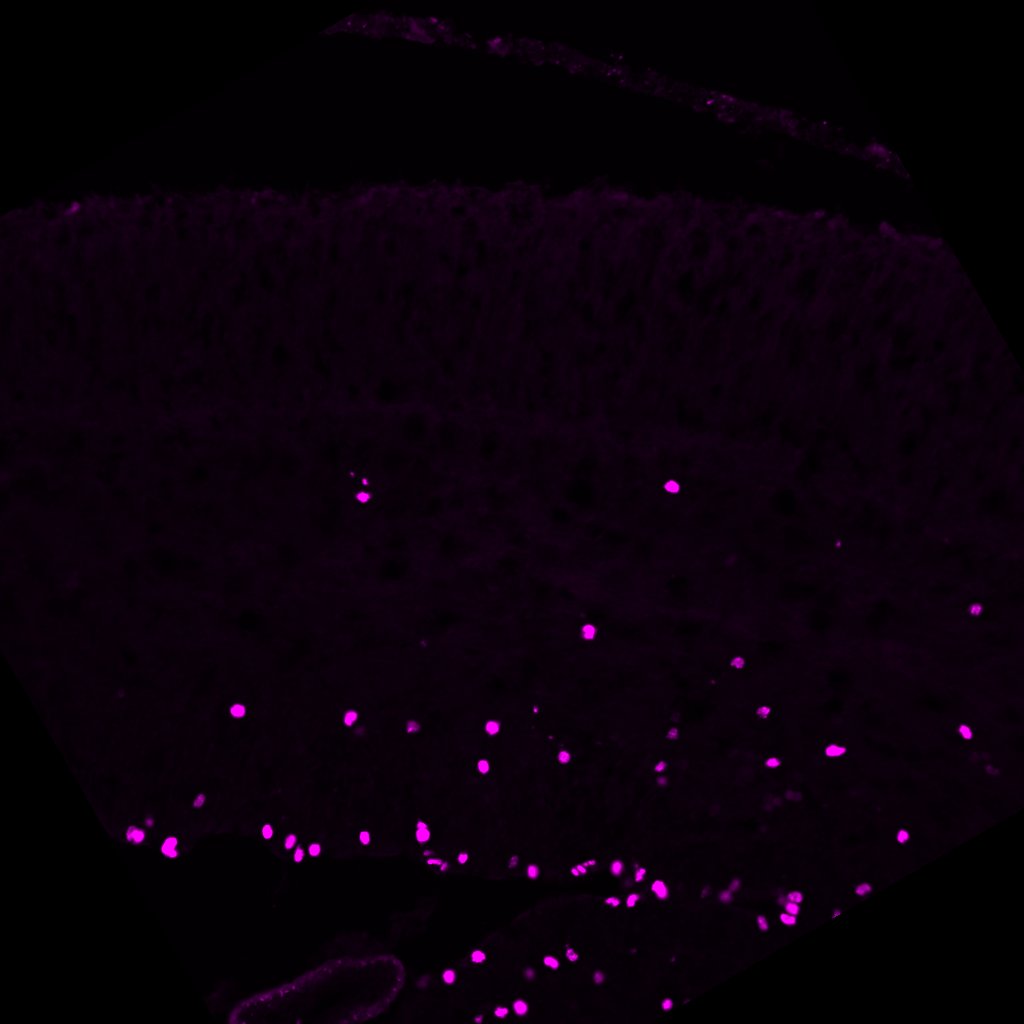

Supplement: Supplementary file 11 — Source data Fig. 8 [file 44318_2024_343_MOESM11_ESM.zip › Figure8/8F/shEzh2_PH3.jpg]

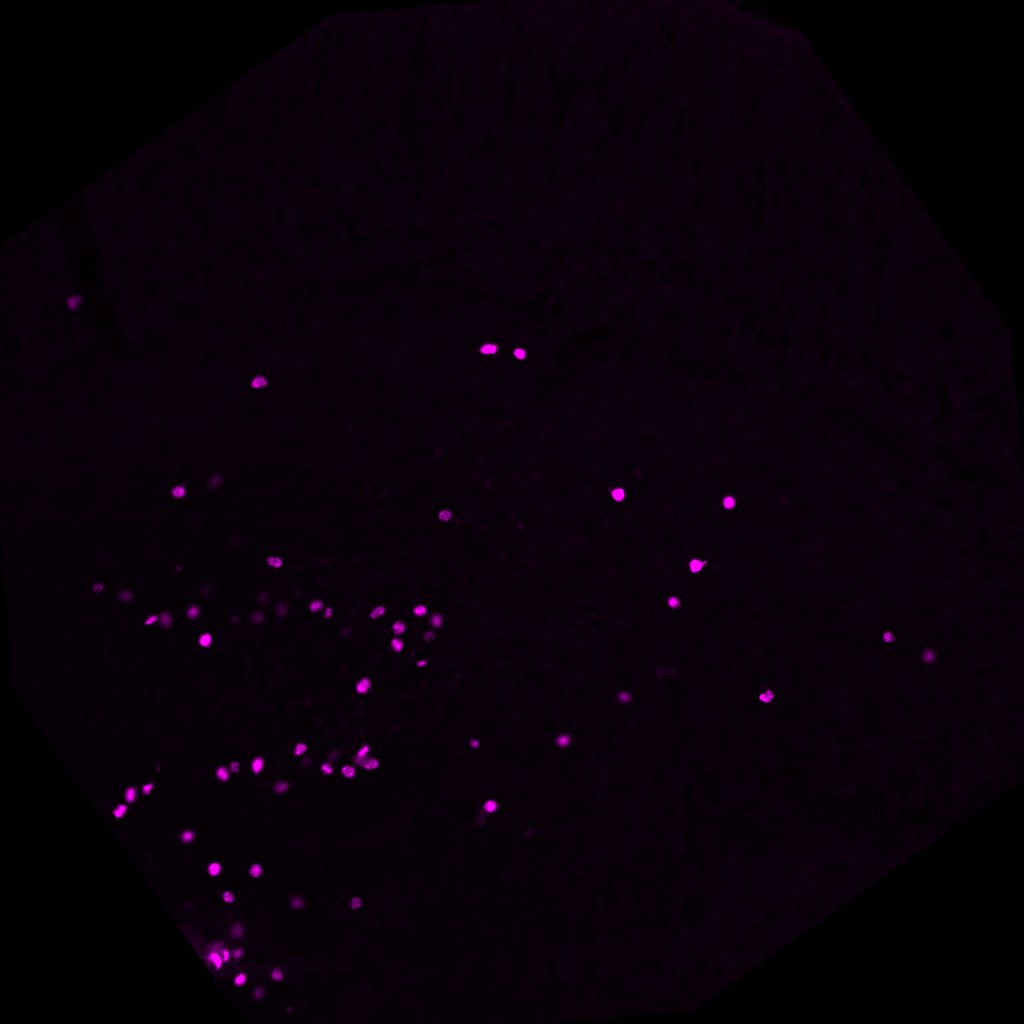

Supplement: Supplementary file 11 — Source data Fig. 8 [file 44318_2024_343_MOESM11_ESM.zip › Figure8/8F/shScramble_PH3.jpg]

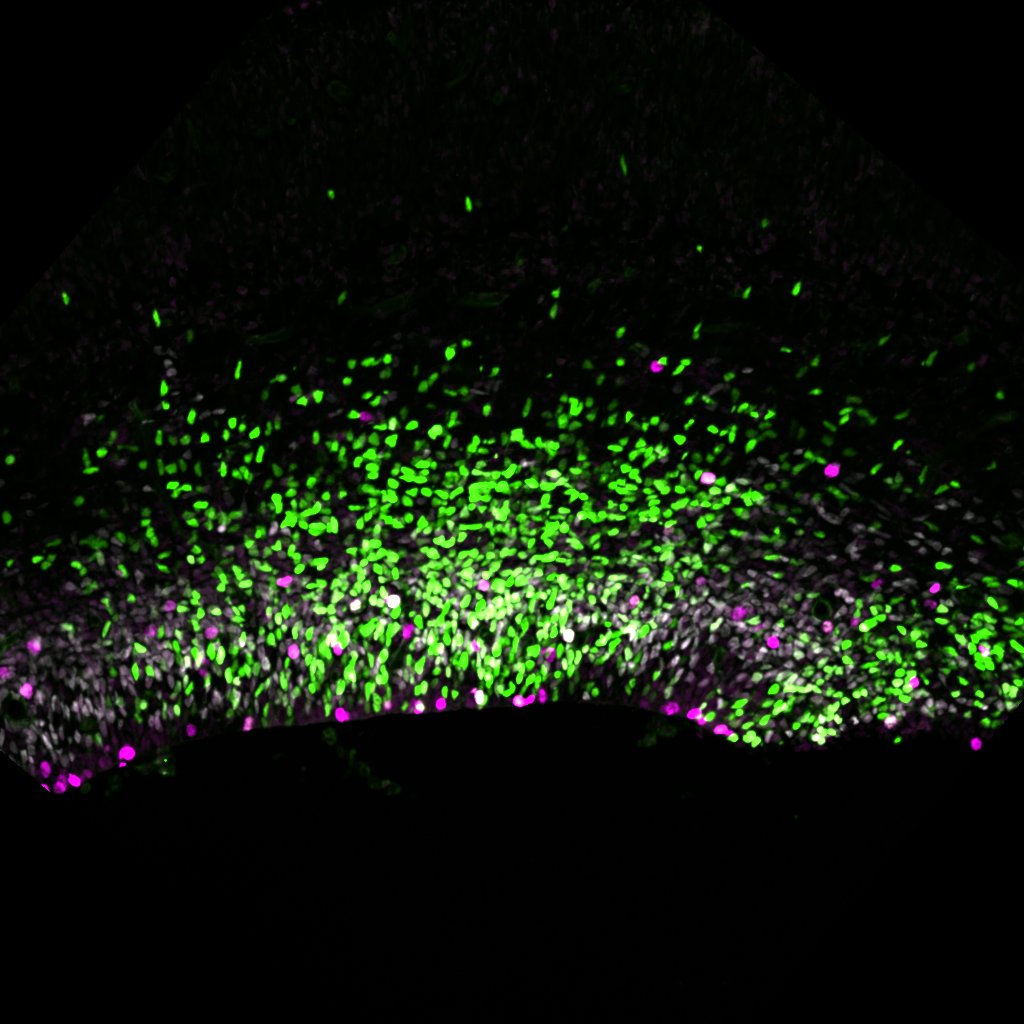

Supplement: Supplementary file 11 — Source data Fig. 8 [file 44318_2024_343_MOESM11_ESM.zip › Figure8/8F/shSuz12+FL-Auts2_merge.jpg]

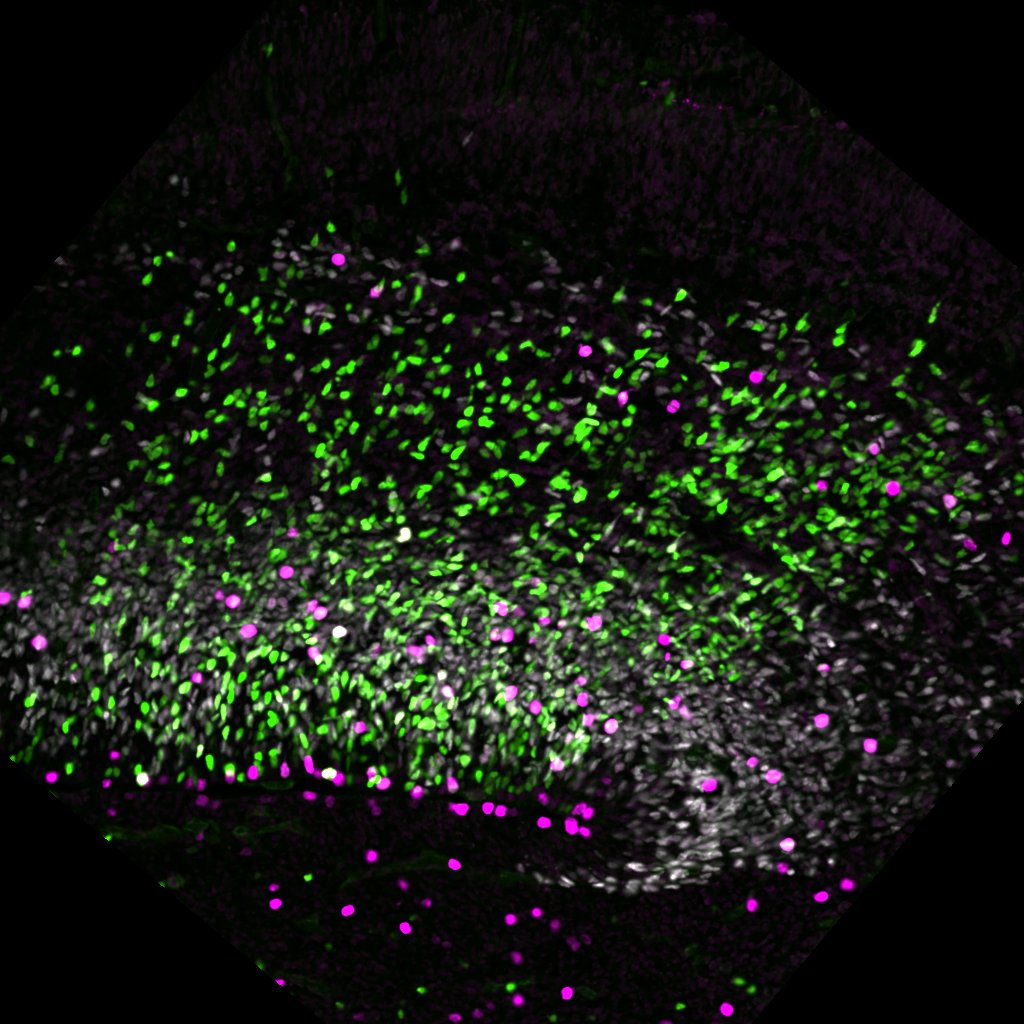

Supplement: Supplementary file 11 — Source data Fig. 8 [file 44318_2024_343_MOESM11_ESM.zip › Figure8/8F/shEzh2+FL-Auts2_merge.jpg]

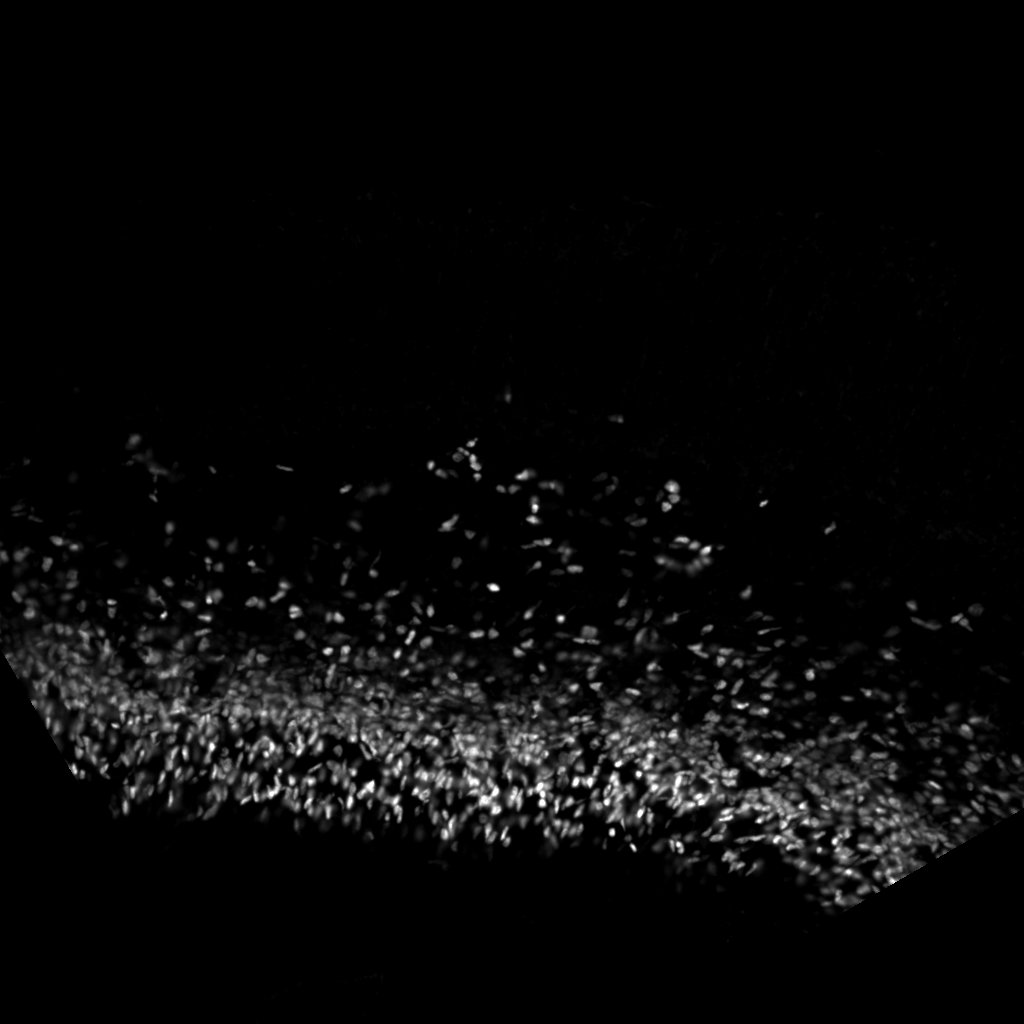

Supplement: Supplementary file 11 — Source data Fig. 8 [file 44318_2024_343_MOESM11_ESM.zip › Figure8/8F/shEzh2_TBR2.jpg]

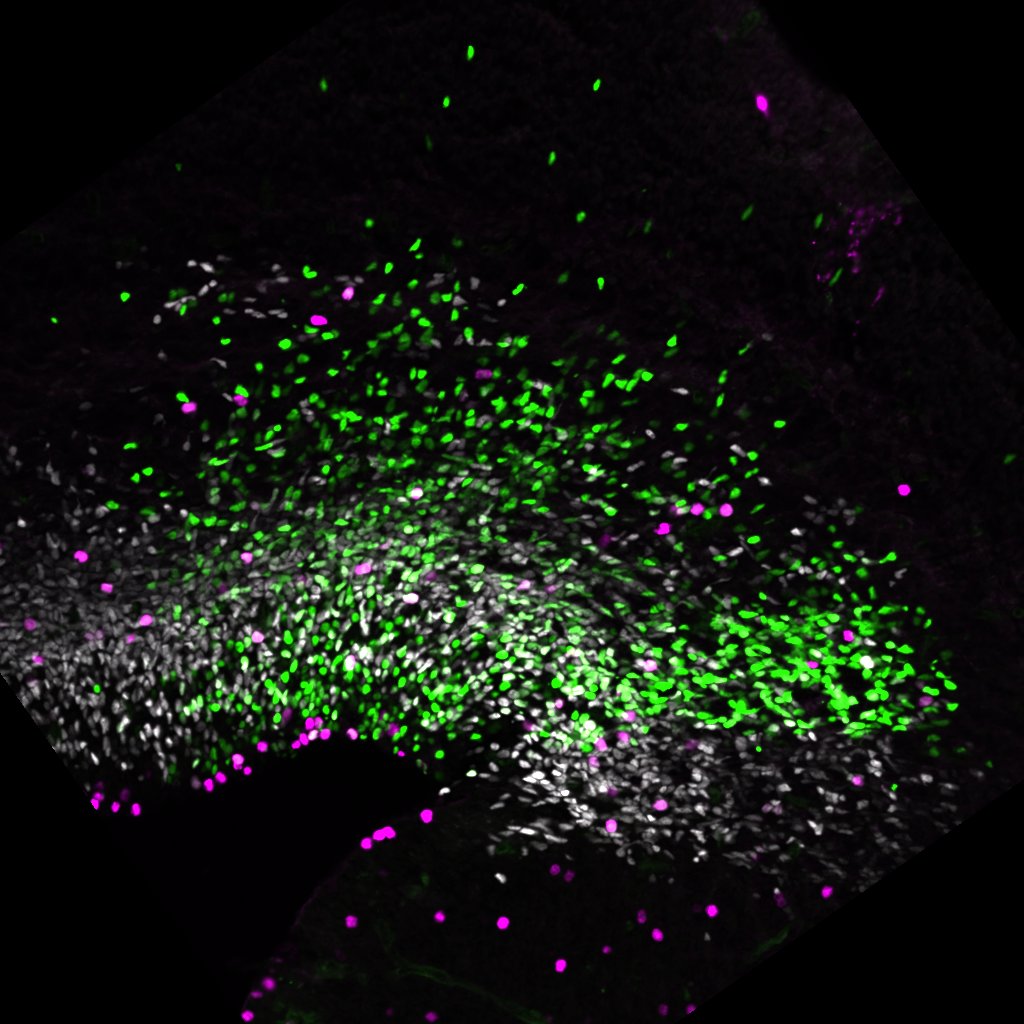

Supplement: Supplementary file 11 — Source data Fig. 8 [file 44318_2024_343_MOESM11_ESM.zip › Figure8/8F/shSuz12_merge.jpg]

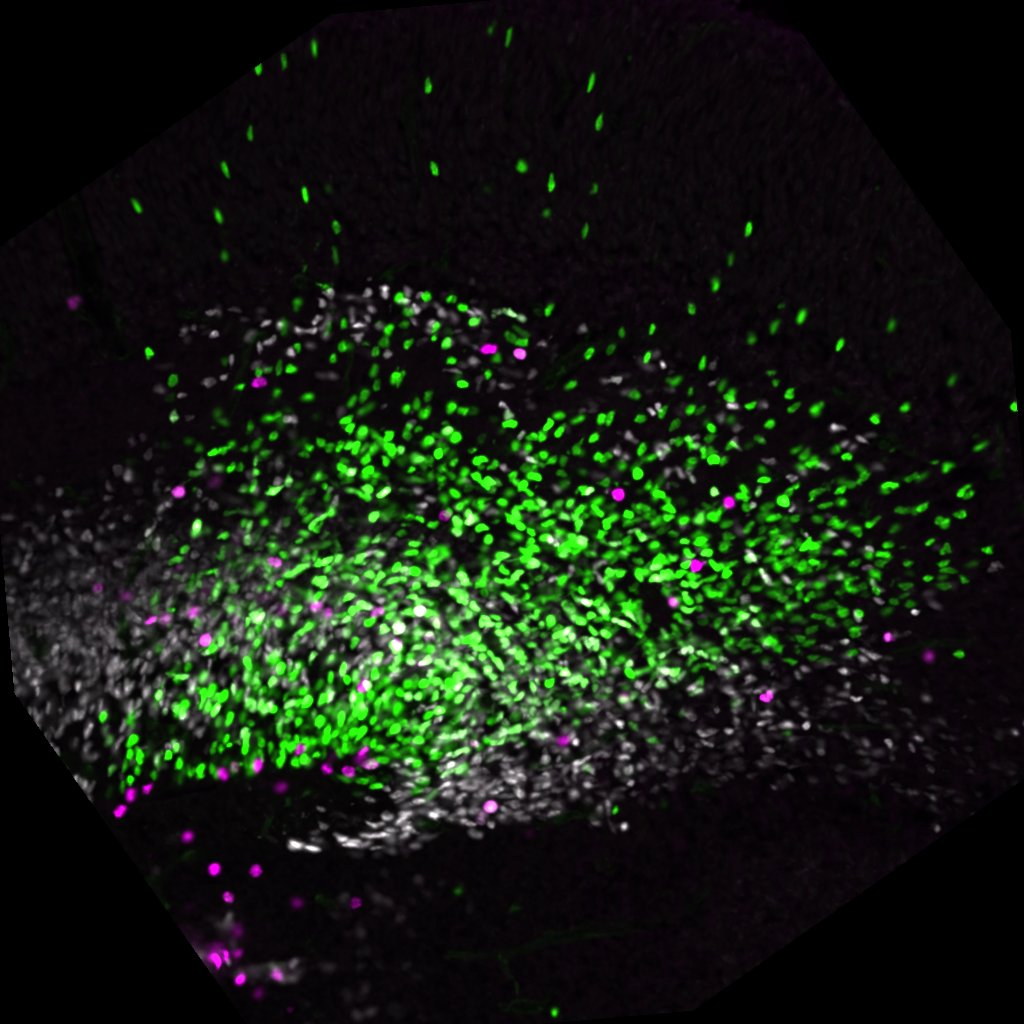

Supplement: Supplementary file 11 — Source data Fig. 8 [file 44318_2024_343_MOESM11_ESM.zip › Figure8/8F/shScramble_merge.jpg]

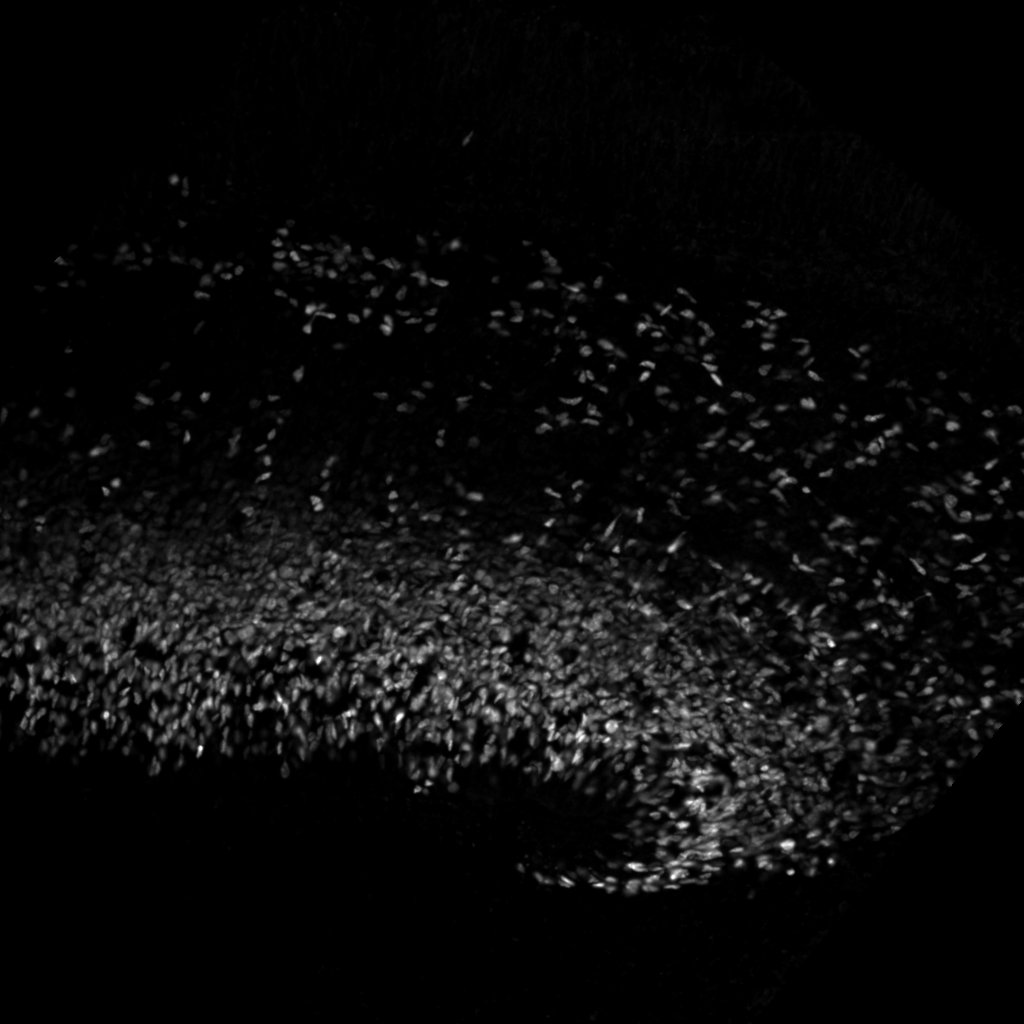

Supplement: Supplementary file 11 — Source data Fig. 8 [file 44318_2024_343_MOESM11_ESM.zip › Figure8/8F/shEzh2+FL-Auts2_TBR2.jpg]
