# Supplementary figures and images for: The microcephaly-associated transcriptional regulator AUTS2 cooperates with Polycomb complex PRC2 to produce upper-layer neurons in mice (part 2 of 2)
Source: EMBO J. 2025 Jan 15;44(5):1354–78. doi: 10.1038/s44318-024-00343-7 (PMC11876313; doi:10.1038/s44318-024-00343-7)

## Slide 1
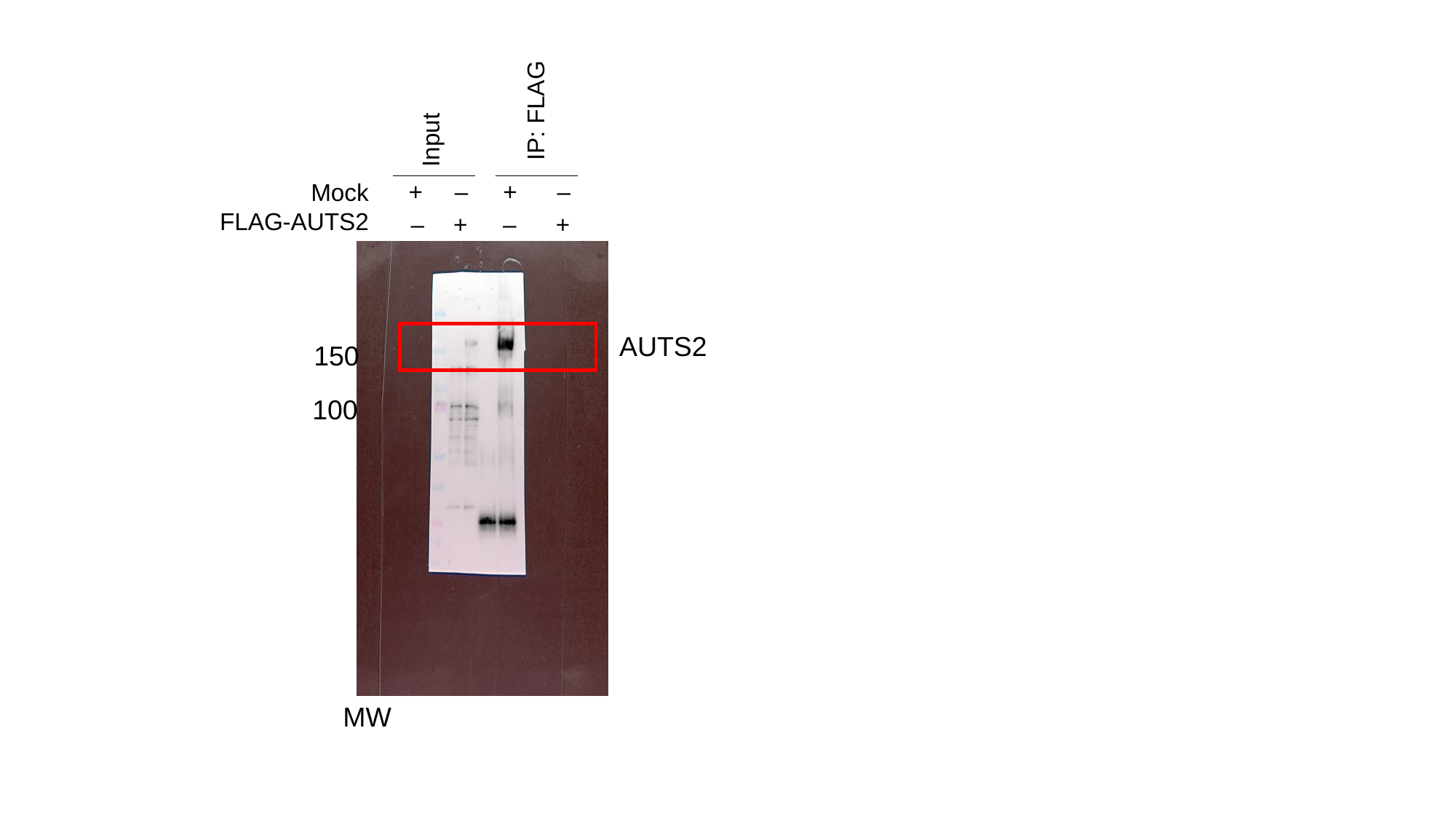

IP: FLAG
Input
+
–
+
–
Mock
FLAG-AUTS2
–
+
–
+
AUTS2
150
100
MW

Supplement: Supplementary file 11 — Source data Fig. 8 [file 44318_2024_343_MOESM11_ESM.zip › Figure8/8A/FLAG-AUTS2.pptx]

## Slide 1
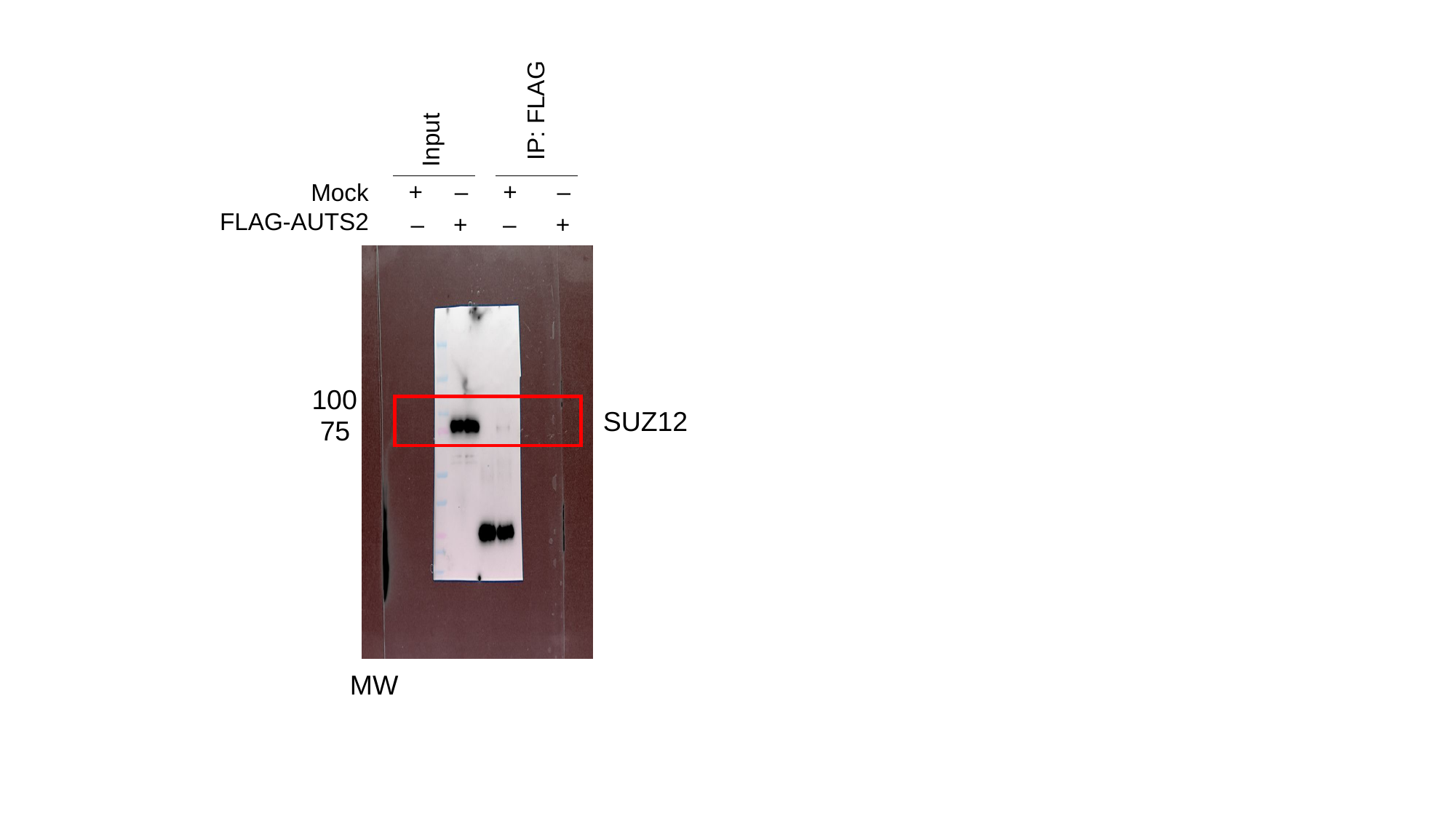

IP: FLAG
Input
+
–
+
–
Mock
FLAG-AUTS2
–
+
–
+
100
SUZ12
75
MW

Supplement: Supplementary file 11 — Source data Fig. 8 [file 44318_2024_343_MOESM11_ESM.zip › Figure8/8A/SUZ12.pptx]

## Slide 1
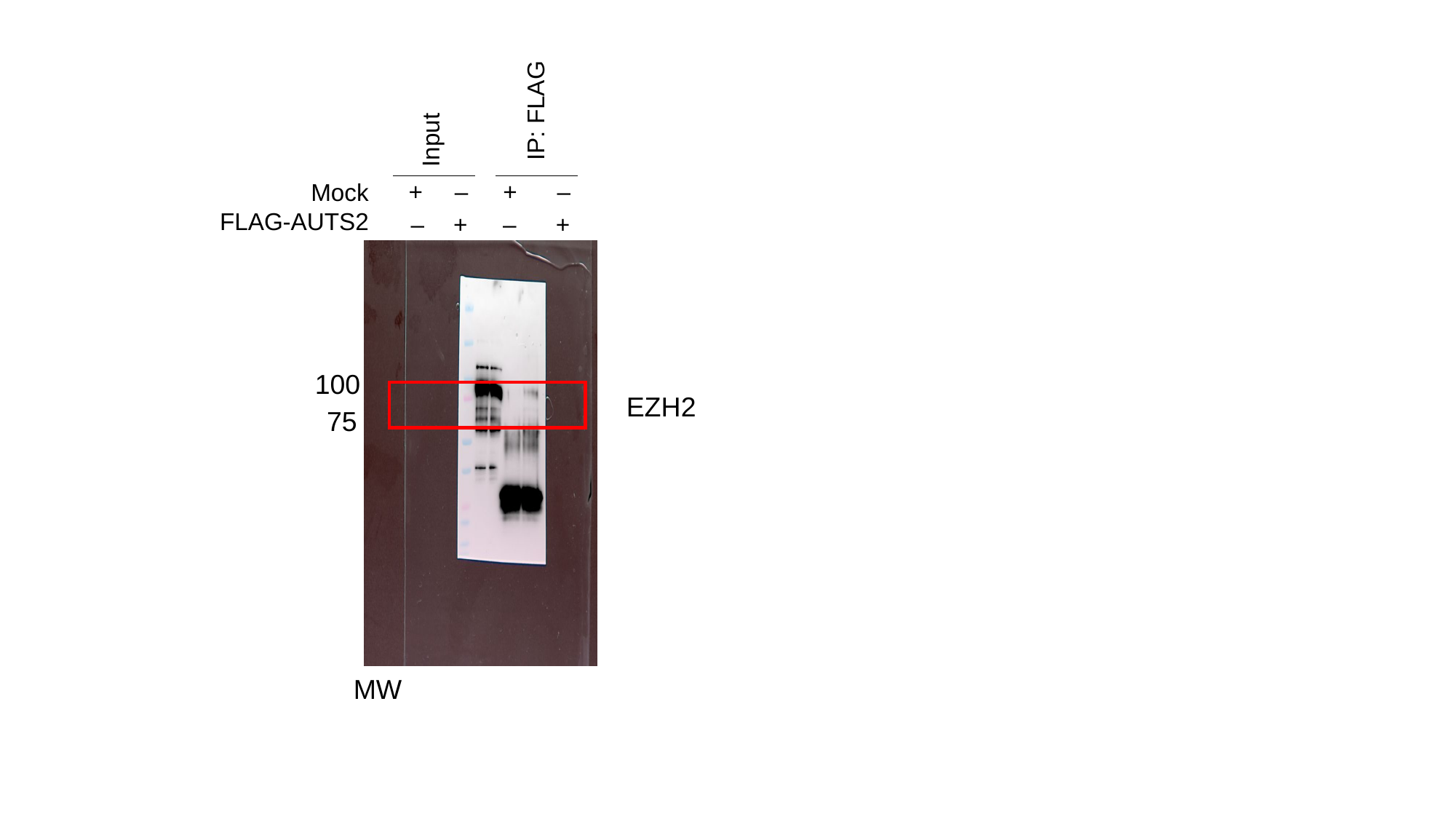

IP: FLAG
Input
+
–
+
–
Mock
FLAG-AUTS2
–
+
–
+
100
EZH2
75
MW

Supplement: Supplementary file 11 — Source data Fig. 8 [file 44318_2024_343_MOESM11_ESM.zip › Figure8/8A/EZH2.pptx]

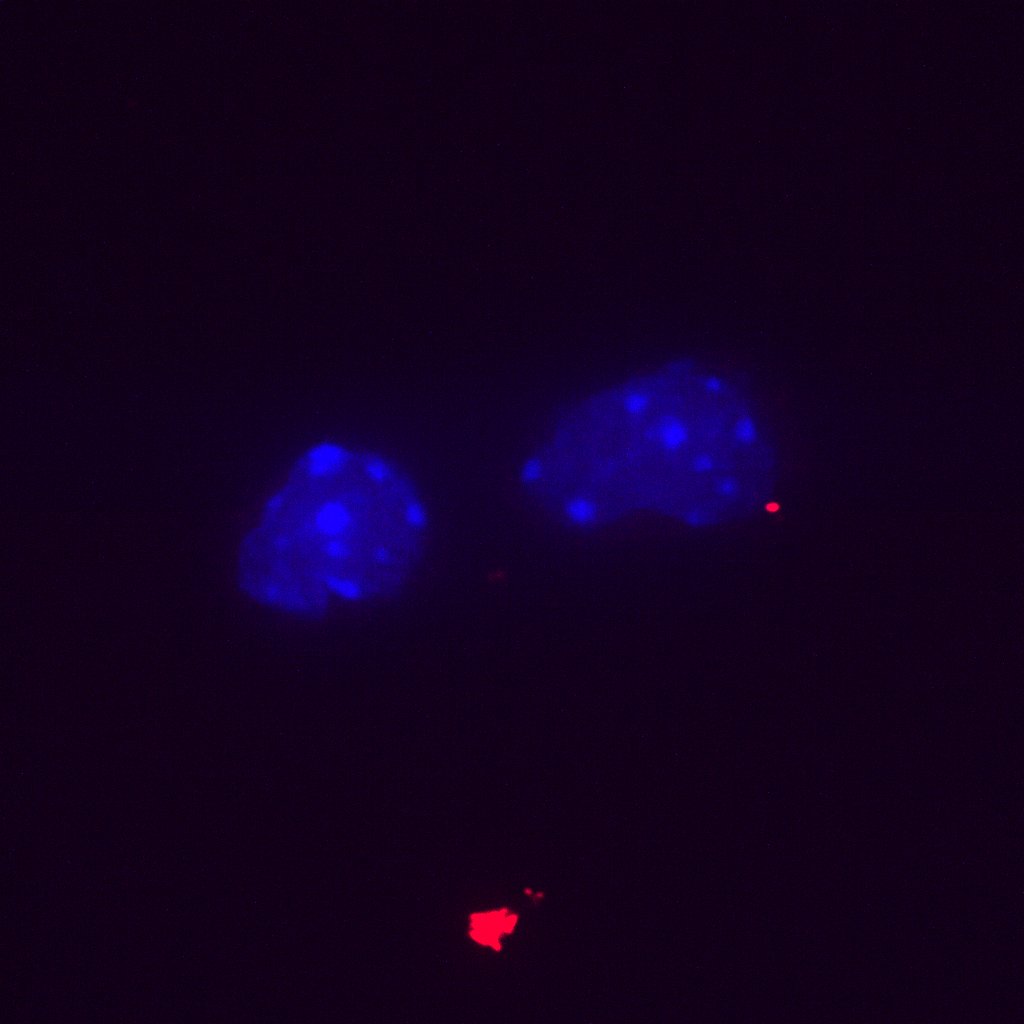

Supplement: Supplementary file 11 — Source data Fig. 8 [file 44318_2024_343_MOESM11_ESM.zip › Figure8/8C/EZH2_only.jpg]

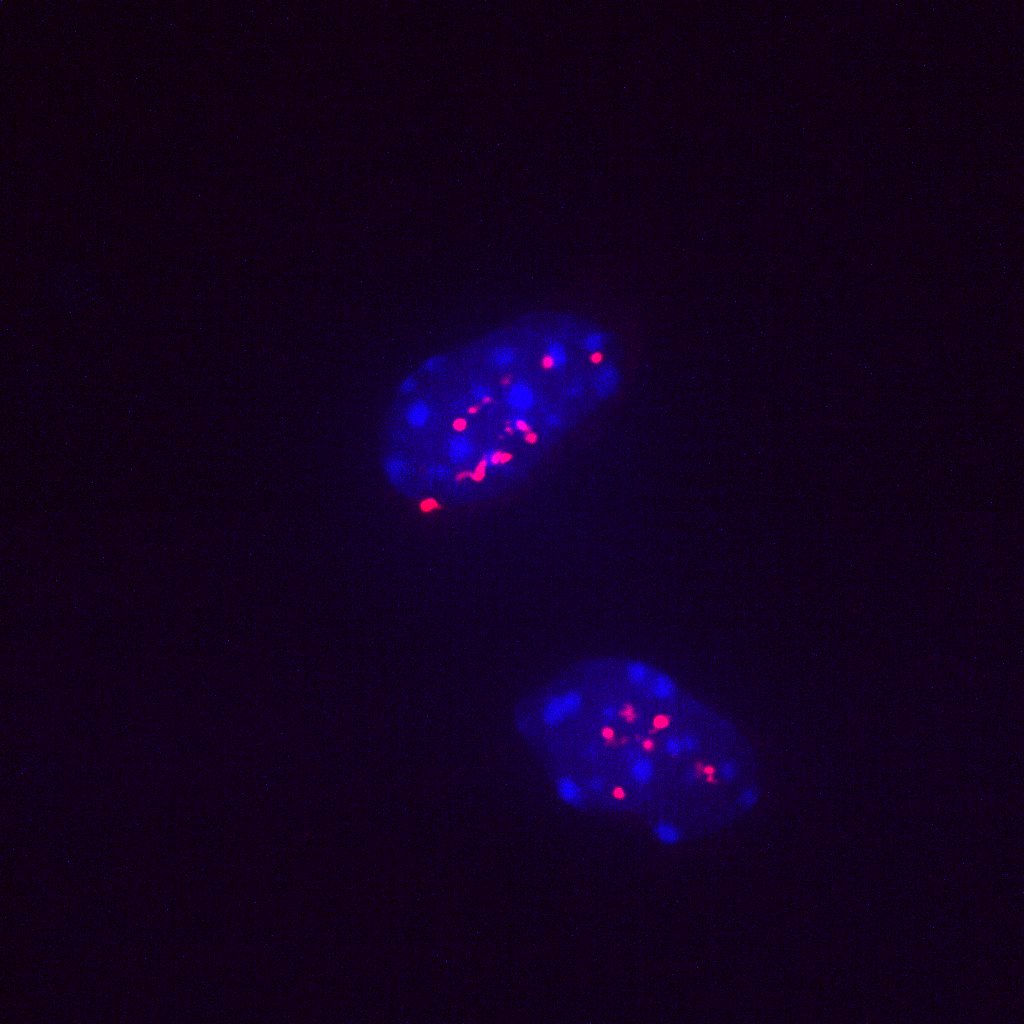

Supplement: Supplementary file 11 — Source data Fig. 8 [file 44318_2024_343_MOESM11_ESM.zip › Figure8/8C/AUTS2,EZH2.jpg]

## Slide 1
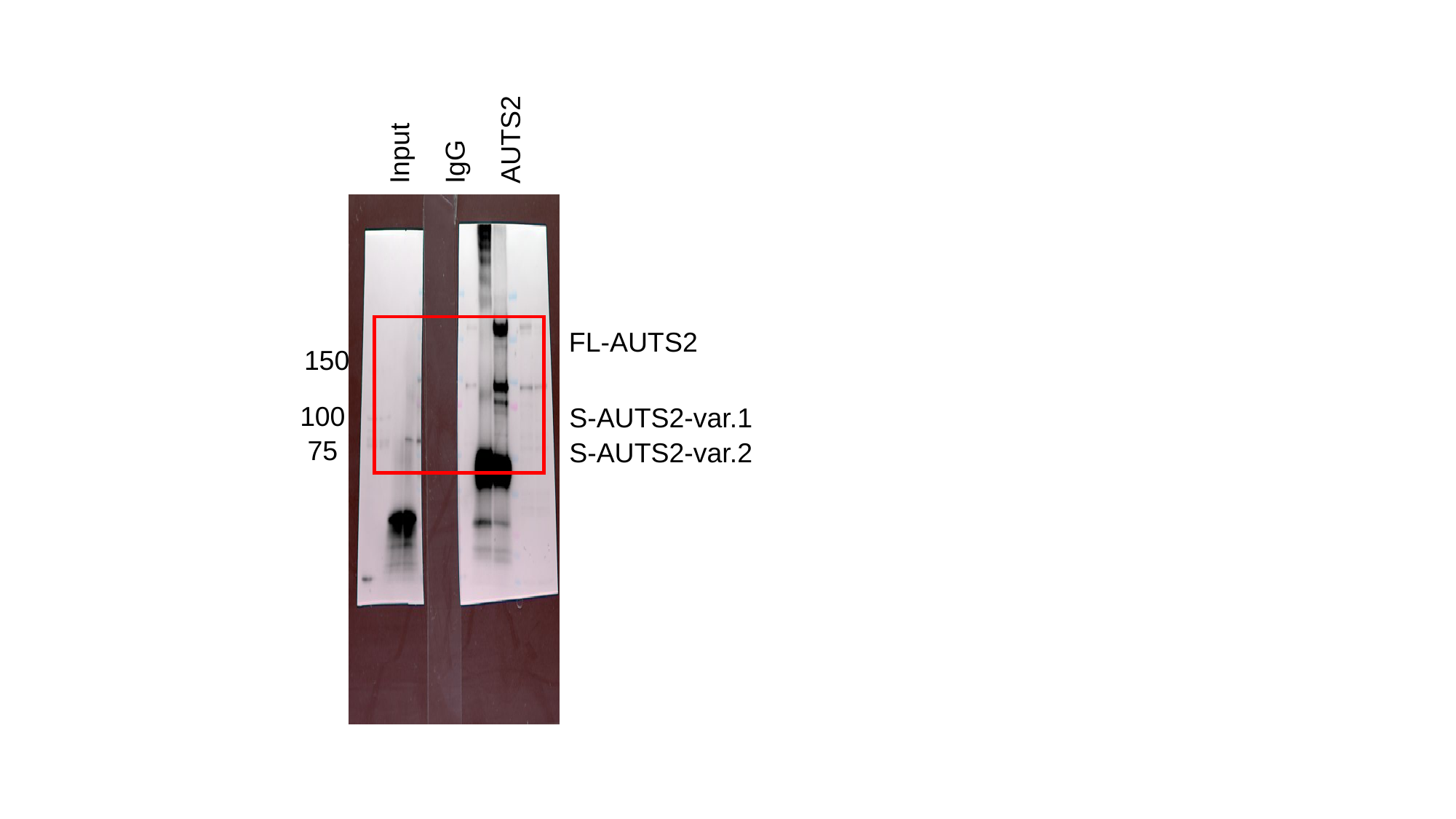

AUTS2
Input
IgG
FL-AUTS2
150
100
S-AUTS2-var.1
75
S-AUTS2-var.2

Supplement: Supplementary file 11 — Source data Fig. 8 [file 44318_2024_343_MOESM11_ESM.zip › Figure8/8B/AUTS2.pptx]

## Slide 1
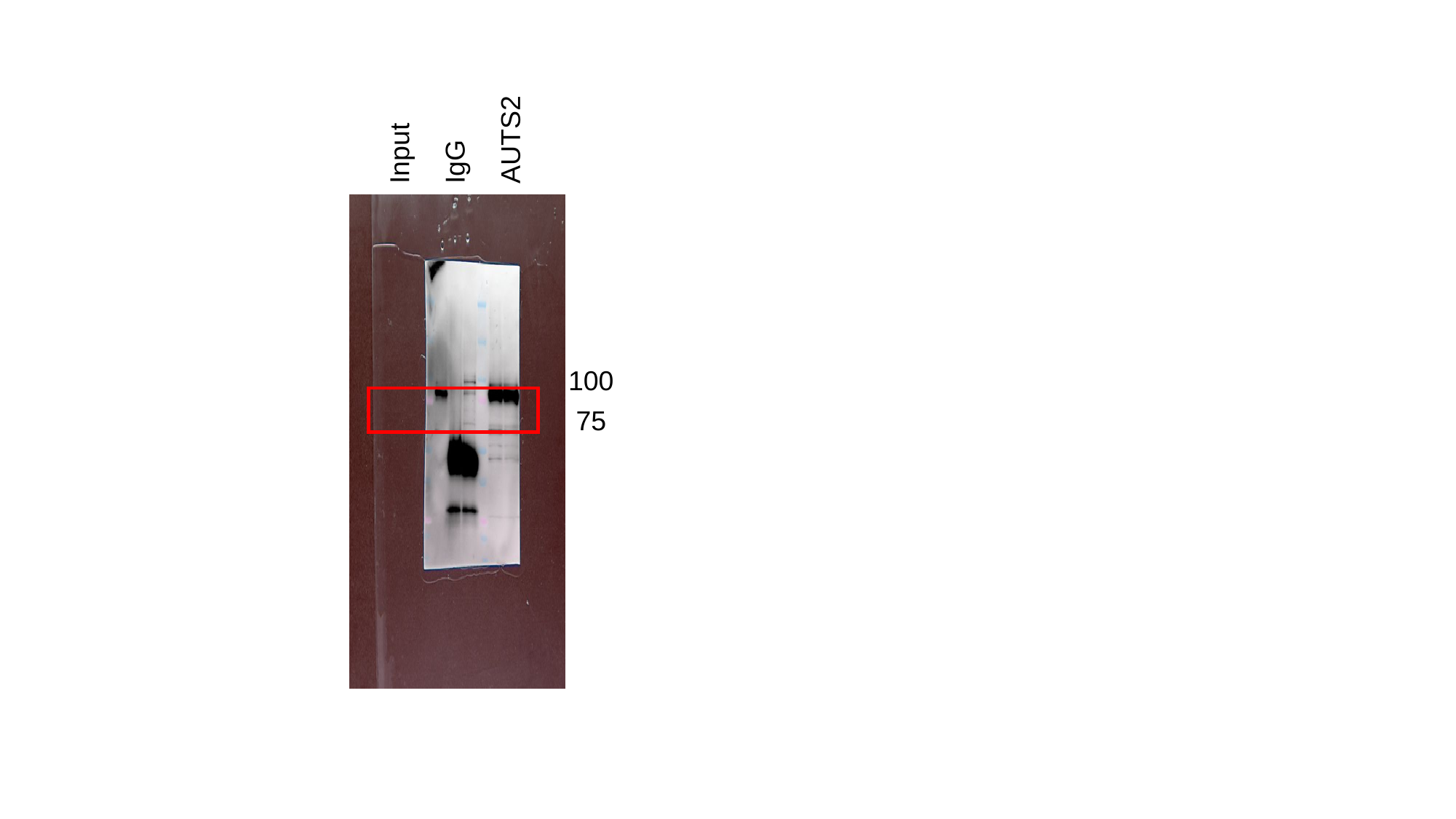

AUTS2
Input
IgG
100
75

Supplement: Supplementary file 11 — Source data Fig. 8 [file 44318_2024_343_MOESM11_ESM.zip › Figure8/8B/SUZ12.pptx]

## Slide 1
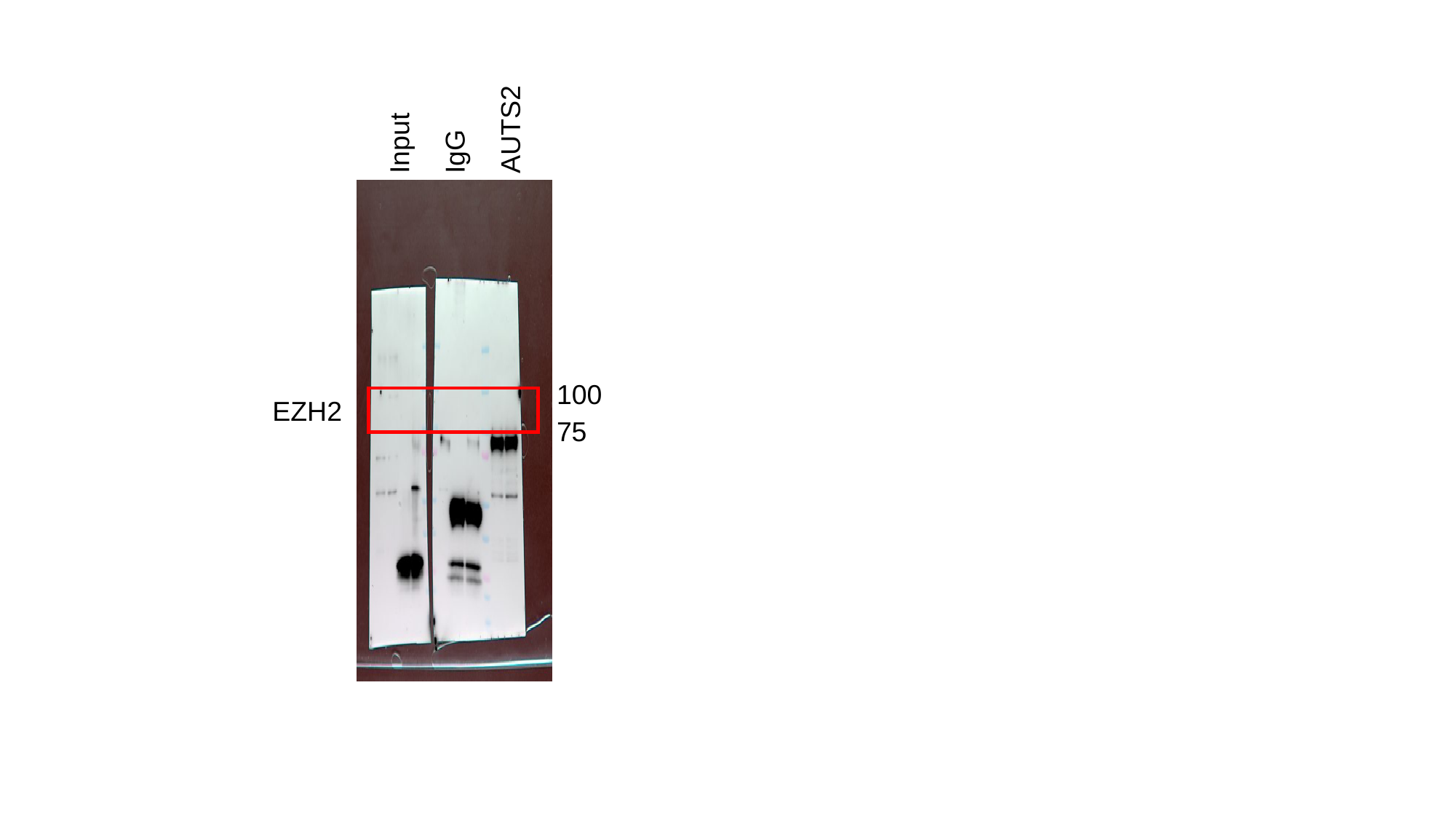

AUTS2
Input
IgG
100
EZH2
75

Supplement: Supplementary file 11 — Source data Fig. 8 [file 44318_2024_343_MOESM11_ESM.zip › Figure8/8B/EZH2.pptx]
